# Bridging the Gap Between Morphometric Similarity Mapping and Gene Transcription in Alzheimer’s Disease

**DOI:** 10.3389/fnins.2021.731292

**Published:** 2021-09-29

**Authors:** Yang Zhang, Min Ma, Zhonghua Xie, Heng Wu, Nan Zhang, Junlin Shen

**Affiliations:** ^1^Department of Medical Imaging and Tianjin Key Laboratory of Functional Imaging, Tianjin Medical University General Hospital, Tianjin, China; ^2^Department of Mathematics, School of Science, Tianjin University of Science and Technology, Tianjin, China; ^3^Tianjin Key Laboratory of Lung Cancer Metastasis and Tumor Microenvironment, Tianjin Lung Cancer Institute, Tianjin Medical University General Hospital, Tianjin, China; ^4^Department of Neurology, Tianjin Medical University General Hospital, Tianjin, China

**Keywords:** Alzheimer’s disease, morphometric similarity, Allen Human Brain Atlas, gene transcription, sMRI = structural MRI

## Abstract

Disruptions in brain connectivity have been widely reported in Alzheimer’s disease (AD). Morphometric similarity (MS) mapping provides a new way of estimating structural connectivity by interregional correlation of T1WI- and DTI-derived parameters within individual brains. Here, we aimed to identify AD-related MS changing patterns and genes related to the changes and further explored the molecular and cellular mechanism underlying MS changes in AD. Both 3D-T1WI and DTI data of 106 AD patients and 106 well-matched healthy elderly individuals from the ADNI database were included in our study. Cortical regions with significantly decreased MS were found in the temporal and parietal cortex, increased MS was found in the frontal cortex and variant changes were found in the occipital cortex in AD patients. Mean MS in regions with significantly changed MS was positively or negatively associated with memory function. Negative MS-related genes were significantly downregulated in AD, specifically enriched in neurons, and participated in biological processes, with the most significant term being synaptic transmission. This study revealed AD-related cortical MS changes associated with memory function. Linking gene expression to cortical MS changes may provide a possible molecular and cellular substrate for MS abnormality and cognitive decline in AD.

## Introduction

Alzheimer’s disease (AD) is a neurodegenerative disease marked by progressive neuron loss, manifested by short-term memory and other cognitive impairment symptoms (Wang et al., 2020). AD-related neurodegeneration involves several brain regions, in which the entorhinal, hippocampal and temporal cortices are the most reported (Lerch et al., 2005; Im et al., 2008; Morra et al., 2008; Seong et al., 2010; Li et al., 2014; Femminella et al., 2018). Structural indicators of these regions, including gray matter density (Frisoni et al., 2002), volume (Busatto et al., 2003), cortical thickness (Pettigrew et al., 2017), and curvature (Im et al., 2008; Seong et al., 2010), have been found to be decreased in AD patients. White matter studies based on diffusion tensor imaging (DTI) have also demonstrated reduced integrity in the temporal lobe as well as white matter tracts connecting frontal and temporal regions in AD (Naggara et al., 2006; Kantarci et al., 2017). In recent years, AD has been widely regarded as a disconnected syndrome whereby a large-scale brain network is progressively disrupted by neuropathological processes. MR topological studies constructed whole-brain structural networks and demonstrated abnormal topological properties in multiple brain regions, including the hippocampal, frontal, temporal, parietal and occipital regions, verifying brain network disruption and disconnection between anatomically connected brain regions in AD patients (Lo et al., 2010; Yao et al., 2010).

All the above-mentioned multiregional changes in either gray matter (from 3D T1WI) or white matter (from DTI) may be attributed to dysconnectivity of large-scale brain structural networks in AD. However, a structural covariance network using T1WI could not be applied to single-subject level analysis, and precisely estimating long-distance connections still constrains DTI-based tractography. Here, we adopted a different parameter from the past—“morphometric similarity (MS)”—which is estimated as the inter-regional correlation of multiple macro- and micro-structural multimodal MRI variables, based on both structural T1WI and DTI (Stam et al., 2007). It reflects the anatomical connections of different brain areas from histological similarity and axonal connectivity within an individual human brain (Seidlitz et al., 2018). Given that AD has been considered a disconnection syndrome due to regional vulnerability to cellular neurodegeneration and disconnection of distant cortical regions (Gonzalez-Escamilla et al., 2020), it is suitable to evaluate brain anatomical connectivity in AD patients using MS as a neuroimaging indicator.

AD is a highly heritable disease (Bellenguez et al., 2020). Investigating the link between related gene expression and internal brain structure helps to understand the pathophysiological processes of the disease. The Allen Human Brain Atlas (AHBA) can present gene transcription information in the same standard space as neuroimaging data, providing a new approach for linking gene expression to neuroimaging phenotypes. With this approach, only a few reports combine gene transcription data with gray matter volumes in AD. However, it is unclear which genes related to AD-specific MS changes are specific to which neurological functions and how the expression of these genes affects MS changes. In the current study, we investigated the MS changing pattern map in AD and spatially associated the MS changing pattern map with anatomically patterned gene expression using data from the AHBA. We aimed to identify AD-related MS changing patterns and genes closely related to the changes and further explore the cellular and molecular mechanism underlying MS changes in AD.

## Materials and Methods

### Participates

A total of 113 AD patients with their initial 3T MRI scans, including both 3D T1WI and DTI data, were obtained from ADNI database^1^ which followed the standard ADNI-GO and ADNI-2 protocols (Jack et al., 2010; Weiner et al., 2017). The main inclusion criteria were as follows: (1). subjective memory concern as reported by subject, study partner or clinician; (2). abnormal memory function documented by scoring within the education adjusted ranges on the Logical Memory II subscale from the Wechsler Memory Scale-Revised; (3). Mini-Mental State Exam (MMSE) score between 20 and 26; (4). Clinical Dementia Rating 0.5 or 1.0; and 5. NINCDS/ADRDA criteria for probable AD. All images were visually inspected by two radiologists, and seven patients with poor image quality (2 patients’ 3D T1WI and five patients’ DTI) were excluded. Finally, 106 AD patients with qualified image data were included (63 males and 43 females; mean age 75, ranging from 55 to 90 years). For comparison, an equal number of age- and gender-matched healthy elders with qualified 3D-T1WI and DTI were selected from the ADNI database (63 males and 43 females; mean age 75, ranging from 55 to 90 years). The detailed scan parameters are provided in Supplementary Table 1. General cognitive function was assessed by the MMSE and the Clinical Dementia Rating. Memory function was evaluated by a memory composite score obtained for the majority of participants (94 subjects with AD and 99 subjects with healthy elderly individuals) (Crane et al., 2012).

### Morphometric Similarity Estimation

Surface-based morphology parameter estimation from high-resolution T1WI was performed using FreeSurfer v6.0.0.^2^ The DTI data were preprocessed according to the pipeline of FMRIB’s Diffusion Toolbox implemented in FSL 5.0.10.^3^ The detailed preprocessing procedures for T1WI and DTI data are provided in Supplementary Material.

The DTI parameters of fractional anisotropy and mean diffusion were defined as myelination metrics. Among the surface-based morphology parameters, the gray matter, surface area and cortical thickness were defined as gray matter metrics, and the intrinsic/Gaussian curvature and mean curvature were the curvature metrics.

To adjust the variation from multiple sites and scanners, the ComBat harmonization of surface-based morphology and diffusion parameters across scanners and sites was performed before the downstream morphometric similarity estimation (Fortin et al., 2017, 2018). Then, these metrics were Z-score transformed to improve normality.

The Pearson correlation of gray matter, curvature and myelination metrics between each pair of cortical regions was performed to generate 308 × 308 MS matrices for each subject. Then, the 308 × 308 MS matrices were averaged across the 308 cortical regions to calculate the regional MS for every 308 cortical regions. From the brain connectome perspective, the regional MS represents the weighted degree of each cortical node, which was connected by signed and weighted edges of pairwise similarity to all other cortical nodes in the whole brain.

### Transcription-Imaging Association

A compiled transcription matrix of six postmortem adult brains from the AHBA^4^ was acquired from the data directory for Neuroscience in Psychiatry Network manuscript,^5^ which provided expression values for each of 20,737 genes estimated in 151 cortical regions of the left hemisphere. PLS regression was used to identify genes whose transcriptional profiles were significantly associated with regional MS differences. In this study, the independent variable was the compiled AHBA transcription matrix (151 regions × 20,737 genes), and the dependent variables were the vector of regional MS case-control *T-*values from the left hemisphere (151 regions). The first PLS component (PLS1) weight of each gene was assigned in terms of its contribution to the overall model. Then, the ratio of each gene’s PLS1 weight to its bootstrapped standard error (1,000 resamplings with replacement of the 151 cortical regions) was calculated as a *Z* score. Here, genes with | *Z* score | > 4.72 (Bonferroni correction of *P* < 0.05) denoted the PLS1 gene set. Details about the transcription-imaging association are provided in Supplementary Material.

### Disease Enrichment Analyses

Disease enrichment analyses were used to explore whether the PLS1 gene set was enriched in AD-related differentially expressed genes (DEGs). The expression dataset with series accession number GSE5281 from the Gene Expression Omnibus database^6^ was acquired to screen the AD-related DEGs. The LIMMA package (version 3.42.2) of R software was used to analyze the DEGs between AD and normal elderly individuals. *P* < 0.01 and | log_2_ (fold change) | > 1 were defined as the thresholds for screening AD-related DESs. Fisher’s exact test was used to evaluate the significance of the overlap between PLS1 gene sets and AD-related DEGs. The Bonferroni method was used to correct for multiple comparisons (both up- and downregulated DEGs) (*Pc* < 0.05, an uncorrected *P* < 0.05/2 = 0.025). Details about the disease enrichment analyses are provided in Supplementary Material.

### Cell-Type-Specific Analysis

The RNAseq dataset with series accession number GSE73721 from the Gene Expression Omnibus database was acquired to perform cell-type-specific analysis. pSI v1.1^7^ was used to determine the specific neocortical cell type for which the PLS1-genes were enriched. A pSI threshold of 0.05 was used to generate the cell-type-enriched gene lists for each type of cortical cell. Fisher’s exact test was used to evaluate the significance of the overlap between PLS1 gene sets and cell-type-specific genes for each type of cortical cell. The Bonferroni method was used to correct for multiple comparisons (5 cell types) (*Pc* < 0.05, an uncorrected *P* < 0.05/4 = 0.01). Details about the cell type-specific analysis are provided in Supplementary Material.

### Gene Ontology Analysis

The clusterProfiler package (v3.14.3) of R software was used to perform the gene ontology (GO) analysis. Our study only focused on the biological process of GO terms in which the PLS1 gene sets were enriched. A Bonferroni adjusted *P*-value < 0.05 was considered significant.

### Statistical Analysis

The statistical analyses for demographic and cognitive data were performed using the Statistical Package for the Social Sciences (SPSS version 18.0). Comparisons between AD patients and healthy elderly individuals were performed using a two-sample *T*-test for continuous variables with a normal distribution and a chi-squared test for categorical variables. To test whether the MS of brain regions with significant case-control differences were associated with memory function, partial correlation analysis was conducted with age, gender, and years of education as nuisance covariates. The Pearson correlation analysis was used to test the association between the *Z* scored expression values of PLS1 gene sets and the *T* statistics of case-control differences in MS. The resulting *P*-values above were Bonferroni corrected for multiple comparisons. The case-control difference in regional MS was estimated by fitting linear models with age, gender and education as covariates, and the resulting *P*-values for each region were false discovery rate (FDR) corrected for multiple comparisons.

## Results

### Demographics and Cognition

A total of 106 AD patients and the same number of age- and gender-matched healthy elderly individuals with qualified image data were ultimately included in the present study. The demographic and cognitive data of these subjects are shown in Table 1. Significant differences were found in terms of MMSE (*P* = 0.0001), Clinical Dementia Rating (*P* = 0.0001), and memory composite scores (*P* = 0.0001). No significant differences were observed in terms of age (*P* = 0.98), gender (*P* = 1), or years of education (*P* = 0.06).

**TABLE 1:** Demographics and cognition.

	AD (*n* = 106)	NC (*n* = 106)	*T*/χ^2^	*P*-value
Age, years	74.94 ± 8.02	74.92 ± 7.84	0.026	0.979
Education, years	15.59 ± 2.60	16.27 ± 2.50	−1.92	0.06
Gender, male/female	63/43	63/43	0	1
MMSE	22.92 ± 3.13	28.57 ± 1.73	−16.26	0.0001
CDR	0.81 ± 0.27	0.04 ± 0.13	26.13	0.0001
Memory composite score*	−0.85 ± 0.50	0.79 ± 0.54	−22.01	0.0001

The data are shown as means (SD). The symbol * indicates that the composite memory score was available from 94 of the 106 AD and 99 of the 106 NC. AD, Alzheimer’s disease; CDR, Clinical Dementia Rating; MMSE, Mini-Mental State Examination; NC, normal control subject.

### Morphometric Similarity Differences Between Alzheimer’s Disease and Healthy Elders

**FIGURE 1:**
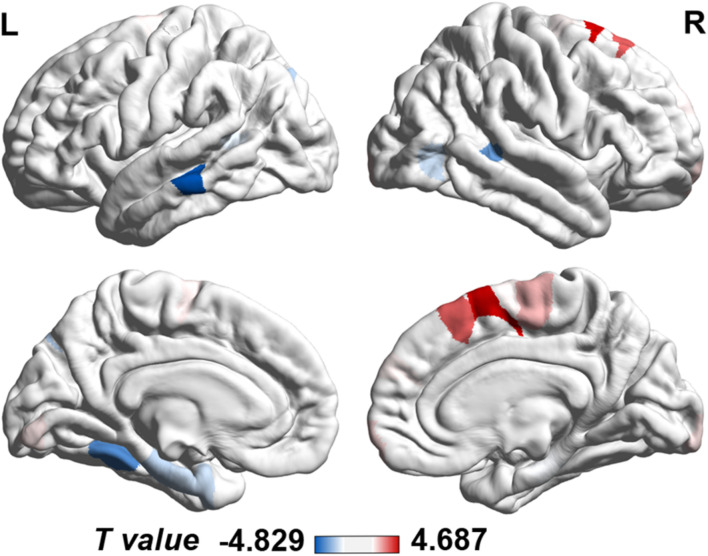
Case-control differences in regional morphometric similarity (*P* < 0.05, FDR corrected). Regions in blue indicate significantly decreased morphometric similarity in AD, whereas regions in red indicate significantly increased morphometric similarity in AD. FDR, false discovery rate; L, left; R, right.

**FIGURE 2:**
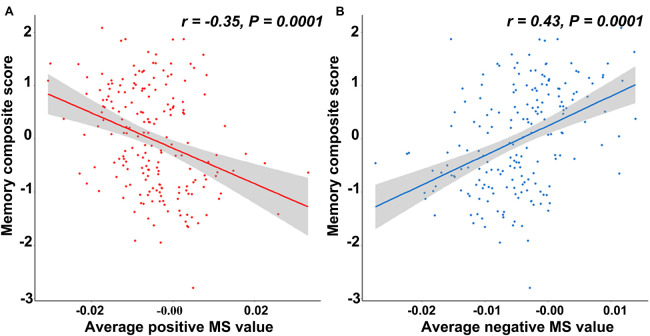
The relationship between the memory composite score and regional morphometric similarity. **(A)** The average morphometric similarity in regions with significantly increased morphometric similarity in AD is significantly negatively correlated with the memory composite score. **(B)** The average morphometric similarity in regions with significantly decreased morphometric similarity in AD is significantly positively correlated with the memory composite score.

The cortical map in Figure 1 demonstrated the significant differences in regional MS at each cortical area between AD and healthy elderly individuals. Cortical regions with significantly decreased MS were observed in the left middle temporal lobe, left fusiform gyrus, bilateral banks of superior temporal sulci, bilateral parahippocampal lobes, left entorhinal cortex, left superior parietal lobe, left supramarginal gyrus and right lateral occipital lobe (Table 2). Cortical regions with significantly increased MS were found in the bilateral superior frontal lobes, right paracentral lobe, right frontal pole cortex, left lingual gyrus and right lateral occipital lobe (Table 2). The partial correlation analysis showed that the mean MS average across the 10 regions with decreased MS was significantly positively associated with the memory composite score (*r* = 0.43, *P* = 0.0001), and the mean MS average across the nine regions with increased MS was significantly negatively associated with the memory composite score (*r* = −0.35, *P* = 0.0001) (Figure 2).

**TABLE 2:** Cortical regions of case-control differences in regional morphometric similarity.

Cortical regions	Coordinates (MNI)			*T* value	*P*-value	FDR
	x	y	z			
L_middletemporal_part5	–60.019	–27.635	–13.299	–4.8294	2.66E-06	7.74E-04
L_fusiform_part1	–30.238	–46.494	–17.452	–4.2592	3.11E-05	3.196E-04
R_bankssts_part1	53.969	–39.123	1.4973	–4.1341	5.18E-05	3.986E-03
L_superiorparietal_part8	–23.65	–73.056	29.861	–3.5866	4.18E-04	0.021429
L_parahippocampal_part1	–25.991	–25.187	–25.332	–3.5065	5.57E-04	0.021429
R_lateraloccipital_part2	44.513	–70.022	–2.0359	–3.4184	7.59E-04	0.024105
L_entorhinal_part1	–24.011	–5.8614	–32.827	–3.4094	7.83E-04	0.024105
L_bankssts_part2	–53.141	–49.843	8.2646	–3.2373	0.001405	0.035229
R_parahippocampal_part2	27.448	–24.861	–24.205	–3.0702	0.002426	0.04151
L_supramarginal_part7	–49.357	–38.912	32.554	–3.0206	0.00284	0.046042
R_superiorfrontal_part7	9.6868	8.2947	60.026	4.6868	5.03E-06	7.74E-04
R_superiorfrontal_part11	9.2005	24.389	53.686	4.0632	6.87E-05	4.232E-03
R_paracentral_part2	5.3566	–16.772	61.135	3.5071	5.56E-04	0.021429
R_frontalpole_part1	9.8338	62.819	–10.737	3.285	0.0011976	0.033534
R_lateraloccipital_part1	18.646	–99.162	–7.394	3.2203	0.001487	0.035229
L_lingual_part2	–6.5596	–88.407	–8.0452	3.1896	0.001646	0.036218
L_superiorfrontal_part2	–11.687	–8.4248	64.785	3.1488	0.001882	0.038635
R_superiorfrontal_part6	10.21	54.909	26.16	3.1191	0.002073	0.039895
R_superiorfrontal_part3	12.367	–3.2651	65.643	3.0906	0.002272	0.041168

*The cortical regions above the middle line of the table are regions with significantly decreased morphometric similarity in AD, whereas the cortical regions under the middle line of the table are regions with significantly increased morphometric similarity in AD. FDR, the corrected P-value with the false discovery rate method.*

### Gene-Morphometric Similarity Spatial Correlations and Characters

#### First PLS Component Gene Expression Associated With Morphometric Similarity Difference

**FIGURE 3:**
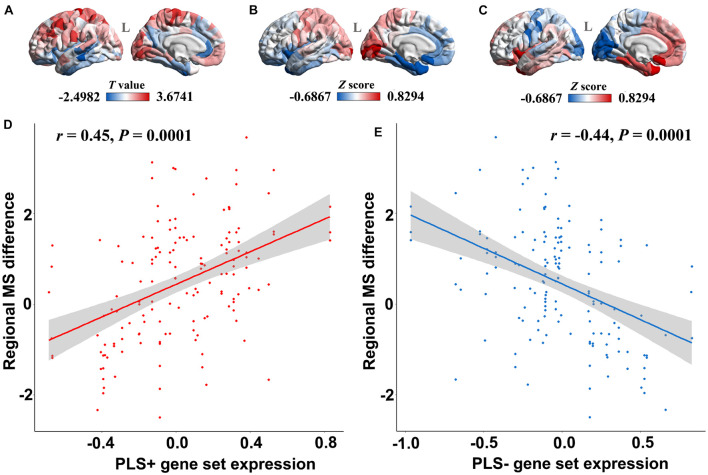
The relationship between PLS1 gene set expression and regional morphometric similarity differences. **(A)** The regional morphometric similarity case-control *T* map in the left hemisphere. The regions in red indicate increased morphometric similarity in AD, whereas the blue color indicates decreased morphometric similarity in AD. **(B)** The PLS1 + gene set expression map illustrates that regions in red have increased expression of the PLS1 + gene set, whereas regions in blue have decreased expression of the PLS1 + gene set. **(C)** The PLS1-gene set expression map illustrates that regions in red have increased expression of the PLS1-gene set, whereas regions in blue have decreased expression of the PLS1-gene set. **(D)** The point plot in red shows that the expression of the PLS1 + gene set is significantly positively correlated with the regional morphometric similarity difference between AD patients and healthy elderly individuals. **(E)** The blue point plot shows that the expression of the PLS1-gene set is significantly negatively correlated with regional morphometric similarity differences between AD patients and healthy elderly individuals.

The PLS regression analysis revealed 1,932 genes with normalized PLS1 weights *Z* score < −4.72 (Bonferroni correction of *P* < 0.05), which were defined as the PLS1-genes, and 2,139 genes with normalized PLS1 weights *Z* score > 4.72 (Bonferroni correction of *P* < 0.05), which were defined as the PLS1 + genes (Supplementary Table 2). The majority of cortical regions on the PLS1 + gene expression map were in accordance with those on the case-control *T* map of regional MS (Figures 3A,B), whereas the majority of cortical regions on the PLS1-gene expression map were in contrast with those on the case-control *T* map of regional MS (Figures 3A–C). Pearson correlation analysis revealed that the expression of PLS1 + genes was significantly positively correlated with regional MS differences (*r* = 0.45, *P* = 0.0001) (Figure 3D), whereas the expression of PLS1-genes was significantly negatively correlated with regional MS differences (*r* = −0.44, *P* = 0.0001) (Figure 3E).

#### Alzheimer’s Disease-Related Differentially Expressed Genes Enrichment for First PLS Component-Genes

**FIGURE 4:**
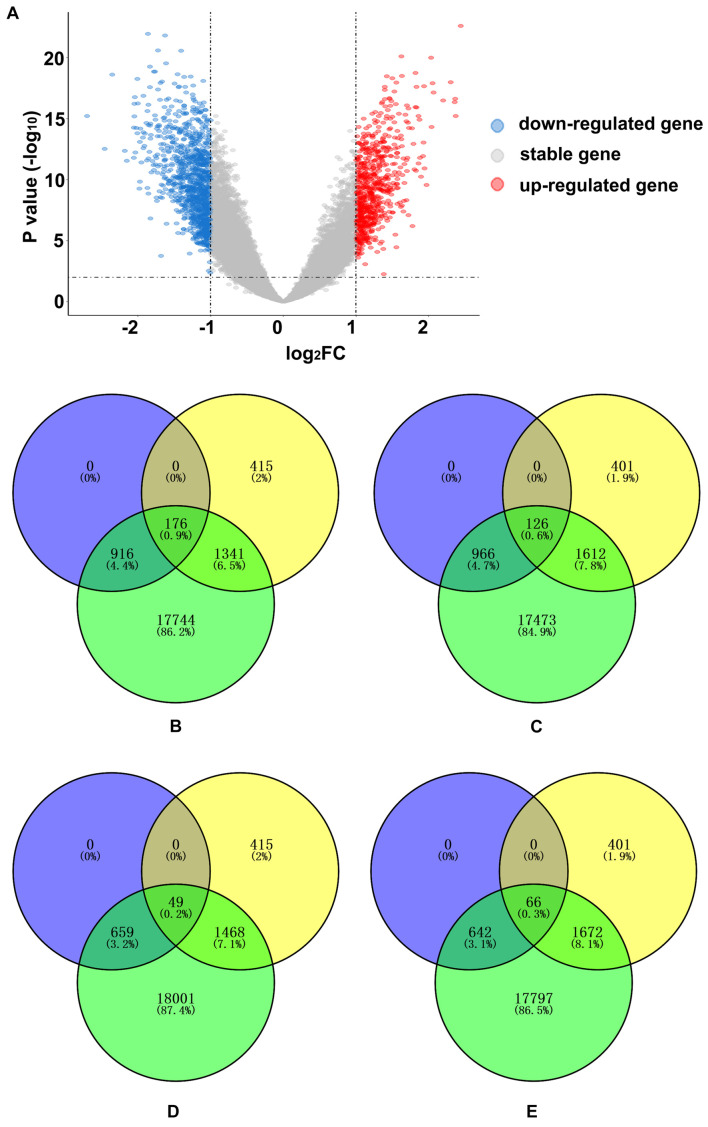
AD-related DEG enrichment analyses for PLS1 gene sets. **(A)** The volcano plot shows 708 upregulated genes in red on the right and 1,092 downregulated genes in blue on the left. **(B)** The PLS1-genes significantly overlapped with downregulated genes. The number of overlapping genes was 176, accounting for 0.9% of the total genes. The purple circle indicates 1,092 downregulated genes, the yellow circle indicates 1,932 PLS1-genes and the green circle indicates 20,177 background genes. **(C)** The PLS1 + genes did not significantly overlap with downregulated genes. The number of overlapping genes was 126, accounting for 0.6% of the total genes. The purple circle indicates 1,092 downregulated genes, the yellow circle indicates 2,139 PLS1 + genes and the green circle indicates 20,177 background genes. **(D)** The PLS1-genes did not significantly overlap with the upregulated genes. The number of overlapping genes was 49, accounting for 0.2% of the total genes. The purple circle indicates 708 up-regulated genes, the yellow circle indicates 1,932 PLS1-genes, and the green circle indicates 20,177 background genes. **(E)** The PLS1 + genes did not significantly overlap with upregulated genes. The number of overlapping genes was 66, accounting for 0.3% of the total genes. The purple circle indicates 708 upregulated genes, the yellow circle indicates 2,139 PLS1 + genes and the green circle indicates 20,177 background genes. DEGs, differentially expressed genes; FC, fold change; PLS, partial least squares regression.

A total of 1,800 significant DEGs between AD and normal elderly individuals were identified from the GSE5281 series, with 708 upregulated and 1,092 downregulated genes (Figure 4A and Supplementary Table 3). Both upregulated and downregulated genes were defined as AD-related DEGs. The enrichment analysis revealed that PLS1-genes were significantly enriched in downregulated DEGs (*Pc* = 5.43 × 10^–12^) but not in upregulated DEGs (*Pc* = 1) (Figures 4B–D). In addition, PLS1 + genes were not significantly enriched in upregulated DEGs (*Pc* = 1) or in downregulated DEGs (*Pc* = 1) (Figures 4C–E).

#### Cell-Type Specificity of First PLS Component-Genes

**FIGURE 5:**
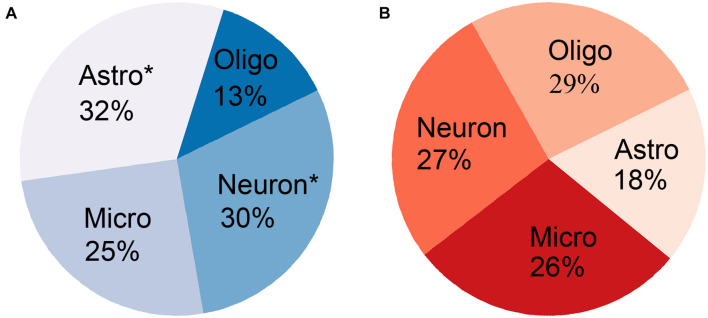
The percentage of gene ratio between overlapping genes and cell-type-specific genes for each type of neocortical cell. **(A)** The PLS1-genes significantly overlapped with cell-type-specific genes in neurons and astrocytes (asterisk) but not in oligodendrocytes and microglia. **(B)** The PLS1 + genes did not significantly overlap with cell-type-specific genes in any type of neocortical cell.

The cell-type-enriched gene lists for each type of cortical cell are provided in Supplementary Table 4. The PLS1-genes showed significant specific expression in neurons (*Pc* = 1.83 × 10^–5^) and astrocytes (*Pc* = 1.74 × 10^–5^) but not in oligodendrocytes (*Pc* = 1) or microglia (*Pc* = 0.57) (Table 3 and Figure 5A). The PLS1 + genes were not significantly enriched in any type of neocortical cell (*Pc* = 1 for all) (Table 4 and Figure 5B).

**TABLE 3:** The significance of the overlap between PLS1-genes and cell-type-specific genes.

	Astrocytes	Neurons	Oligodendrocytes	Microglia
Overlapped genes	139	195	33	71
Cell-type-specific genes	1,160	1,770	684	746
Gene ratio	0.12	0.11	0.048	0.095
*Pc* values	1.83 × 10^–5^	1.74 × 10^–5^	1	0.57

*PLS1, the first component of partial least squares regression; Overlapped genes, the number of overlapping genes between PLS1-genes and cell-type-specific genes; Gene ratio, gene ratio between the number of overlapping genes and the number of cell-type-specific genes; Pc values, the Bonferroni corrected P-values.*

**TABLE 4:** The significance of the overlap between PLS1 + genes and cell-type-specific genes.

	Astrocytes	Neurons	Oligodendrocytes	Microglia
Overlapped genes	67	153	62	61
Cell-type-specific genes	1,160	1,770	684	746
Gene ratio	0.058	0.086	0.091	0.082
*Pc* values	1	1	1	1

*PLS1, the first component of partial least squares regression; Overlapped genes, the number of overlapping genes between PLS1 + genes and cell-type-specific genes; Gene ratio, ratio between the number of overlapping genes and the number of cell-type-specific genes; Pc values, the Bonferroni corrected P-values.*

#### Gene Ontology Enrichment for First PLS Component-Gene Sets

**FIGURE 6:**
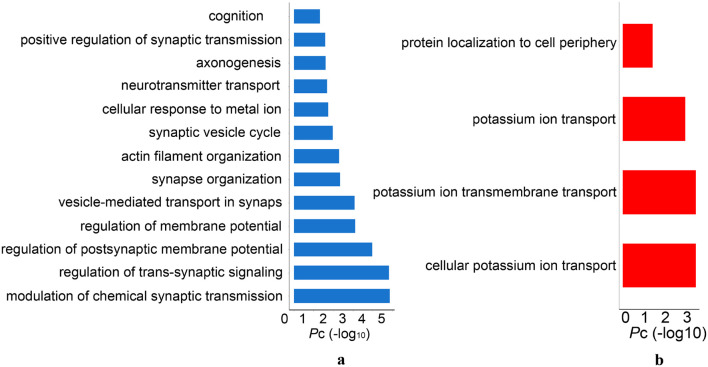
The significant gene ontology terms of biological process for PLS1 gene sets. **(A)** The PLS1-genes were significantly enriched in synaptic signaling, neurotransmitter release, axonogenesis and cognition. **(B)** The PLS1 + genes were significantly enriched in potassium ion transport and protein localization. *Pc*, the Bonferroni corrected *P*-values.

The GO analysis revealed that significant biological processes of the PLS1-genes were mainly enriched in neuron-specific terms, including synaptic signaling, neurotransmitter release, axonogenesis, and cognition (Figure 6A and Supplementary Table 5). However, the PLS1 + genes were involved in non-neuron-specific biological processes, including potassium ion transport and protein localization (Figure 6B and Supplementary Table 5).

## Discussion

### Morphometric Similarity Changing Patterns and Associated Memory Function in Alzheimer’s Disease

The MS quantifies the similarity in terms of multiple MRI parameters measured in each area. Compared with traditional measurements based on a single MRI sequence, MS considering multiple MRI morphometric indices (based on both structural T1WI and DTI) could reflect the anatomical connections of different brain areas based on histological similarity and axonal connectivity within an individual human brain.

This study showed that AD patients had decreased regional MS in multiple AD-susceptible regions in the temporal and parietal cortex. Additionally, increased regional MS in several frontal areas and variable changing MS in parts of the occipital cortex were also detected in AD patients compared with healthy elderly individuals. The mean MS average across those regions with decreased regional MS was positively associated with memory function. In contrast, the mean MS average across those regions with increased regional MS was negatively associated with memory function.

Our findings were consistent with a large number of studies reporting decreased gray matter volume and cortical thickness (Lerch et al., 2005; Femminella et al., 2018), lower average mean curvature (Im et al., 2008; Morra et al., 2008) and shallower sulcal depth (Im et al., 2008) in the hippocampus, temporal lobe, fusiform gyrus, and entorhinal cortex in AD, with the left hemisphere being dominant. DTI studies revealed disruptions of white matter integrity in the early stage of AD in limbic fiber tracks with direct connections to medial temporal lobe structures (Kalus et al., 2006; Zhang et al., 2007; Sexton et al., 2010). Moreover, decreased connectivity of multiple brain regions, including the temporal lobe, hippocampus, fusiform gyrus and parietal lobe, has also been documented as the cause of cognitive decline in AD patients (Bokde et al., 2006; Stam et al., 2007; He et al., 2008). Decreased MS in multiple brain regions, including the temporal, parietal and part of the occipital cortical regions in AD, reflected the weakening of the abovementioned brain regions’ anatomical connections from the histological and cellular architecture level and implied increased architectonic differentiation and decreased axonal connectivity between these cortical regions. We further found a correlation between the weakening of this anatomical connection and the impairment of memory function, suggesting that the anatomical disconnection caused by the reduction of the similarity of histology and cellular architecture may be the neural basis for the impairment of memory function in AD patients.

Our result of elevated MS in the prefrontal areas and the left lingual gyrus in AD patients suggested increased architectonic similarity and enhanced axonal connectivity in these regions in AD patients. These findings were consistent with enhanced functional activation and connectivity within frontal regions in the early stage of AD (Grady et al., 2003; Aganj et al., 2020). In addition, at the local network level, changes in connectivity of the left lingual gyrus were also reported to be significantly negatively correlated with behavioral performance in AD patients (Chang et al., 2020). We tended to interpret the increased prefrontal and lingual MS in AD as a structural compensatory reallocation of cognitive resources. This explanation was further supported by the negative correlation between the average MS value of the brain areas and increased MS and memory function in AD patients. The more structural compensatory the increase in MS is, the worse the performance of the memory function is. As the disease becomes severe, the structural compensatory increase may disappear, but this needs to be confirmed by future longitudinal studies. Regarding the occipital areas, most AD studies have reported atrophy, hypometabolism (Ten Kate et al., 2018; Das et al., 2021) and connection changes (Huang et al., 2020) in this area in AD patients. Studies have also found an up-regulated signaling pathway located in the occipital area in AD patients, which suggests that an enhancement in dying or surviving neurons plays a protective role by compensating for decreased neurotransmission during the progression of AD (Jacobs et al., 2006). The inconsistent MS change patterns in the occipital areas in AD patients in the current study may be related to different functional areas with distinct changing patterns in the occipital lobe, which was supported by the evidence of the dissociation between impaired explicit memory encoding in secondary visual areas and intact implicit encoding in the primary visual cortex in AD.

### Linking Gene Expression to Morphometric Similarity Difference Map and Functional Annotation

PLS analysis showed that the PLS1 + gene was positively correlated with the AD-related MS difference map, and the PLS1-gene was negatively correlated with the AD-related MS difference map. However, only PLS1-genes were significantly enriched in downregulated AD-related DEGs. GO analysis and cell-type-specific analysis showed that the PLS-genes were cytologically enriched in neurons and astrocytes and functionally involved in neuron-specific biological processes, including synaptic signaling, neurotransmitter release, axonogenesis, and cognition. Because PLS1 + genes were not enriched in AD differential genes and implicated in non-neuron-specific functions, the following discussion mainly focuses on the PLS1-genes.

The circuitry of the human brain is formed by neuronal networks in which astrocytes embed. Synaptic signaling, neurotransmitter release and axonogenesis are fundamental to highly efficient neuronal networks, which maintain normal cognition in humans (Verkhratsky et al., 2010). The loss of neurons and synapses and axon destruction are common findings in AD neuropathology and are related to cognitive decline in AD patients. Exposure of astrocytes to Aβ may induce astrocyte activation (Diniz et al., 2017) and release proinflammatory cytokines, contributing to neuronal death (Wood et al., 2015). As PLS1-genes were significantly enriched in downregulated AD-related DEGs, it can be presumed that the reduced expression of PLS1-genes may lead to neuron death, axon deterioration and synapse loss, causing histological similarity and anatomical connectivity destruction and, thus, abnormal MS changes in AD.

The PLS1-genes acted as a whole gene set in the enrichment analysis for AD-related DEGs, cortical cell types and GO terms. We cannot ensure that every single gene in the PLS1-gene set was enriched in AD-related DEGs, cortical cell types and GO terms simultaneously. The significance of the enrichment analysis did not represent for the true biological connection. Further *in vitro* and *in vivo* experiments are warranted to validate our hypothesis. Although variation from multiple sites and scanners could be moderately adjusted using ComBat harmonization, different scan protocols still affected the results. More robust methods are needed in the future to properly control the batch effect from multiple sites and scanners.

In summary, this study revealed AD-related cortical MS changes associated with memory function. Linking gene expression to cortical MS changes, the negative MS-related genes were found to be enriched explicitly in neurons and astrocytes, participate in neuron-specific biological processes and be significantly downregulated in AD. These findings may provide a possible molecular and cellular substrate for MS abnormalities and cognitive decline in AD.

## Data Availability Statement

The original contributions presented in the study are included in the article/Supplementary Material, further inquiries can be directed to the corresponding author/s.

## Ethics Statement

The Data used in this study were obtained from the Alzheimer’s Disease Neuroimaging Initiative (ADNI) (http://adni.loni.usc.edu/). The ADNI data were previously collected across 50 research sites. Study subjects gave written informed consent at the time of enrollment for imaging and genetic sample collection and completed questionnaires approved by each participating sites’ Institutional Review Board (IRB). The list of all sites can be found in Supplementary Material, Data Sheet 2. All procedures performed in studies involving human participants were in accordance with the ethical standards of the institutional and/or national research committee and with the 1964 Helsinki declaration and its later amendments or comparable ethical standards. The patients/participants provided their written informed consent to participate in this study.

## Author Contributions

JS and YZ conceived the idea. MM and ZX analyzed the MRI data. HW analyzed the transcription data. NZ analyzed the behavioral data. YZ wrote the initial draft. All authors agreed with the final version of the manuscript.

## Conflict of Interest

The authors declare that the research was conducted in the absence of any commercial or financial relationships that could be construed as a potential conflict of interest.

## Publisher’s Note

All claims expressed in this article are solely those of the authors and do not necessarily represent those of their affiliated organizations, or those of the publisher, the editors and the reviewers. Any product that may be evaluated in this article, or claim that may be made by its manufacturer, is not guaranteed or endorsed by the publisher.

## Footnotes

1
http://adni.loni.ucla.edu

2
http://surfer.nmr.mgh.harvard.edu/

3
http://www.fmrib.ox.ac.uk

4
http://human.brain-map.org/

5
https://doi.org/10.6084/m9.figshare.2057796.v1

6
https://www.ncbi.nlm.nih.gov/geo/

7
http://genetics.wustl.edu/jdlab/psi_package/
