# Supplementary material for: Bridging the Gap Between Morphometric Similarity Mapping and Gene Transcription in Alzheimer’s Disease
Source: Front Neurosci. 2021 Sep 29;15:731292. doi: 10.3389/fnins.2021.731292 (PMC8522649; doi:10.3389/fnins.2021.731292)
Supplement: Supplementary file 1 [file Data_Sheet_1.docx]

**Supplementary Material**

**1. Supplementary material and methods**

**1. 1 Surface-based morphology analysis**

Cortical reconstruction and surface-based morphology parameter estimation from the high-resolution T1-weighted images were performed using FreeSurfer v6.0.0 (http://surfer.nmr.mgh.harvard.edu/). The main processing steps included motion correction, affine transformation to MNI305 space, intensity normalization, skull stripping, tissue segmentation, and surface reconstruction. The individual surfaces were then inflated into a spherical space and nonlinearly registered to a spherical atlas from the FreeSurfer average subject (Fsaverage) template (Fischl et al., 1999) . The original 68 cortical regions in the Desikan-Killiany atlas were sub-parcellated into 308 (left 152, right 156) continuous cortical areas of 5mm^2^ using the backtracking algorithm, and this parcellation scheme was applied on the surface of the Fsaverage template (Romero-Garcia et al., 2012;Whitaker et al., 2016). Then, surface-based morphology parameters of the 308 cortical regions were extracted based on the newly defined Fsaverage template after registering it to individual spaces using the spherical registration parameters.

**1.2 Calculation of** **diffusion** **parameters**

The DTI data were preprocessed according to the pipeline of FMRIB's Diffusion Toolbox implemented in FSL 5.0.10 (http://www.fmrib.ox.ac.uk). The eddy current-induced distortion and motion artifacts were corrected using affine alignment of each diffusion weighted image to the b = 0 image. Then, skulls in the images were removed using the brain extract toolbox. The diffusion tensor was fitted using a linear least square algorithm and eigenvalues were decomposed from the tensor to calculate the fractional anisotropy and mean diffusion. The 308 cortical parcellation generated on the surface of Fsaverage template was interpolated and expanded to DTI volumes. Then, diffusion parameters of the 308 cortical regions were extracted based on the newly defined Fsaverage template after registering it to individual spaces using the registration information from DTI b = 0 image to individual T1 weighted image in MNI305 space.

**1.3 Transcription-imaging association**

A compiled transcription matrix of six post mortem adult brains from the AHBA (http://human.brain-map.org/) was acquired from the data directory for Neuroscience in Psychiatry Network manuscript (https://doi.org/10.6084/m9.figshare.2057796.v1). Two of the 308 cortical regions (lateraloccipital_part8 on the left hemisphere and parahippocampal_part1 on the right hemisphere) were excluded, as the mean and range of gene expression values in both regions were outliers compared with the other cortical regions. Since the AHBA only includes expression profile for the right hemisphere in two subjects, the gene expression profiles only from the left hemisphere (151 cortical regions) were used in our transcription-imaging association. Thus, the final transcription matrix includes expression values for each of 20,737 genes estimated in 151 cortical regions of the left hemisphere.

The PLS regression, a multivariate regression and data reduction method that could predict a set of dependent variables from a set of independent variables, was used to identify genes whose transcriptional profiles were significant associated with regional MS difference. In this study, the independent variables were the normalized matrix of AHBA gene expression profiles from the left hemisphere (151 regions × 20737 genes), and the dependent variables were the vector of regional MS case-control *T* values also from the left hemisphere (151 regions). The PLS regression was performed using full 150 components, and percentage variances in the case-control *T* statistics explained by top 15 components were plotted (**Supplemental Figure S1**). Among the top 15 components, the first PLS component (PLS1) was adopted because it explains the maximum variance (22.9%) in regional MS difference between AD and healthy elders. The PLS1 weight of each gene was assigned in terms of its contribution to the overall model. Then, the ratio of each gene's PLS1 weight to its bootstrapped standard error (1000 resampling with replacement of the 151 cortical regions) was calculated as a *Z* score. Here, genes with | *Z* score | > 4.72 (Bonferroni correction of *P* < 0.05) denote the PLS1 gene set.

**1.4 Disease enrichment analyses**

Disease enrichment analyses was used to explore whether the PLS1 gene set enriched in AD-related differentially expressed genes (DEGs), including both up and down-regulated DEGs. The expression dataset with series accession number GSE5281 from Gene Expression Omnibus database was acquired to screen the AD-related DEGs. The GSE5281 series based on the GPL570 platform of Affymetrix Human Genome U133 Plus 2.0 Array (HG-U133_Plus_2) provides the gene expression profile of brain samples from entorhinal cortex, hippocampus, medial temporal gyrus, posterior cingulate, superior frontal gyrus and primary visual cortex in 10 AD and 13 healthy elders(Liang et al., 2007;Liang et al., 2008a;Liang et al., 2008b;Readhead et al., 2018). The LIMMA package (version 3.42.2) of R software was used to analyses the DEGs between AD and normal elders(Ritchie et al., 2015). Specifically, a design matrix was firstly constructed according to the group assignment of AD and healthy elders. A linear model was fitted to the expression data for each gene. Then, empirical Bayes statistic was performed for differential expression between AD and normal elders. The *P* < 0.01 and | log2(fold change) | > 1 were defined as the thresholds for screening AD-related DESs. The Fisher's exact test was used to evaluate the significance of the overlap between PLS1 gene sets and AD-related DEGs using 20,177 genes from the GSE5281 series as the background gene list. The Bonferroni method was used to correct for multiple comparisons (both up and down-regulated DEGs) (*Pc* < 0.05, an uncorrected *P* < 0.05/2 =0.025). Genes that are significantly up-regulated or down-regulated were continued for further analyses.

**1.5** **Cell-type-specific analysis**

The RNAseq dataset with series accession number GSE73721 from Gene Expression Omnibus database (http://www.ncbi.nlm.gov/geo) was acquired to perform cell-type-specific analysis for PLS1- genes. The GSE73721 dataset provides the expression profiling of purified CNS cell types from both human and mouse by high throughput sequencing (https://www.ncbi.nlm.nih.gov/geo/query/acc.cgi?acc=GSE73721). Specifically, the samples of this dataset contain 6 normal fetal human astrocyte samples, 12 normal adult human astrocyte samples, 8 GBM or sclerotic human hippocampal samples, 4 whole normal human cortex samples, 4 adult mouse astrocyte samples, and 11 normal adult human samples of other purified CNS cell types (1 neurons, 5 oligodendrocytes, 3 microglia/myeloid cell and 1 endotheliocyte) in this dataset. For the purpose of our study, only the averaged expression levels of neurons, astrocytes, oligodendrocytes and microglia from normal adult human cortical samples were used (Zhang et al., 2016). The log2 normalized and averaged expression profile of each neocortical cell type were used in the specificity index (SI) analysis to determine the specific neocortical cell type for which the PLS1- genes were enriched using pSI v1.1 (http://genetics.wustl.edu/jdlab/psi_package/). Specifically, expression profile from one neocortical cell type were compared to those of the other cell types across genes. For each comparison between cell types, the genes were ranked from the highest to the lowest fold changes. The SI for each gene was calculated as the average rank across all comparisons. A *P* value was assigned to each SI value via permutation testing, resulting in a pSI value, representing how likely it was that a gene was specifically expressed in a given cell type relative to other cortical cells. A pSI threshold of 0.05 was used to generate the cell-type-enriched gene lists for each type of cortical cells. The total cell-type-enriched genes of all cortical cells were used as the background genes. The Fisher's exact test was used to evaluate the significance of the overlap between PLS1 gene sets and cell-type-specific genes for each type of cortical cells. The Bonferroni method was used to correct for multiple comparisons (5 cell-types) (*Pc* < 0.05, an uncorrected *P* < 0.05/4 =0.01).

**2. Supplementary results**

**2.1 Mean MS values before and after ComBat harmonization**

The 3D T1-weighted image and DTI of 212 subjects from ADNI database were acquired at 37 sites, where 34 sites equipped with one type of scanner for each site, 2 sites equipped with 2 types of scanners for each site, and 1 site equipped with 3 types of scanners **(Supplemental Table1)**. Therefore, a total of 45 different batches existed from multiple sites and scanners. The mean MS values across 308 brain regions were shown for each batch before and after ComBat harmonization **(Supplemental Figure S2)**. Though different scan protocols still affected the results, the variation from multiple sites and scanners could be moderately adjusted.

**2.2 Examples of 3 genes in PLS1- gene set downregulated in AD**

Adenylate kinase 5 encoded by *AK5* gene was found to be downregulated in entorhinal and frontal cortex of AD stages V-VI of Braak and Braa. Adenylate kinase 5 participates the purine metabolism, deregulation of which mainly affects energy metabolism and cell signaling either between neurons or neurons and glial cells in AD (Ansoleaga et al., 2015).

*TUBB* gene encodes a beta tubulin protein which normally associate into heterodimers to serve as the building blocks of microtubules. Decreased expression of beta tubulin proteins was identified across entorhinal cortex, hippocampus, middle temporal gyrus, and posterior cingulate cortex in AD, dissociation of tau from which indicates aberrant hyperphosphorylation of tau, neurofibrillary tangle formation, and eventually neuron death (Liang et al., 2008a).

*ATP6V0C* gene encodes ATP6V0C, a multi-functional protein that appears to function at the intersection of a number of biological processes. In addition to its role in V-ATPase, ATP6V0C is also a kind of synaptic protein and plays an important role in neurotransmitter release, the degradation of which mediated by another protein, RNF182, may contribute to neurodegeneration in AD (Liu et al., 2008).

**Reference**

Ansoleaga, B., Jove, M., Schluter, A., Garcia-Esparcia, P., Moreno, J., Pujol, A., Pamplona, R., Portero-Otin, M., and Ferrer, I. (2015). Deregulation of purine metabolism in Alzheimer's disease. *Neurobiol Aging* 36**,** 68-80.

Fischl, B., Sereno, M.I., Tootell, R.B., and Dale, A.M. (1999). High-resolution intersubject averaging and a coordinate system for the cortical surface. *Hum Brain Mapp* 8**,** 272-284.

Liang, W.S., Dunckley, T., Beach, T.G., Grover, A., Mastroeni, D., Ramsey, K., Caselli, R.J., Kukull, W.A., Mckeel, D., Morris, J.C., Hulette, C.M., Schmechel, D., Reiman, E.M., Rogers, J., and Stephan, D.A. (2008a). Altered neuronal gene expression in brain regions differentially affected by Alzheimer's disease: a reference data set. *Physiol Genomics* 33**,** 240-256.

Liang, W.S., Dunckley, T., Beach, T.G., Grover, A., Mastroeni, D., Walker, D.G., Caselli, R.J., Kukull, W.A., Mckeel, D., Morris, J.C., Hulette, C., Schmechel, D., Alexander, G.E., Reiman, E.M., Rogers, J., and Stephan, D.A. (2007). Gene expression profiles in anatomically and functionally distinct regions of the normal aged human brain. *Physiol Genomics* 28**,** 311-322.

Liang, W.S., Reiman, E.M., Valla, J., Dunckley, T., Beach, T.G., Grover, A., Niedzielko, T.L., Schneider, L.E., Mastroeni, D., Caselli, R., Kukull, W., Morris, J.C., Hulette, C.M., Schmechel, D., Rogers, J., and Stephan, D.A. (2008b). Alzheimer's disease is associated with reduced expression of energy metabolism genes in posterior cingulate neurons. *Proc Natl Acad Sci U S A* 105**,** 4441-4446.

Liu, Q.Y., Lei, J.X., Sikorska, M., and Liu, R. (2008). A novel brain-enriched E3 ubiquitin ligase RNF182 is up regulated in the brains of Alzheimer's patients and targets ATP6V0C for degradation. *Mol Neurodegener* 3**,** 4.

Readhead, B., Haure-Mirande, J.V., Funk, C.C., Richards, M.A., Shannon, P., Haroutunian, V., Sano, M., Liang, W.S., Beckmann, N.D., Price, N.D., Reiman, E.M., Schadt, E.E., Ehrlich, M.E., Gandy, S., and Dudley, J.T. (2018). Multiscale Analysis of Independent Alzheimer's Cohorts Finds Disruption of Molecular, Genetic, and Clinical Networks by Human Herpesvirus. *Neuron* 99**,** 64-82.e67.

Ritchie, M.E., Phipson, B., Wu, D., Hu, Y., Law, C.W., Shi, W., and Smyth, G.K. (2015). limma powers differential expression analyses for RNA-sequencing and microarray studies. *Nucleic Acids Res* 43**,** e47.

Romero-Garcia, R., Atienza, M., Clemmensen, L.H., and Cantero, J.L. (2012). Effects of network resolution on topological properties of human neocortex. *Neuroimage* 59**,** 3522-3532.

Whitaker, K.J., Vertes, P.E., Romero-Garcia, R., Vasa, F., Moutoussis, M., Prabhu, G., Weiskopf, N., Callaghan, M.F., Wagstyl, K., Rittman, T., Tait, R., Ooi, C., Suckling, J., Inkster, B., Fonagy, P., Dolan, R.J., Jones, P.B., Goodyer, I.M., and Bullmore, E.T. (2016). Adolescence is associated with genomically patterned consolidation of the hubs of the human brain connectome. *Proc Natl Acad Sci U S A* 113**,** 9105-9110.

Zhang, Y., Sloan, S.A., Clarke, L.E., Caneda, C., Plaza, C.A., Blumenthal, P.D., Vogel, H., Steinberg, G.K., Edwards, M.S., Li, G., Duncan, J.A., 3rd, Cheshier, S.H., Shuer, L.M., Chang, E.F., Grant, G.A., Gephart, M.G., and Barres, B.A. (2016). Purification and Characterization of Progenitor and Mature Human Astrocytes Reveals Transcriptional and Functional Differences with Mouse. *Neuron* 89**,** 37-53.


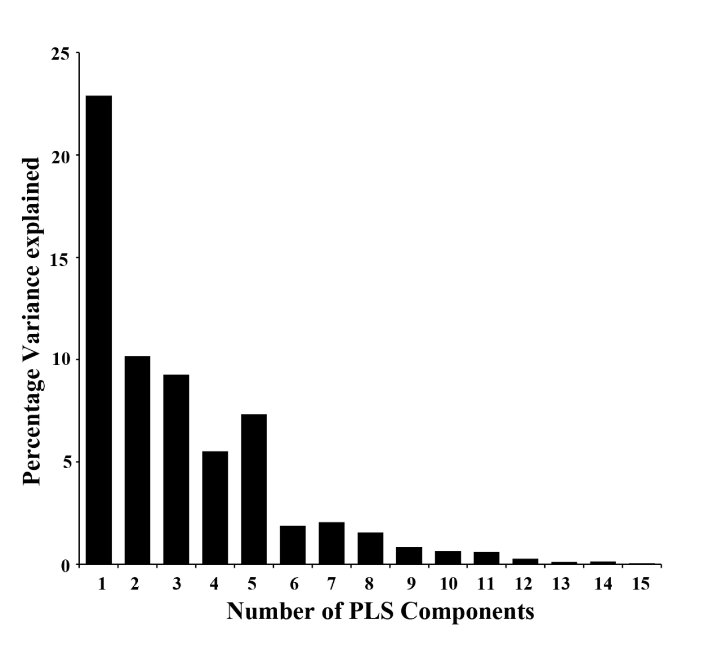


**Figure S1** Percentage variance explained by the top 15 components. The first PLS component (PLS1) explains the maximum proportion of variance (22.9%) in regional morphometric similarity difference between AD and normal elders and is selected for further analyses. PL partial least squares regression.


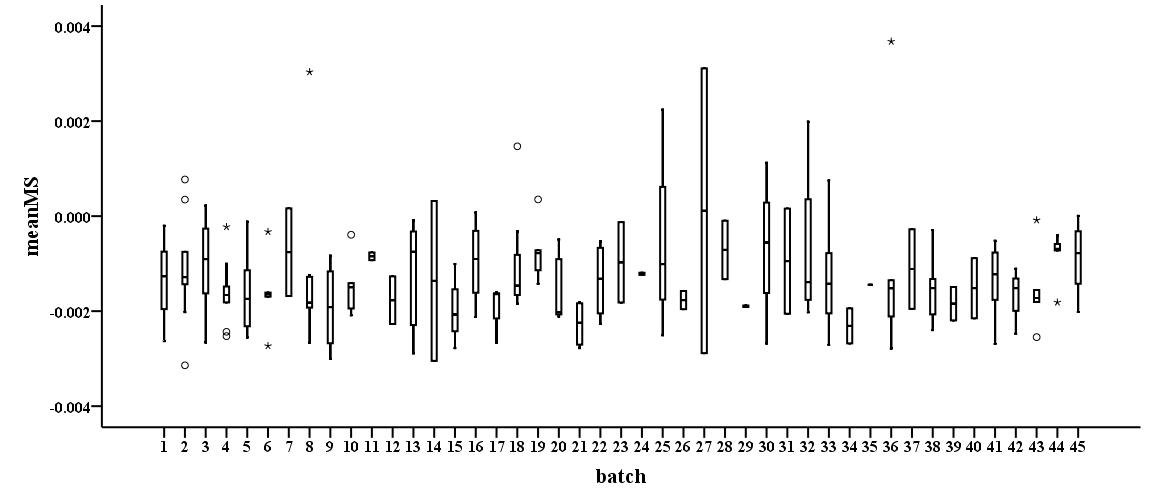

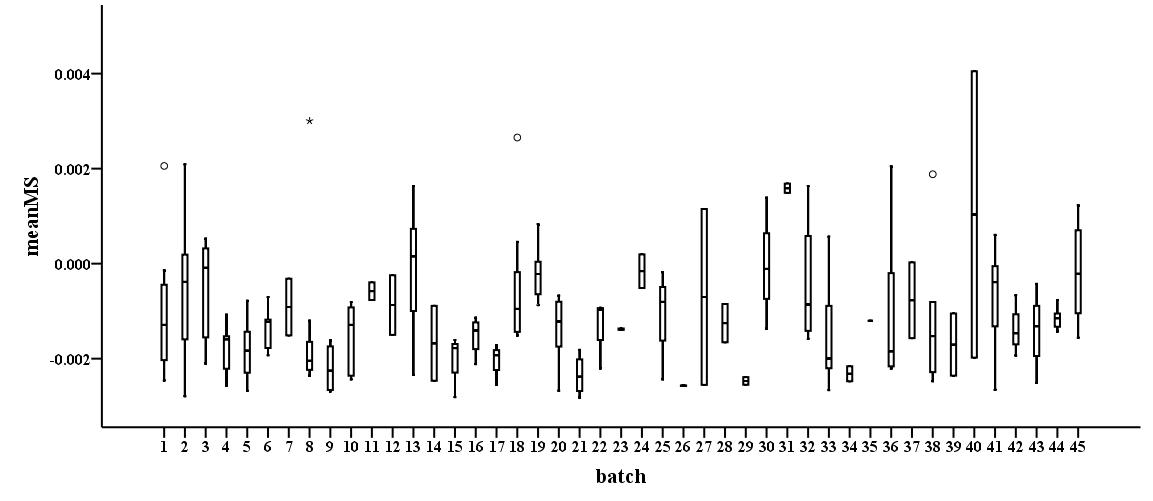


**Figure S2** The mean MS values across 308 brain regions for the 45 batches before (upper) and after (lower) ComBat harmonization. The variations of mean MS values are moderately controlled after ComBat harmonization. MS, morphometric similarity.
