# Supplementary material for: Bridging the Gap Between Morphometric Similarity Mapping and Gene Transcription in Alzheimer’s Disease
Source: Front Neurosci. 2021 Sep 29;15:731292. doi: 10.3389/fnins.2021.731292 (PMC8522649; doi:10.3389/fnins.2021.731292)
Supplement: Supplementary file 3 [file Table_1.DOCX]

**Table S1** Scan parameters of participates from ADNI database

| **T1WI scan parameters** | | | | | | | | | | | | | | |
| --- | --- | --- | --- | --- | --- | --- | --- | --- | --- | --- | --- | --- | --- | --- |
| **Sites**  **ID** | **Manufacturing**  **Models** | **Field Strength**  **(Tesla)** | **Acquisition**  **Type** | **Flip Angle**  **(degree)** | **Matrix**  **(x,y,z)（pixels）** | | | **Pixel Spacing (x,y) (mm)** | | **Pulse Sequence** | **Slice**  **Thickness**  **(mm)** | **TE**  **(ms)** | **TI**  **（ms）** | **TR**  **(ms)** |
| 002 | SIEMENS Prisma_fit | 3.0 | 3D | 9 | 240 | 256 | 176/208 | 1.0/1.1 | 1.0/1.1 | GR/IR | 1.0/1.2 | 3.0 | 900.0 | 2300.0 |
| 003 | GE  Signa HDxt | 3.0 | 3D | 11 | 256 | 256 | 196 | 1.0/1.1 | 1.0/1.1 | GR | 1.2 | 2.8/3.0 | 400.0 | 7.0/7.2 |
| 003 | SIEMENS Prisma | 3.0 | 3D | 9 | 240 | 256 | 208 | 1.0 | 1.0 | GR/IR | 1.0 | 3.0 | 900.0 | 2300.0 |
| 005 | GE DISCOVERY MR750 | 3.0 | 3D | 11 | 256 | 256 | 196 | 1.0 | 1.0 | GR | 1.0/1.2 | 3.0/3.1 | 400.0 | 7.3/7.4 |
| 006 | Philips Ingenia | 3.0 | 3D | 9 | 256 | 256 | 211/422 | 1.0 | 1.0 | GR | 1.0 | 2.9 | 0.0 | 6.5 |
| 007 | GE Signa HDxt | 3.0 | 3D | 11 | 256 | 256 | 196 | 1.0/1.1 | 1.0/1.1 | GR | 1.2 | 2.8 | 400.0 | 7.0 |
| 007 | SIEMENS Prisma | 3.0 | 3D | 9 | 240 | 256 | 208 | 1.0 | 1.0 | GR/IR | 1.0 | 3.0 | 900.0 | 2300.0 |
| 009 | GE DISCOVERY MR750w | 3.0 | 3D | 11 | 256 | 256 | 196 | 1.0 | 1.0 | GR | 1.0 | 3.1 | 400.0 | 7.7 |
| 011 | SIEMENS Prisma_fit | 3.0 | 3D | 9 | 240 | 256 | 208 | 1.0 | 1.0 | GR/IR | 1.0 | 3.0 | 900.0 | 2300.0 |
| 013 | SIEMENS Biograph_mMR | 3.0 | 3D | 9 | 240 | 256 | 176 | 1.0 | 1.0 | GR/IR | 1.0 | 3.0 | 900.0 | 2300.0 |
| 014 | SIEMENS Verio | 3.0 | 3D | 9 | 240 | 256 | 176 | 1.0 | 1.0 | GR/IR | 1.0 | 3.0 | 900.0 | 2300.0 |
| 016 | GE Signa HDxt | 3.0 | 3D | 11 | 256 | 256 | 196 | 1.0/1.1 | 1.0/1.1 | GR | 1.2 | 2.8 | 400.0 | 7.0 |
| 016 | SIEMENS Skyra | 3.0 | 3D | 9 | 240 | 256 | 176 | 1.1 | 1.1 | GR/IR | 1.2 | 3.0 | 900.0 | 2300.0 |
| 021 | GE DISCOVERY MR750 | 3.0 | 3D | 11 | 256 | 256 | 196 | 1.1 | 1.1 | GR | 1.2 | 3.0 | 400.0 | 7.3 |
| 022 | SIEMENS TrioTim | 3.0 | 3D | 9 | 240 | 256 | 176 | 1.0/1.1 | 1.0/1.1 | GR/IR | 1.0/1.2 | 3.0 | 900.0 | 2300.0 |
| 022 | SIEMENS Prisma_fit | 3.0 | 3D | 9 | 240 | 256 | 208 | 1.0 | 1.0 | GR/IR | 1.0 | 3.0 | 900.0 | 2300.0 |
| 023 | SIEMENS Prisma_fit | 3.0 | 3D | 9 | 240 | 256 | 208 | 1.0 | 1.0 | GR/IR | 1.0 | 3.0 | 900.0 | 2300.0 |
| 027 | GE Signa HDxt | 3.0 | 3D | 11 | 256 | 256 | 196 | 1.1 | 1.1 | GR | 1.2 | 2.8/3.0 | 400.0 | 7.0/7.2 |
| 027 | GE DISCOVERY MR750w | 3.0 | 3D | 11 | 256 | 256 | 196 | 1.1 | 1.1 | GR | 1.2 | 3.1 | 400.0 | 7.6 |
| 027 | GE DISCOVERY MR750 | 3.0 | 3D | 11 | 256 | 256 | 196 | 1.0 | 1.0 | GR | 1.0 | 3.1 | 400.0 | 7.4 |
| 029 | GE Signa HDxt | 3.0 | 3D | 11 | 256 | 256 | 196 | 1.0 | 1.0 | GR | 1.2 | 2.8 | 400.0 | 7.0 |
| 032 | SIEMENS Prisma_fit | 3.0 | 3D | 9 | 240 | 256 | 208 | 1.0 | 1.0 | GR/IR | 1.0 | 3.0 | 900.0 | 2300.0 |
| 033 | SIEMENS Skyra | 3.0 | 3D | 9 | 240 | 256 | 176 | 1.0 | 1.0 | GR/IR | 1.0 | 3.0 | 900.0 | 2300.0 |
| 035 | SIEMENS Prisma_fit | 3.0 | 3D | 9 | 240 | 256 | 208 | 1.0 | 1.0 | GR/IR | 1.0 | 3.0 | 900.0 | 2300.0 |
| 036 | SIEMENS Skyra | 3.0 | 3D | 9 | 240 | 256 | 176 | 1.1 | 1.1 | GR/IR | 1.2 | 3.0 | 900.0 | 2300.0 |
| 037 | SIEMENS Prisma | 3.0 | 3D | 9 | 240 | 256 | 208 | 1.0 | 1.0 | GR/IR | 1.0 | 3.0 | 900.0 | 2300.0 |
| 041 | SIEMENS Prisma_fit | 3.0 | 3D | 9 | 240 | 256 | 176 | 1.1 | 1.1 | GR/IR | 1.2 | 3.0 | 900.0 | 2300.0 |
| 052 | GE DISCOVERY MR750 | 3.0 | 3D | 11 | 256 | 256 | 196 | 1.1 | 1.1 | GR | 1.2 | 3.0 | 400.0 | 7.3 |
| 082 | SIEMENS Verio | 3.0 | 3D | 9 | 240 | 256 | 176 | 1.0 | 1.0 | GR/IR | 1.0 | 3.0 | 900.0 | 2300.0 |
| 094 | GE Signa HDxt | 3.0 | 3D | 11 | 256 | 256 | 196 | 1.0 | 1.0 | GR | 1.2 | 2.8 | 400.0 | 7.0 |
| 094 | SIEMENS Prisma | 3.0 | 3D | 9 | 240 | 256 | 176 | 1.1 | 1.1 | GR/IR | 1.2 | 3.0 | 900.0 | 2300.0 |
| 098 | GE DISCOVERY MR750 | 3.0 | 3D | 11 | 256 | 256 | 196 | 1.0 | 1.0 | GR | 1.2 | 3.0 | 400.0 | 7.3 |
| 098 | GE SIGNA Premier | 3.0 | 3D | 11 | 256 | 256 | 196 | 1.0 | 1.0 | GR/IR | 1.0 | 2.9 | 400.0 | 7.2 |
| 109 | GE Signa HDxt | 3.0 | 3D | 11 | 256 | 256 | 196 | 1.0/1.1 | 1.0/1.1 | GR | 1.2 | 2.8 | 400.0 | 7.0 |
| 114 | SIEMENS Verio | 3.0 | 3D | 9 | 240 | 256 | 176 | 1.0 | 1.0 | GR/IR | 1.0 | 3.0 | 900.0 | 2300.0 |
| 116 | SIEMENS TrioTim | 3.0 | 3D | 9 | 240 | 256 | 176 | 1.0 | 1.0 | GR/IR | 1.0 | 3.0 | 900.0 | 2300.0 |
| 126 | GE DISCOVERY MR750 | 3.0 | 3D | 11 | 256 | 256 | 196 | 1.0 | 1.0 | GR | 1.0/1.2 | 3.0/3.1 | 400.0 | 7.3/7.4 |
| 127 | GE DISCOVERY MR750 | 3.0 | 3D | 11 | 256 | 256 | 196 | 1.1 | 1.1 | GR | 1.2 | 3.0 | 400.0 | 7.3 |
| 129 | GE DISCOVERY MR750 | 3.0 | 3D | 11 | 256 | 256 | 196 | 1.0 | 1.0 | GR | 1.0 | 3.1 | 400.0 | 7.4 |
| 130 | Philips Achieva dStream | 3.0 | 3D | 9 | 256 | 256 | 211 | 1.0 | 1.0 | GR | 1.0 | 2.9 | 0.0 | 6.5 |
| 135 | GE DISCOVERY MR750w | 3.0 | 3D | 11 | 256 | 256 | 196 | 1.0 | 1.0 | GR | 1.0 | 3.1 | 400.0 | 7.7 |
| 137 | SIEMENS Skyra | 3.0 | 3D | 9 | 240 | 256 | 176/208 | 1.0/1.1 | 1.0/1.1 | GR/IR | 1.0/1.2 | 3.0 | 900.0 | 2300.0 |
| 168 | SIEMENS Prisma_fit | 3.0 | 3D | 9 | 240 | 256 | 208 | 1.0 | 1.0 | GR/IR | 1.0 | 3.0 | 900.0 | 2300.0 |
| 301 | Philips Ingenia | 3.0 | 3D | 9 | 256 | 256 | 211 | 1.0 | 1.0 | GR | 1.0 | 2.9 | 0.0 | 6.5 |
| 341 | SIEMENS Prisma | 3.0 | 3D | 9 | 240 | 256 | 208 | 1.0 | 1.0 | GR/IR | 1.0 | 3.0 | 900.0 | 2300.0 |

Note: GR, gradient recalled sequence; IR, inversion recovery pulse sequence.

| **DIT scan parameters** | | | | | | | | | | | | | | |
| --- | --- | --- | --- | --- | --- | --- | --- | --- | --- | --- | --- | --- | --- | --- |
| **Sites**  **ID** | **Manufacturing**  **Models** | **Field Strength**  **(Tesla)** | **Acquisition**  **Type** | **Flip Angle**  **(degree)** | **Gradient**  **Directions** | **Matrix**  **(x,y,z)（pixels）** | | | **Pixel Spacing (x,y) (mm)** | | **Pulse Sequence** | **Slice**  **Thickness**  **(mm)** | **TE**  **(ms)** | **TR**  **（ms）** |
| 002 | SIEMENS Prisma_fit | 3.0 | 2D | 90 | 54 | 1044 | 1044 | 55 | 2.0/2.5 | 2.0/2.5 | EP | 2.0 | 56.0 | 7200.0 |
| 003 | GE Signa HDxt | 3.0 | 2D | 90 | 41 | 256 | 256 | 2208/2714 | 1.4 | 1.4 | EP/SE | 2.7 | 68.3/68.4/68.5/68.7/69.3 | 10000.0/  13000.0 |
| 003 | SIEMENS Prisma | 3.0 | 2D | 90 | 54 | 1044 | 1044 | 55 | 2.0 | 2.0 | EP | 2.0 | 56.0 | 7200.0 |
| 005 | GE DISCOVERY MR750 | 3.0 | 2D | 90 | 41/48 | 256 | 256 | 2714/4320 | 0.9/1.4 | 0.9/1.4 | EP/SE | 2.0/2.7 | 63.0/60.9 | 7800.0  /9050.0 |
| 006 | Philips Ingenia | 3.0 | 2D | 90 | 0 | 128 | 128 | 2880/5760 | 2.0 | 2.0 | SE | 2.0 | 99.6 | 10948.0  /10948.1 |
| 007 | GE Signa HDxt | 3.0 | 2D | 90 | 41 | 256 | 256 | 2668/2714 | 1.4 | 1.4 | EP/SE | 2.7 | 68.3/68.4/68.5 | 12300.0 |
| 007 | SIEMENS Prisma | 3.0 | 2D | 90 | 126 | 1044 | 1044 | 127 | 2.0 | 2.0 | EP | 2.0 | 71.0 | 3400.0 |
| 009 | GE DISCOVERY MR750w | 3.0 | 2D | 90 | 32 | 256 | 256 | 2880 | 0.9 | 0.9 | EP/SE | 2.0 | 74.4/74.6/74.9 | 15261.0  /15354.0  /15421.0 |
| 011 | SIEMENS Prisma_fit | 3.0 | 2D | 90 | 54 | 1044 | 1044 | 55 | 2.0 | 2.0 | EP | 2.0 | 56.0 | 7200.0 |
| 013 | SIEMENS Biograph_mMR | 3.0 | 2D | 90 | 30 | 1044 | 1044 | 31 | 2.0 | 2.0 | EP | 2.0 | 95.0 | 12400.0 |
| 014 | SIEMENS Verio | 3.0 | 2D | 90 | 30 | 1044 | 1044 | 31 | 2.0 | 2.0 | EP | 2.0 | 95.0 | 12400.0 |
| 016 | GE Signa HDxt | 3.0 | 2D | 90 | 41 | 256 | 256 | 2714/2760 | 1.4 | 1.4 | EP/SE | 2.7 | 68.3/68.4/68.6 | 12500.0 |
| 016 | SIEMENS Skyra | 3.0 | 2D | 90 | 54 | 1160/1044 | 1160/1044 | 55 | 2.0 | 2.0 | EP | 2.0 | 82.0 | 9800.0  /9600.0 |
| 021 | GE DISCOVERY MR750 | 3.0 | 2D | 90 | 41/48 | 256 | 256 | 2714/4320 | 0.9/1.4 | 0.9/1.4 | EP/SE | 2.0/2.7 | 60.6/63.0/62.8 | 7800.0/  9050.0 |
| 022 | SIEMENS TrioTim | 3.0 | 2D | 90 | 30 | 1044 | 1044 | 31 | 2.0 | 2.0 | EP | 2.0 | 95.0 | 12400.0 |
| 022 | SIEMENS Prisma_fit | 3.0 | 2D | 90 | 126 | 1044 | 1044 | 127 | 2.0 | 2.0 | EP | 2.0 | 71.0 | 3400.0 |
| 023 | SIEMENS Prisma_fit | 3.0 | 2D | 90 | 126 | 1044 | 1044 | 127/44 | 2.0 | 2.0 | EP | 2.0 | 71.0 | 3400.0 |
| 027 | GE Signa HDxt | 3.0 | 2D | 90 | 41 | 256 | 256 | 2806/2760 | 1.4 | 1.4 | EP/SE | 2.7 | 69.3/68.3 | 12700.0 |
| 027 | GE DISCOVERY MR750w | 3.0 | 2D | 90 | 41 | 256 | 256 | 2438/2576 | 1.4 | 1.4 | EP/SE | 2.7 | 94.4/92.6 | 12700.0 |
| 027 | GE DISCOVERY MR750 | 3.0 | 2D | 90 | 48 | 256 | 256 | 4320 | 0.9 | 0.9 | EP/SE | 2.0 | 60.4/60.5/60.6/60.7 | 7800.0 |
| 029 | GE Signa HDxt | 3.0 | 2D | 90 | 41 | 256 | 256 | 2714 | 1.4 | 1.4 | EP/SE | 2.7 | 68.4 | 13000.0 |
| 032 | SIEMENS Prisma_fit | 3.0 | 2D | 90 | 54 | 1044 | 1044 | 41/55 | 2.0 | 2.0 | EP | 2.0 | 56.0 | 9600.0 |
| 033 | SIEMENS Skyra | 3.0 | 2D | 90 | 54 | 1044 | 1044 | 55 | 2.0 | 2.0 | EP | 2.0 | 82.0 | 9600.0 |
| 035 | SIEMENS Prisma_fit | 3.0 | 2D | 90 | 54 | 1044 | 1044 | 55 | 2.0 | 2.0 | EP | 2.0 | 56.0 | 7200.0 |
| 036 | SIEMENS Skyra | 3.0 | 2D | 90 | 54/126 | 1044 | 1044 | 55/127 | 2.0/2.2 | 2.0/2.2 | EP | 2.0 | 82.0/99.0 | 4200.0/  9600.0 |
| 037 | SIEMENS Prisma | 3.0 | 2D | 90 | 54/126 | 1044 | 1044 | 55/127 | 2.0 | 2.0 | EP | 2.0 | 56.0/71.0 | 3400.0/  7200.0 |
| 041 | SIEMENS Prisma_fit | 3.0 | 2D | 90 | 54 | 1044 | 1044 | 55 | 2.0 | 2.0 | EP | 2.0 | 56.0 | 7200.0 |
| 052 | GE DISCOVERY MR750 | 3.0 | 2D | 90 | 41 | 256 | 256 | 2714 | 1.4 | 1.4 | EP/SE | 2.7 | 61.9 | 9050.0 |
| 082 | SIEMENS Verio | 3.0 | 2D | 90 | 30 | 1044 | 1044 | 31 | 2.0 | 2.0 | EP | 2.0 | 95.0 | 12500.0 |
| 094 | GE Signa HDxt | 3.0 | 2D | 90 | 41 | 256 | 256 | 2714 | 1.4 | 1.4 | EP/SE | 2.7 | 68.3 | 12500.0 |
| 094 | SIEMENS Prisma | 3.0 | 2D | 90 | 54 | 1044/1160 | 1044/1160 | 55 | 2.0 | 2.0 | EP | 2.0 | 56.0 | 7200.0/  7600.0 |
| 098 | GE DISCOVERY MR750 | 3.0 | 2D | 90 | 41 | 256 | 256 | 2714 | 1.4 | 1.4 | EP/SE | 2.7 | 61.8 | 9050.0 |
| 098 | GE SIGNA Premier | 3.0 | 2D | 90 | 48 | 256 | 256 | 3726/3780 | 0.9 | 0.9 | EP/SE | 2.0 | 54.7/54.9 | 7800.0 |
| 109 | GE Signa HDxt | 3.0 | 2D | 90 | 41 | 256 | 256 | 2714 | 1.4 | 1.4 | EP/SE | 2.7 | 82.3/83.5 | 14200.0/  14300.0 |
| 114 | SIEMENS Verio | 3.0 | 2D | 90 | 30 | 1152 | 1152 | 31 | 2.7 | 2.7 | EP | 2.0 | 105.0 | 16700.0 |
| 116 | SIEMENS TrioTim | 3.0 | 2D | 90 | 30 | 1044 | 1044 | 31 | 2.0 | 2.0 | EP | 2.0 | 95.0 | 12400.0 |
| 126 | GE DISCOVERY MR750 | 3.0 | 2D | 90 | 41/48 | 256 | 256 | 2714/4320 | 0.9/1.4 | 0.9/1.4 | EP/SE | 2.0/2.7 | 60.8/  61.6/  61.9/  62.0 | 7800.0/  7850.0/  9050.0 |
| 127 | GE DISCOVERY MR750 | 3.0 | 2D | 90 | 41/48 | 256 | 256 | 2714/2760/4320 | 0.9/1.4 | 0.9/1.4 | EP/SE | 2.0/2.7 | 60.6/  60.7/  61.8/  61.9/  62.0 | 7800.0/  9050.0 |
| 129 | GE DISCOVERY MR750 | 3.0 | 2D | 90 | 48 | 256 | 256 | 1760/4320 | 0.9 | 0.9 | EP/SE | 2.0 | 60.4/  60.6 | 7800.0 |
| 130 | Philips Achieva dStream | 3.0 | 2D | 90 | 0 | 128 | 128 | 1 | 2.0 | 2.0 | SE | 2.0 | 87.8 | 10011.0 |
| 135 | GE DISCOVERY MR750w | 3.0 | 2D | 90 | 32 | 256 | 256 | 2880 | 0.9 | 0.9 | EP/SE | 2.0 | 74.7/  74.8/  74.9 | 15964.0/  15993.0/  16024.0/  16033.0 |
| 137 | SIEMENS Skyra | 3.0 | 2D | 90 | 54 | 1044 | 1044 | 55 | 2.0 | 2.0 | EP | 2.0 | 82.0 | 9600.0 |
| 168 | SIEMENS Prisma_fit | 3.0 | 2D | 90 | 54 | 1044 | 1044 | 55 | 2.0 | 2.0 | EP | 2.0 | 56.0 | 7200.0 |
| 301 | Philips Ingenia | 3.0 | 2D | 90 | 0 | 128 | 128 | 2880 | 2.0 | 2.0 | SE | 2.0 | 101.1 | 11199.0 |
| 341 | SIEMENS Prisma | 3.0 | 2D | 90 | 126 | 1044 | 1044 | 127 | 2.0 | 2.0 | EP | 2.0 | 71.0 | 3400.0 |

Note: EP, echo planar sequence; SE, spin echo sequence.
