# Supplementary material for: Bridging the Gap Between Morphometric Similarity Mapping and Gene Transcription in Alzheimer’s Disease
Source: Front Neurosci. 2021 Sep 29;15:731292. doi: 10.3389/fnins.2021.731292 (PMC8522649; doi:10.3389/fnins.2021.731292)
Supplement: Supplementary file 4 [file Table_2.DOCX]

**Table S2** PLS1 positive and negative gene sets

| **PLS1 negative genes** | | | | | |
| --- | --- | --- | --- | --- | --- |
| PACSIN1  RXRA  PDLIM3  CREG1  ATPIF1  MYO1F  DAB1  HSD17B14  EID2B  C4A  CLSPN  POLR3GL  TPO  FGFR3  SPINK9  TMEM45A  CRHBP  SPIN2B  PCDHGC3  RP5-820A21.1  WISP1  CPAMD8  APOBEC3G  FKBP10  LONRF2  LCP1  NOL9  PRSS3  PDYN  DYRK3  TRO  INPP4A  RALA  ATP6V0C  RP11-193H5.1  TCAP  ASL  CYBB  CPE  CSRP2BP  PPAPR3  LOC780529  TRDN  NEURL1B  ELK1  LOC100287987  MS4A4A  GLI1  TMEM242  NMT1  GNG8  CD63  PRRG3  RP11-561O23.6  ESD  SEC24D  B4GALT3  RTN1  SSBP4  GSG1  CD200  LRRN2  MFSD10  NXPH1  TBCB  GLUD1  1-Mar  DYRK2  HADH  ZDHHC24  LRRC27  C6orf108  TYMP  MIIP  CLYBL  TG  DNALI1  AC135592.2  FADS2  TMED9  KRT1  DERL3  SLC26A8  GPR173  ISG15  RTN4RL1  CACNG1  PTS  LOC144486  ATP8A2  AC004381.2  PSMB7  POLR2G  ADCY7  SLC7A11  SH3BGRL3  EIF1AY  WDR62  EBP  AC016747.3  RAB34  SLC1A6  EMP3  TSPAN6  GDAP1L1  AQP3  BRD7  SIDT1  CCS  CKS1B  GAB3  CSF1R  PET100  MT1B  GUK1  B3GNT1  UBQLNL  REC8  C1orf61  FAM116B  TMEM54  PEG10  DUSP5P  GRHL1  PPOX  FLRT1  C5  TP53I11  SNTA1  TLL1  CPSF4  IQCA1  SLC9A6  CYP4X1  C2orf60  PRRT1  C6orf118  GSTM2  CALB1  ORC6L  AP2S1  ST6GALNAC6  ADAMTS8  FAM131A  TOMM6  GTF3C6  KIAA1239  NDST2  SLC29A3  NLRC4  MILR1  CXADRP3  KIAA2022  SYTL1  LSP1  C21orf62  CDH8  WNT7B  PON3  4-Mar  C1orf173  PPP1R2  PTPRO  MEST  DDOST  BZRAP1  OXTR  TBC1D14  NTNG2  FOLR2  DCDC2  PLD3  TRIML2  MRPL28  RNF2  FAM181A  PROC  LRCH2  MYOZ1  NR0B1  OSR1  FBXO2  JPH4  CCDC90A  PRR19  C10orf35  HES4  GFPT1  RAB3GAP1  TRIM24  COPS4  C5orf55  WRB  DNAJC12  UPP2  ST5  CPT1C  C22orf23  HMGCLL1  GEMIN8P4  GP1BB  LOC644662  TMEM181  HMGCR  GABRA2  PITPNM2  S100Z  GPR56  TEK  RNASET2  P4HA2  AC073346.1  LOC389458  GTF2F2  KCNQ2  GCNT1  LYZL4  CD36  MS4A8B  HEBP1  KAL1  ENOX2  TSPAN4  SOX11  GRP  AF205589.2  DPF1  MNF1  YWHAB  PTPRR  CD244  C10orf107  WDR52  SLC25A22  MAFG  MBLAC2  FUCA1  USP9X  SFTPD  OCA2  SCUBE2  RP11-566K11.2  RNF181  IDS  GPR88  FZD1  SMAD3  AKAP14  ALG1L  KCNK13  TBC1D24  GPD2  AP3B2  ELL3  CALM3  NUPR1  DYX1C1-CCPG1  HIST1H2BK  RAC2  NLE1  CCDC103  PLD6  PGAP1  SH3RF1  AC009365.1  DLG2  NUDT17  LOC728392  NKAIN3  LRRC50  AP3M2  LPHN3  HMGCS1  LOC283143  FAH  TMEM52  HSPB11  RIMBP2  RDBP  MCFD2  C9orf9  PTBP2  GOLT1A  PDK2  CMTM8  CNIH2  RTBDN  LOC100289600  DTNBP1  TMEM130  SSSCA1  NECAB2  TRAPPC2L  TNNT1  KLK10  UBE2S  ANXA8  C3orf26  DCAKD  CEBPA  OTUB2  PTCHD1  PGRMC1  GPRIN2  TTLL1  RRAGB  BAIAP3  STK25  PLAC9  LINC00086  RIPK2  VAV1  AK7  AC024575.2  GRIA1  ZNF831  LINC00087  RSPO3  TDRD5  ASCL2  FAM150B  GDPD2  TXLNB  BPHL  TRIM6  KCNG1  TPM3  CCDC109B  LOC643037  KCTD12  LRRC42  SLC30A10  IL34  PCDH20 | RUNDC3A  LOC729722  DIS3L  TMEM107  RNASEK  UBE3A  GPR83  LOC150519  DERL1  LIX1  TPH2  SEMA6B  TAC1  ALPL  TPST1  CNTNAP3  NECAP1  HEXA  TNFRSF13C  CLTCL1  OVCH1  VANGL2  CYB5R1  STX12  LHX2  OTOS  L1CAM  RNF5P1  RPS10  SFRP2  SYNPO  TWIST2  NKD2  INTS4  CTGF  SHISA5  ATF7IP2  3-Mar  BRK1  NID1  BCL2L10  ACOT8  TNIP3  NEUROD2  LYPD6B  NPTX2  BYSL  KCNA4  SIGLEC11  EPB41L4B  SOX4  NSUN6  CENPE  LOC100289922  FRMPD2  RASAL3  RAB11FIP1  ZBTB44  PSMD1  ATP8B4  C8orf46  L3MBTL  LCMT1  FUK  MOBKL2A  PDCD2L  C1orf194  C12orf42  KCNJ1  SNAP47  RARRES3  RAE1  ELMO2  AKR7A2  PALLD  TMEM86A  AQP4  IHH  TFEC  MT1G  FGGY  ZDHHC15  C10orf46  PPP1R1A  ATBF1  SMPDL3A  ZNF642  PRPS1  B4GALT5  TRPV2  ALDH1A2  CDC42EP4  LOC643750  GMIP  KLRG1  OAF  GNG12  APAF1  SSR4  WBP1  RNF24  SHF  PARK2  ENC1  LOC728716  RPL27A  ONECUT1  DNAJA1  SLC17A8  OPRK1  MLEC  FBXW9  SLC2A10  NSUN7  ARL16  OR6C2  AP000880.1  ACTR3B  P2RY12  VKORC1L1  C1orf93  RPL12  OBSCN  GSG1L  GUCA1A  AP1M1  ZNF673  CSPG4  RP13-102H20.1  AL161668.2  NR2E1  PSMD9  ADI1  SMARCA1  THOC4  FAM151B  COQ7  FDXR  ARRB1  TAF10  CCDC42B  ADAM28  GPM6A  SLC2A4  LOC100128760  NPPA  CXCR7  HNRNPC  CNN3  GOSR2  DNAH6  APOE  CTNND2  S100A16  RABL5  PTPRG  DCAF4  TIMM17B  VEZT  RASGEF1C  LINC00152  KCTD17  MLX  TRPC4  TMEM68  GSTM3  AC093310.1  DDX54  KBTBD6  LOC648771  SSR4P1  MVD  ZNF706  TMEM205  LOC202781  NUDT18  GALNT14  RGS18  DDA1  PCBD1  ST6GALNAC5  AGBL1  CYHR1  PROSC  IL17RD  TRIM34  ICAM3  MTCH1  EPHA8  RP4-725G10.1  PDCD6  CCDC24  ALDH9A1  BAI3  C1S  HBG1  FZD2  XYLT1  EIF3CL  GRIK5  PODNL1  UBE2L6  SLC25A14  AMIGO2  PSMB1  TEKT2  LMO1  NEDD4L  SAMD9  SQLE  5-Sep  GPSM1  GRIN2B  NUDT2  LARP1  CYP2C18  ERBB2  PJA1  GGCT  DMWD  FAM127B  NISCH  CADM1  NCAM2  C1orf145  APBA1  C16orf75  C6orf52  TNN  RP4-788L13.1  AP001107.1  FOXRED2  VOPP1  C6orf222  SLC17A7  ACSM5  IQGAP2  MRAS  CAMK2D  GPR98  CGREF1  DHX58  RIC8A  ARL15  RCN2  PDGFC  LTBP4  ZC3HAV1  SCN9A  MKL2  TMEM158  SIL1  LANCL2  KCNK2  FNBP1L  TP53BP1  KLK5  KLK7  STOML1  CREM  AC092324.1  TM2D3  P2RX5  RASL11B  COL21A1  GPT2  MTPN  C17orf89  CCDC112  SNTG2  C1orf91  SLC27A2  EMID1  TNNI3  SAMD15  ABCA17P  ZNF572  SCARA5  DPH5  EFEMP2  CCDC52  B4GALT2  C12ORF75  MAGEH1  BLVRB  ROBO2  C20orf112  RHBDD2  SLC4A3  LOR  RFC3  PHEX  RRP7A  MAGED4B  PSD3  CPNE6  SNCA  SUSD1  ETNK2  PPM1M  PLB1  DAK  C16orf55  YJEFN3  PGM2L1  C16orf93  HN1  LARGE  STYK1  DMGDH  ZCCHC17  LINC00260  RSPH9  LOC100288743  SLC26A4  PHPT1  HPCAL4  KLHDC8B  RAB36  KIF12  GYPE  SLC1A4  B3GALNT1  RILPL2  WDR66  LOC642852  HTR1A  TXN  FXYD6  F12  FAM122C  TMEM159  TMEM238  CDH4  LRRC36 | SPRN  LAMB1  ZZZ3  SNRPD3  GABRE  PI4KA  RABAC1  CDC42SE2  TSPAN18  INTS3  ABLIM3  C15orf57  BRUNOL6  SYNGR3  NDN  TLN2  TRAPPC6A  ILF2  TCTE1  MPP3  ASB5  DOCK9  SPRED1  LOC646127  SELENBP1  MTHFS  MMAA  LDHD  APOC1  OAZ1  METTL9  ABCB6  GHRH  TOR3A  E2F3  COG5  UTS2D  GATSL3  C7orf70  AXL  DGUOK  PYCARD  LOC727967  LIPH  CCDC18  ABHD1  SNAPC5  CALU  TMEM208  FGFBP3  PPP2R3B  RYR3  C21orf110  ISYNA1  PARD3  CRIM1  SOX9  KCTD15  MOSC1  HRASLS5  NPY5R  MRPL10  TXNRD3IT1  RP11-29H23.1  MAOA  PALM  ODZ1  C7orf20  LOC644189  MT1A  PCTK2  EIF4A1P6  ERC2  LINC00338  ANKRD55  N6AMT2  SESN3  MAP7D3  PIK3C2G  DCTPP1  SH3BGR  PRKG1  LOC100287590  L3MBTL4  GRN  MPHOSPH6  IBSP  NSFL1C  TYRO3  INTU  PPAPR5  AC015871.2  HS2ST1  FAM105A  ATP2C1  TMEM9  LSM7  TRIM68  ADRB2  ITGB7  AHNAK  TNFAIP2  CHD3  AIFM2  C6orf221  TCTN1  PALM2  C8G  RFWD2  LPA  PLAC2  S100A10  HPCA  COPZ1  SRD5A3  SATL1  TRMT12  PALMD  MMAB  CTC1  WWC1  KIAA0649  SNTB1  GPR172A  DPYSL4  BRUNOL4  AC002563.2  STX1A  MCTP1  JAKMIP1  FBLN2  LOC649330  LIN7B  MYO16  CMTM4  SIRPA  GRAMD2  C14orf129  DLK2  PSD  CA11  DRD5  KCTD21  TMEM53  CAPZB  DHRS2  TUBB7P  MT2A  TCTEX1D2  CCDC8  KLF10  CYP2C19  PPM1F  HYI  LOC100287038  NCAPH2  SORBS2  MANEAL  SUMF1  DACT1  HSD11B1L  TNFRSF11A  FAM117A  KLHL35  DERL2  GNAO1  PACS1  AGAP3  FLJ45248  SLC16A8  BAIAP2  GPNMB  PAFAH1B3  SLC29A4P1  CYGB  HHIPL1  TMEM132A  C19orf12  FGD3  FARSB  ISLR2  AP2A2  MAOB  CALB2  C1R  DENND1C  SHC2  RP11-431H16.2  C17orf108  ATP6V0A4  HSP90AB1  DACH2  SEPN1  KIAA1024  FAM49A  LOC100129291  SNAP29  DUSP18  CNTN1  RPL39L  DNAH7  TTPAL  TMEM59L  C5orf49  CAMKV  WDFY4  MIF  PSD4  CSGALNACT1  C1QL4  TIMP2  PDGFRA  C9orf117  MIR2682  RHOC  ZNF449  PELI1  TBCD  EFR3B  LRP1B  LOC646627  DUSP9  C3orf31  AC007688.2  C10orf116  CXADR  PIM2  NIPSNAP3A  SLC2A4RG  RAP1GAP  GRIK2  DOK1  PPM1N  CAPG  LRRC62  DLL3  SLC2A12  YPEL1  SVOP  FAM26F  SPATA4  PCSK5  LOC100292909  WDR54  TUSC3  KIAA0284  GPC4  MGMT  LOXL1  AC005152.2  FAM113B  TEPP  CANX  MYB  ARL10  CRIP2  RP11-63L7.1  CCDC90B  C22orf39  SYTL5  SNHG8  SYN2  HES5  PDGFB  RPA2  AC011479.2  GNPTAB  SULF2  FAM184A  OBFC2A  E2F5  LSM4  NAGK  AGXT2L1  MYCNOS  DNAH2  RNF7  DMC1  SRM  ENO1  C21orf67  SLIT3  UCHL1  PMVK  GLT8D2  CACHD1  RAVER2  C21orf56  C6orf1  RNASEH2C  GPR160  TMEM106C  PEA15  RNASEH2A  SLA  LENG4  PLCB2  AC015936.3  C12orf73  AP000654.2  PCDH15  SUB1  FABP7  CXorf57  SMARCD3  FAM148C  RASAL1  PTPRF  ARMC10P1  GABRA3  GMFB  TRIM27  AP3S1  CENPVL1  IL12RB2  PACRG  CMTM1  C1orf230  CFD  MGST1  C11orf66  DAPL1  ZCCHC12  MAPK3  CHID1  CTXN1  TMSB10  BRE  KIF21B  MEIS3P2  LYRM2  PYCRL  SLC7A4  TCEA3  PSENEN | EDF1  RASA4CP  CCKBR  STRAP  LINC00158  PRR7  SLC27A3  RSU1  NAA11  AL117209.1  THRB  C22orf29  CLEC4A  ADRA1B  STOX1  KCNE2  TLR4  DGKB  C6orf70  C17orf61  MSH5  CCND3  AHCY  C19orf70  SCN7A  C2orf70  RIN1  CBFB  RPS18  RBM24  LOXL3  HCST  PSMA6  AC011498.2  KL  RAB32  TNS3  SPANXD  VLDLR  ADH5  GAMT  USP7  TERF1  ZRANB3  SC4MOL  AMZ1  SFT2D2  GUCY1B3  ARFGAP3  MINK1  LINC00461  NEUROG2  RP3-410C9.1  LY86-AS1  GGCX  SAMD3  UBE2D2  ALDOA  CACNA1H  RLBP1  LINC00205  DLX2  SDC3  SLITRK2  STK32A  CD163  TMC5  LRRC24  NEK6  ASPG  ABI2  NPY  LOC100291229  KCTD2  C7orf13  MGC23284  LDOC1L  WDR17  LOC100287260  ME3  KRT86  PRODH  NUDT3  FKBP1B  SCIN  P2RY13  KLHL23  PPDPF  FAM128B  GLI2  SYTL2  JMJD4  FAM19A1  GALNT10  SPRYD4  PHF13  NAV1  FAM160A2  ZFAND3  VAMP2  PLXNA1  C9orf98  CEP72  IL1B  SERP1  VDR  THSD7B  C16orf58  GRID2  PABPC1L  LOC729683  MRPS6  CD83  PCK2  ERLIN1  XKR4  DMD  GSTM5  ANGPTL6  PRPH2  IDNK  C3orf54  PNPLA4  ITFG1  RP11-45B20.3  NCRNA00164  ASTN1  DSEL  APBB1IP  ARPC5  TMC8  GALNTL6  ANKRD43  LOC727938  MBOAT2  DOC2A  TCP10L  PCDH1  SNCG  SMPD2  DAP  TCF7L1  SKAP2  SLC7A3  ZNF497  ZSCAN18  TTYH3  SLBP  NOVA2  ANKRD19P  FABP3  ARF3  ACOT12  ENTPD6  TRIB3  AP000812.2  SNX2  FAM71F2  ZBTB8B  BEGAIN  UROS  TSSC1  C2orf73  KIRREL2  ZNF436  DNAJC30  FDFT1  KCNG3  TOM1L1  ZWILCH  C17orf79  SLC29A4  C19orf76  MRPL23  SEPW1  SYNPR  CRYM  LOC439914  NDST4  CES8  NTSR2  DIAPH2  GPI  ZNF350  IGSF22  ZMAT1  CCR1  PDLIM5  C14orf79  MPV17L2  GLUD2  WDR23  NOL4  ASXL3  TTYH1  KCTD4  HTR3A  ANKLE2  KCNMB4  NPTXR  C10orf11  HPCAL1  YWHAH  LOC100289341  DDX25  GSS  EFNB2  WDR77  MARCKS  PRKAA2  AOF1  STK32B  METTL5  RP11-60I3.5  DHDH  LCN12  PRAF2  TRIM17  AC079776.2  CYP4Z1  CLPP  DCAF7  DKK3  CYTH4  GPRIN1  SLN  FADS3  MRPL49  RP11-33N16.1  FAM102B  SARNP  CRMP1  ARPP19  RP11-834C11.3  ASH2L  CD24  RGS14  NUTF2  ZDHHC12  FCGBP  ACOX3  RALGPS2  MAPK8IP1  THRA  MORN4  SRRD  POR  ISOC1  LOH12CR2  CAP1  IFI27L2  PDE2A  TAF7L  CACNG3  ATOX1  DNAL4  CHCHD6  COCH  PTPLAD2  DOK4  AKR7A2P1  WDR6  NUDT16L1  PYDC1  RBP4  TIMP1  ZCCHC18  PTGER3  SNX26  COG1  CNR1  GLRA2  C8orf30B  CDH9  ZFP64  TMEM176B  DMRTC1B  SP5  IGFBP5  PCDH19  TIMM8B  CD99P1  GABRB3  RAB8B  NNAT  LOC653051  TUBB  GNB2  POLR2L  CORO1A  BIRC3  DNAH5  DOK6  TMEM17  LOC100287146  PRSS23  VPS37D  MSANTD1  LY6H  FKBP1A  LOC441052  SCPEP1  GRIN3A  KIAA1644  NKAIN2  PRKCG  C2orf80  MMD  SCN3B  LRRC3B  B9D1  C6orf173  ARHGDIA  MAN1A1  SSTR1  SLIT1  GDA  GNG2  PGA3  ANKRD50  LOC100131504  PKIA  KLF8  SNX7  UCHL3  NIT2  MOBKL1A  KCNN3  FAM71F1  LOC100287347  TUBB2A  IL33  PYGL  GABRA5  PID1 | CACNA1S  TCF20  AKIRIN1  SFTA1P  ZC3HAV1L  CCDC123  MAN1B1  FAM183A  RP11-369J21.1  C11orf83  KRT83  MEIG1  DGKA  HEYL  ZNF883  CCDC56  NXF2  WFS1  C13orf18  CD8A  USP27X  TMEM136  AAED1  GBA  CYB561  MGC45800  RSPO4  ALCAM  PPAT  C1orf53  PHYHD1  MMP28  PARVB  UBE2I  ZNF667  C3orf17  B3GAT2  LHFPL3  PFN3  PLA2G15  MLC1  ARHGAP15  GALNTL4  SDK2  ADRA2C  ENOX1  SLC44A2  GPR6  NHP2  TMEM18  CD81  C2CD2L  HILS1  IQCJ  C1orf128  FKBP14  TSPAN14  MORN5  LRRC2  CSNK2A1P  MRPL37  BCL2A1  AC069029.1  ERI3  TBC1D10A  PRMT2  OPHN1  SRGAP1  VIM  ANO3  TRAIP  APOM  LILRB1  PRR5  EHBP1L1  PTDSS1  WBP11P1  CYP2D7P1  SEC14L2  WDR13  FIGNL2  EYA2  PSMD8  DDX20  SYT10  CPEB2  PDE1A  DTNB  CDCA7  CHSY3  ATP6V1C1  ZNF528  FABP1  ADAMTS3  B3GALT6  MAP1LC3A  XAGE1B  SND1  MAZ  C3orf78  BTBD9  MTMR3  CCNH  CNTN3  HBG2  GEMIN8  LOC145837  SH2B3  GABRG1  AK5  CCDC37  OGG1  MPP2  SMAD1  RRAGD  SAMD14  CDC34  LYVE1  LOC550643  EDARADD  GUCY1A3  LOC100127891  ACVR2A  LDOC1  GPC6  UBE2A  C6orf154  KLHL12  TRIM22  RNGTT  TMEM66  TMEM42  IGF1  SRR  AC013283.1  MAPK11  MAN2B2  CXXC4  TNNC1  DOCK7  CCDC85C  C3orf52  ITPRIPL2  ARHGAP18  A1BG  PTHLH  LGI4  CHMP1B  LINC00239  C7orf31  IQCF3  PSMB2  GNMT  GJA1  MAD2L2  MAST3  GNG10  SLC8A2  TBPL1  KLHL1  CDH10  LOC728739  PUSL1  NUMBL  GLP2R  TXNL4A  GABBR1  ARPC4  KIAA0947  RABL4  HTR2C  BFSP1  LMAN2L  TUBB8  AC026369.2  LOC100130054  LRPAP1  MED24  MSX2P1  UFC1  LYPD1  TTC18  NUS1  STON2  C1QL2  SGSM2  MEA1  ZNF239  SIN3B  GLRA3  YWHAZ  NAV3  MYRIP  RAPGEF4  OXCT1  FAM110C  C5orf33  IMMP2L  NPM3  ZG16B  EXOC6  SEL1L2  RGS22  AKIRIN2  POTEKP  MDGA1  ODZ3  C19orf10  AP1S1  KLHL2  C1orf50  SFMBT2  SLC35F2  LOC100129309  EIF4EBP1  VPS37C  LUZP2  DNAJA4  CYB5D2  LINC00238  TMEM47  GSTO2  SLC25A23  MR1  KNCN  AP2B1  CYP2D6  GNG4  WNT4  ABI1  GRAMD4  B3GNT4  PLXNC1  MAPK1  FLJ38379  POLE4  FAM127A  ARMCX1  SUMO3  CYP46A1  RRAS2  BRMS1L  C2orf69  MESP1  PTRH1  LSM3  EDNRB  MYH7  EIF3S8  HBQ1  LRRC16B  CCDC148  RPS27AP11  ROBO1  SCGN  NPY1R  SNRNP27  CORT  ACTG1  ICAM5  ACOT2  TMEM176A  HRH1  DNAJC25-GNG10  DPYD  DDN  C13orf36  AC093734.11  UG0898H09  ARHGAP28  RGS20  PTPRA  TDRD9  ECHDC3  UBL5  STK32C  CDKN2D  C12orf45  PSORS1C1  SEMA4F  ARMC10  FAM171B  MAGED1  TSPAN16  RRP7B  RNF150  WDR86  WNT10B  NKAIN4  SYT17  DPYSL3  NUDT11  C1QL1  RAB27B  DYDC2  PNCK  PPP4R4  ATP2B4  ANKRD6  ATOH7  CPNE7  PRKCD  AC006273.1  C1orf95  AC044839.2  FAM65B  GABRB1  EFCAB1 | DBNL  C6orf225  DLG4  SMPDL3B  GIT1  ZNF229  P2RY6  CES7  KIAA0195  MDP1  DECR2  FAM126A  SLC16A9  C1QA  C2orf39  ZNF577  UFM1  RNASE6  PPAPDC1A  RP11-544L8__B.4  MYOM1  PAMR1  KIF22  RHPN2  LY86  CHAF1B  EIF4A1  NCAN  ZER1  VBP1  PBX4  WDR92  PCLO  TRHR  AHR  FBXL13  ALOX5  STIM1  RP5-990P15.1  C1QTNF2  FAM107A  HERC5  FZD8  VPS26B  ACVR2B  DUSP26  C17orf58  DNAJC2  CELF3  LOC100127925  PPIH  BRF2  EXOC4  ALDH7A1  CNRIP1  AKR7L  ADSL  GOLGB1  C17orf96  C20orf27  SLC44A3  TP53I3  NBL1  AC099524.1  TBCA  LGALS13  LMO3  LINGO1  SBF2  TMSB4X  CD5  TMEM219  ATXN10  XRCC6  PBXIP1  KPTN  FBXO15  LRRC61  CARD17  LST1  LPAR4  C4orf32  ARPC2  C11orf84  FBXL16  BTRC  PSME2  CAMSAP1  LOC100131482  C21orf70  CCDC41  MAP2K1  C1orf187  ANXA11  C4orf45  KIAA1328  CLDND2  DIRAS3  BDNF  KCTD6  ARHGAP6  SNAI2  LRRC57  PTCH1  VPS37B  ACCN1  RIMBP3  HRAS  DPP10  TUBB2B  ANKRD30BP2  SCN3A  TNFAIP8L1  C3orf14  COMTD1  PTGES  GNB4  IMPACT  SLC26A11  ATP6V1H  CCT5  C11orf70  FAM40A  GSPT2  WDR1  FTL  APOC4  MTHFD1L  NPDC1  YKT6  GPC5  PRDM12  CRELD2  HSPA13  MACROD2  RPL29  CPNE5  AKAP5  CD84  CDKN2AIPNL  HSBP1  METTL6  CTSO  SEMA3D  HRASLS  STOM  WIZ  GRK6  CIB2  HSP90B1  LAMA1  CDC42  PYCR1  FAM132A  INSM1  ZMYM3  PNMAL1  TNKS2  IGFN1  JUN  CMAS  LOC147727  SAP30BP  TIMM10  RPL36  ERCC2  NPB  CCL28  LOC391722  IGSF10  MGAT4B  BLCAP  LRRC56  WDR16  CETN2  EVI1  SPRR2G  SHCBP1  NME4  TH  GRIK3  FAM171A2  FLJ40712  RXRG  VSIG4  NELL2  ADAP2  TRIM36  ACADL  MRC1L1  ARMCX2  SHANK1  LOC644172  DHRS12  SLC27A5  SCAPER  C1orf228  CPLX3  LOC100289263  TOX3  MYL12B  CCNA1  TBC1D26  LOC653513  RP11-565H13.2  SEMA4A  C10orf32  PKIB  LOC100133211  CASK  BATF3  GHR  PLEKHC1  ACTR10  MAPK1IP1L  BEX2  LOC442308  NLGN3  C16orf73  ZNF177  BCR  C6orf26  AL589182.2  DDAH2  EGF  ACSL4  RASL10A  WDR51A  C12orf53  WDR69  NDUFC2  PNMT  FARP1  CAPSL  MMD2  DYNLL1  TMEM108  RSPH1  CHST1  DCLK2  MED16  DUSP13  GLOD4  PTGER4  IL13RA2  TSEN34  LOC392145  PTER  EXD1  SPC25  RP11-544M22.1  NOV  KIAA1244  PXMP4  AC078937.4  NTSR1  AC069234.1  FAM149A  DCAF15  TMEFF2  LOC100288911  AC093283.3  TMEM200A  GOLM1  EFNB3  CXXC11  RAP2B  RGMB  NANOS1  FILIP1  SLC16A2  PPIL6  AK3L1  DNAH14  NT5DC3  SSTR2  ARRB2  SST  LOC100287080  NUDT10  SGSH  C12orf23  CHMP1A  NHLRC1  GRM1 |

| **PLS1 positive genes** | | | | | |
| --- | --- | --- | --- | --- | --- |
| ASB13  OSBPL6  RP5-1022P6.1  KCNAB3  JDP2  LOC100286909  SYCP2  DCUN1D2  JHDM1D  ANK1  ZMAT4  EEPD1  BHLHE40  THAP10  LOC256374  ATP2B2  EPB41  ESRRG  A26C1B  SLC38A2  SLITRK3  RP11-403C10.2  KCNT1  LAG3  TTC39B  LOC100288939  RPGR  RCC2  PHYH  MAP3K6  RHBDL3  TMEM86B  PIM1  ZYX  CDR2L  DCBLD2  SCN1A  PIK3CA  DDHD2  UPP1  MOGAT1  GLCCI1  LOC100131943  KCNC1  VAMP1  WASH3P  ALX3  SNHG1  USP13  ANKS6  AC131238.1  ABCB7  ZNF193  MTRF1  SLC45A4  TTBK2  PPARD  EIF4A2  SETD7  ZDHHC5  OR2L2  LRRC49  SYT12  MAP3K13  CHIC1  GPR89B  RAG1  LRRC37A4P  TGM1  GABRD  C6orf167  TNFRSF25  PURA  RP11-422J8.1  CACNA2D2  HR  GCC2  RP5-1068E13.3  OR2L13  CCR10  IQSEC3  DPY19L2  C9orf45  ECM1  SLC16A6  TCHP  FAM188B  SRFBP1  PINX1  FCHSD1  KCNA1  SLC35B4  TNIP1  GTF2A1  CADM4  TPRKB  ZNF555  KIAA0564  ANKRD34C  SEC31A  FBXO33  MRPS30  CASP8AP2  APOL2  ERCC4  HLTF  FER1L4  ATF4  GFRA2  KREMEN1  LOC100131471  ATP8B1  DCPS  ATXN7L2  AFF1  NAT8L  FMN1  PLXDC1  GPR180  OSBPL2  ADCY9  AR  FAM155A  ERRFI1  CHML  SORBS3  KTELC1  KCNB1  MAP2K3  PPP3CC  SORL1  ADCY1  CUL5  HUNK  AC091565.3  LIAS  PGBD4  PNKD  DENND4B  LOC100292489  C6orf106  SCAF11  PLCL1  COX10  ARL13B  MX1  GNAS  LMBR1  6-Sep  CEP135  AC126544.1  GZF1  IFFO2  AC005277.1  SAV1  WHAMML1  CS  DENND4C  TAPBP  PPIEL  SYT3  PLAGL1  ZFYVE28  SAT2  RANBP6  ZBTB16  ZMYM5  NEK1  ZNF684  CUGBP2  AK2  DUSP1  INPP5B  C3orf37  MC1R  RPP38  NOB1  BRSK1  PANX2  PIP5KL1  ZNF324B  CA13  AC005512.1  NFXL1  EMILIN3  SYNE2  TAS2R45  EDEM3  NUDT15  GRIN2A  KIAA1614  SORT1  ADM  AUH  CCDC109A  MBD2  ZNFX1  FAM88A  TAP1  TBC1D8  PCDHB16  TRPM7  CNOT6L  AAAS  ATXN1  PRDM2  ATAD1  LOC100127998  AL096711.2  EFNA5  C1orf64  IVNS1ABP  C6orf115  ZNF782  KANK3  LOC401357  ASTN2  NECAB3  LAMA4  CLK4  KIF21A  WDR4  HDGF  DACT2  XYLT2  PCDHB11  EMCN  NUAK1  PHF6  KIAA1199  RPS6KB2  LDB2  GAPVD1  COX7A1  RAB11FIP5  DNAJC25  NFKB1  PNMA3  ZNF718  THAP1  CIT  KIAA1712  DHX15  ZSCAN29  HRC  SLC25A15  HELB  TFB1M  MAP3K7IP3  RANBP17  ERCC5  C14orf83  RAB11FIP2  NIPAL2  FIZ1  LOC100294059  ZNF383  PPP1R3E  AC005077.3  PDCL3  DAZAP1  STK3  SLC47A1  RRS1  UBE4B  HAGHL  KCTD9  UBLCP1  GIGYF2  SLC9A3R2  NRIP2  MED21  TOP3B  GALR1  TADA2B  PARP3  FNTB  KIAA1324L  MRPL19  VEGFA  XBP1  MTFR1  ZMYM4  HIBADH  ZNF75A  PCNA  BCL6B  ALKBH8  TRIM58  FAM123B  VPRBP  PRMT10  ABHD11  AVPI1  TAS2R5  ZNF692  C1orf199  PAXIP1  WDR8  CFHR3  CLK2P  C5orf48  ZNF143  APOL1  AC141586.5  AC091878.1  PLCXD1  ANKRD42  SPSB1  HSPB6  TBRG1  TAS2R8  LOC286434  PRKAA1  VCPIP1  PARG  CLDN5  PKN2  MOB2  SEC62  PPM1L  FBXO28  SLC4A9  PLS1  AFAP1-AS1  ATP1A1  PCTP  MYO1E  PCDHB8  TBX3  NOL10  RP1-21O18.1  LDHB  ABCA7  HERC2P4  FLJ14186  LYNX1  SLC2A6  ADPRHL1  CSTF2  FGR  LINC00346  ZNF500  CHAF1A  FBXO30  TMEM132C  SLC20A2  BICD1  MREG  PRR16  ZNF800  ALDH1B1  PCDHB9  UPB1  ACVR1  SPTBN5  SIAH1  EPHX4  CRTAC1  ANKZF1  HTATIP2  MAP6D1  SLC10A5  FAM209A  ANXA3  CLEC2L  BBS1  ENG  PP13  CASP2  MEGF6  KIAA1267  KIAA1109  LRRC43  CCNO  STC2  CTSC  CROCCP3  LRP5L  PNPT1  BTN3A3  BOLA3  SPRY3  ITGA11  FLJ40292  CYTSA  SMC1A | AFTPH  SLC25A12  C1orf201  CORO6  SLC16A7  NCOA3  ABCA6  SEMA7A  SLC25A37  NR3C1  CHGA  SCN1B  FAM71E1  HIST1H3A  ZNF385A  SPTSSB  STRC  MCF2L  POU6F1  LOC338620  GABRG2  TBC1D30  IDE  STS  KLF9  CCDC58  TRPC3  HERPUD1  SLC4A8  GRAMD1B  RNF148  LPCAT4  B3GALNT2  PLEKHH3  CPLX1  ZBTB1  ANKH  ZNF295  PANK1  GLS2  HTR1F  PIK3CB  JARID2  NFKBIZ  FZD6  LYPD5  C1RL  NCKIPSD  KNG1  TFB2M  PRICKLE1  PDE4A  KLF12  MAP3K9  ROCK2  LOC643669  CCNB1IP1  SLC19A2  GAS2  GAS6  BMP4  BRAF  GABRA1  GPR158  SLC9A1  ARHGEF7  DQX1  SRPK1  AC013402.2  MTUS2  SCN8A  LOC440459  CHRD  ZBTB38  ANKS1A  ABCD2  HIST4H4  SLC39A8  NFKBIA  CAMTA2  HCN1  DUS1L  PFTK1  LOC100128174  ETV4  HS3ST1  SLC5A4  GTF3A  SERPINB9  SEMA6D  C10orf140  NARS  RP4-695O20__B.1  EHMT1  ZNF385D  ATP2B3  DCLRE1A  PXDNL  TOP1P2  PAR5  FOSL2  LINC00473  LOC100133150  HELQ  OSBP2  ITGB3BP  STIM2  UBAP1  CCDC89  MBNL2  TAS2R9  KCNC3  NFIX  RHOBTB1  FGF14  B4GALT6  ACSL6  CALCOCO1  PIM3  PREPL  LOC283683  PCGF1  C4orf49  SCAMP1  ZCCHC6  FKBP5  RPP25  THEM4  LRRC4  LMLN  NR2C1  NDUFS1  ZBTB26  PRH2  LEPROTL1  MTPAP  LOC729852  KCNK12  TMEM14C  ZNF740  KCNQ5  SLC38A11  WHSC2  EIF4G3  ZMYM6  HIST1H1E  FLJ23867  PAIP1  PPAPDC3  C18orf15  LOC731275  HES6  BRP44L  C3orf18  SH3BGRL  RP11-61L23.2  MTP18  FBXO9  CPT1B  RGPD4  CD274  IGLL1  DUT  SLC25A25  HCN2  PKNOX2  ARID2  MPHOSPH9  PPP1CC  RECQL  HISPPD1  NCAPD2  MYH7B  ADAM23  CHD2  OXGR1  NOM1  NGLY1  FANCM  NEB  H1F0  ZNF250  ZFPM2  ATP13A3  EML2  KIAA1033  CD2AP  SPG20  RERGL  TGFBR1  C6orf138  ARIH2  EPB41L3  FLYWCH1  SPAG16  USP28  EMB  EPN3  RWDD2B  PSME4  LINC00472  SLC35B3  ADSSL1  EEF2K  CCDC69  FAM126B  PCNT  CHD9  CDC42EP3  GMEB2  SEC14L5  PCDHB18  ECSIT  PRB3  RP11-206L10.11  TMEM35  MRPL39  ZNF20  SRSF1  C8orf41  ZFP3  PIBF1  RUNX2  PRKAB1  NOP14  TAS2R31  LOC100289454  RHOQ  TBK1  BTBD8  PFDN1  ACTR1B  ZNF41  ATP6V1G1  CMPK1  PYROXD1  TNRC6A  LGI2  ATP2B1  RABL2B  HECA  KLHL11  C6orf162  CEP192  LOC285359  RASSF8  LOC642513  DPY19L2P4  GLA  CCDC91  FAM20C  PTPRE  SOCS5  MRPS31  FAT3  NGRN  CLIP4  MTERFD1  ETV6  KLHDC6  EPB41L1  JAK1  PTH1R  KCNA6  TRABD  LOC730234  ITGB1  FLJ42627  NSMCE2  PHLDB2  C12orf54  PCDHGB1  TAF2  ROS1  ZNF543  ZNF333  PDCL  DTX2  MKLN1  MIR133A1  PDE10A  MRPL48  FAM167B  CRKRS  C19orf61  STRN  LOC389834  ZBTB39  ANKRD20A1  C1orf172  ZBTB8OSP1  TMEM145  C17orf56  GSDMB  SERGEF  SPTBN4  LRPPRC  CEND1  SHISA3  GSTA1  KCNJ11  ARMC8  IKBKB  ZNF567  ASAH2C  DPY19L1P1  PRSS16  IMPDH1  C1orf114  KBTBD8  TMCC2  C20orf74  EMILIN2  ZNF557  C16orf46  ABHD13  CCDC96  INHBA  CTTNBP2  CCDC125  CUX1  RMND1  CDR1  ABCG4  PDC  BMP1  EIF1B  DDX55  RAB14  SULT4A1  FAM104B  TOB1  COL4A4  ONECUT3  RARA  PEG3AS  SLC12A8  TMOD2  GLTSCR2  GSTA3  GHRLOS  ATPAF1  C9orf96  SEPHS1  CNOT8  LOC100287813  FAM78B  ZMYM2  CHN2  LOC10049948 | FBXO32  TTC21B  CLGN  LCA5  FNDC5  UCHL5  PIF1  SLC39A14  OIP5-AS1  RFX5  AGPAT9  EIF2A  C1orf71  LOC100288893  ANK3  VPS36  INPP5F  CMYA5  TPTE2P6  BBS7  RELT  FASTKD1  CEP152  SLC5A6  GPR161  IPW  GPLD1  INTS9  STARD5  EXTL2  MAT2B  PPARGC1A  NR1D2  L2HGDH  CACNB4  TAF4B  UBE2D1  KIAA1107  PLCB1  IL28RA  STAG3L4  BID  COG2  PVALB  GK3P  TDRD3  RRM2B  RET  C17orf75  KIAA0240  EFHA2  C16ORF52  LRRC8C  IFFO1  ST3GAL6  BCL3  SNAP25  RHOBTB2  C1QTNF1  CERK  DPP8  PLCB4  FAM35B  TBR1  GOLPH3  PASK  ALDH1A3  RACGAP1P  USF2  ARHGEF18  C1orf97  PCP4L1  GNRH1  INA  MAP9  STXBP5L  C9orf82  RHOXF1  C11orf75  FAM49B  C20orf177  DDX11L5  CDKN1B  LOC642393  C6orf204  KRIT1  RASGRF1  IGLL3P  SIAH2  FNDC4  ACVR1C  EFR3A  ASPSCR1  PDP2  SLC25A17  AP4E1  OR2L8  RCC1  EIF4E1B  CACNG7  NKX3-1  RORA  EYA4  PPIL5  AC024560.3  ATRNL1  TMEM188  KLC4  C5ORF62  ZNF365  ZNF175  ZNF35  GK  FLT1  LIN9  KLHL24  SLAIN2  ABCB10  STARD9  C10orf118  RP9  BTBD11  C22orf25  SRL  TRMT11  EML6  DENND5B  GFM1  GLRB  PKD1  NCRNA00085  SIPA1L2  TIAM2  SCAI  ZFY  LGI3  METT11D1  MADCAM1  MAP4K2  MSH4  SLC46A3  LTV1  C18orf21  STK40  UHRF1BP1L  EIF1AX  INPP5A  FAM133B  JRK  FAM57B  C13orf34  GNGT2  ZNF519  MFHAS1  ING3  AC084125.1  C1orf156  DCAF13P3  TCOF1  STX8  VIPR2  NIPA1  TNNC2  MIAT  RPH3A  CACNA1A  BCO2  SLC26A6  DUSP  STAT4  MITF  HAPLN4  EPS15  SLC38A9  GPR89C  ADAM9  IFIT5  DCAF8L2  HEXIM1  SKIL  SLC43A2  QRFPR  SEC14L1  FGF5  CDADC1  PPIG  PDCD6IP  MAGEL2  CLPX  P2RX6  ZBED1  HELZ  SLU7  PDE7A  PDP1  ZDHHC17  NXPH3  BRWD3  SLCO4A1  AC005041.9  ULK3  CDKL1  PLAUR  CCDC136  MAFB  GPR146  ACTN4  KIAA0408  GLRX2  NT5DC1  PRKCA  UPF2  RIMKLA  ANKRD13A  TRIM26  PIK3R3  A2BP1  ZCCHC10  TCHH  EFCAB7  C18orf42  MBTD1  TANK  ZBED4  ZFX  ZNF146  KPNA5  AC022311.1  DDX4  SACM1L  ARHGEF10L  TTC39C  C9orf80  KIAA0831  FKBP4  KIAA0430  ZNF132  UBE2O  13-Sep  SDHAF2  C2orf27A  HS3ST5  12-Sep  BRUNOL5  LARS  PPP2R5D  KCNC4  PPP1R12B  PES1  SLC9A5  HIST2H2BF  DIP2A  LEF1  ETS1  PIAS2  C12orf32  TAF12  C20orf39  SPOPL  C4orf18  PCDHB10  C5orf56  ANGPTL2  ADAM20  C18orf8  PIP5K1C  TMEM150A  TMEM178  SPAG6  MRRF  WASH2P  ATP5A1  SLC38A3  ZNF586  GTF3C2  C12orf68  TICAM1  ZNF823  DDX46  SETMAR  BAAT  GPR124  OSGIN2  TRPM2  IGFALS  PLAG1  PLEKHG1  MNS1  LOC729020  C4orf22  CCBL1  PRH1  FAM178B  TAF13  ONECUT2  CEBPZ  CCDC49  RILP  RERE  RUSC1  ELMO3  ZNF252P  C12orf5  WWP1  PPP2R2D  SPOCK2  MYOZ3  C9ORF174  MAD2L1  NPAS4  NUDT22  APPL1  CCDC40  ESAM  TMEM41B  USP44  LOC732327  TMEM184C  SPIRE1  GGT5  MBD3  PCMTD1  TTC26  C19orf66  ATP6AP1L  KCNRG  GRINL1A  GOLGA4  ITIH5  TRIAP1  CDK2AP1  TNF  SPIN3  IRAK1  SACS  MCF2L2  ACIN1  USO1  SFXN1  GLMN  AMD1  OGFOD2  CLEC4M  NAAA  KLHL21  TBCC  ELMOD3  FAM116A  STEAP2  C3orf23  EGFR | LOC254559  CRHR2  MYBBP1A  EIF4E3  TLE2  XKR6  CADPS2  ATG16L1  DCP1A  SERTAD4  INTS4L1  CNNM1  LOC158696  CAMK2G  KCNJ3  C12orf24  MID2  DHX35  DPP4  ZNF165  PECR  AS3MT  HIVEP2  CDS1  NT5M  SCLT1  LOC283788  ZFAT  MCF2  MRGPRF  SCRT1  HSPA4L  RAD54B  POSTN  TUBD1  C6orf47  C6ORF228  TRPM3  C4orf33  ZBED5  CLEC16A  MYO19  DSCC1  ZSWIM4  C9orf72  FRAT1  DMKN  VWC2  PPIL3  NKAPL  TRIM52  RSRC1  KIAA1841  FBLN7  GLB1L3  C5orf13  MCF2L-AS1  RNF157  MRPL16  TRERF1  RICTOR  GRPEL2  NCK2  IER2  TRIM37  ENTPD3  SHD  TMPPE  SLC12A5  RAB37  FAM43A  CCDC146  PPP1R28  RCL1  PACSIN3  FLYWCH2  ZBTB7A  PLCD4  GDPD1  SGCG  TSC22D1  SLC25A36  VAV3  TAS2R14  GPBP1L1  FAM115C  MFAP3  MGC16025  TCF7L2  LOC728743  FLT3  ATP6V0A2  DEXI  SOHLH1  C11orf52  SHROOM2  ARID5B  KDM2B  CABP1  RG9MTD2  C14orf104  ZNF394  PDIK1L  TBC1D19  ZBTB8OS  C12orf35  SLC22A23  LMTK2  MFAP3L  ENPP5  KIAA0182  SLC39A13  ADAMTS13  C6orf211  MRPL34  IL15RA  UBL3  CDH7  CKMT1B  ZNF182  C20orf20  C1orf66  ITM2A  TAS2R4  LEAP2  LOC100133161  PMEPA1  SDHA  FAM108A4P  CCDC126  WDR70  AC110754.1  OSGEPL1  ARHGAP26  DIS3  PPTC7  ZNF443  STAM2  KIAA0427  PARD6B  GADD45B  XPR1  HOMER3  CPEB3  IFT172  RASSF4  FAM190A  ZNF774  CITED2  HAR1A  ATP1B3  PTPN12  TMEM49  C8orf31  MRPS5  HYLS1  GPR108  ST8SIA5  ARFGEF2  PRIM1  REPS1  YARS  MYNN  IQSEC1  FAM108A4  ATG4D  RNLS  PPP1R15B  MASP2  LOC728543  ODF2  IER5  RP11-78J21.3  IGFBP2  RBM41  FGF18  MRPL35  LRRFIP1  CHORDC1  APIP  EIF4G2  ZFYVE19  ATP1B1  ITCH  SDHAP1  OXNAD1  TET1  ZKSCAN4  BCAT1  ADORA2A  F7  MSRB3  TXK  TTC39A  HIST1H1D  TUBGCP5  SHMT1  SGOL2  KRBA1  ALG2  PLEKHA6  PLAT  GPR85  TACC3  MIER3  MRPS26  CACNG2  FDX1  ZNF224  ERMP1  HS6ST2  ASAP1-IT1  DFNB59  CRISPLD1  ARHGAP11B  KCNJ14  MYLK2  SLC25A44  AMH  OPN3  TRIM4  USPL1  TAF1D  IL6ST  PAQR9  LOC100288083  PCDHB15  WDR91  ZNF124  ALDH1A1  AC100803.1  OR2AK2  CAMKK2  CABLES2  PTPN2  NLRX1  TRIM32  COX17  ELMOD1  WDR75  TBCEL  TRNP1  ATG4A  ZNF230  ZNRD1  HSN2  SPHAR  FLJ37798  CCDC78  LAMC1  GCKR  ELAVL4  ASH1L  NR1H2  KIAA1586  LOC100129616  GRIP2  FOXA2  PPPDE2  SLC20A1  RP5-1187M17.1  FRMD5  GALNT3  SDSL  TESC  ZNF407  SENP7  PVR  TTLL6  NFATC2IP  ZCCHC4  VSTM2A  MIB2  THAP3  AC136632.1  ARIH1  SNRNP40  C8orf47  LOC100289408  MTRR  PALB2  LANCL1  ALS2  FTSJ3  CAPS2  ARID4B  ZNF672  LOC729839  FAM108B1  SLC6A11  ZNF181  MAGI3  ZC3H7B  C16orf45  PION  MCM8  MAP2K7  DFFB  NEU4  MDGA2  BAG5  ITPK1  WDR42A  NAP1L1  C1orf124  RFPL3  ZNF366  EME1  CTSZ  RNF133  MRPL50  SORBS1  TCERG1  RP9P  CHN1  KDM6B  SNCAIP  PPP1R3B  ZCCHC8  ZNF620  RNF208  SPARCL1  MRPS36  MANSC1  SOAT1  GLRX  DOK3  SPATA22  SLC38A5  DHX40  MUSTN1  IDH3A  ASGR1  NMD3  THAP2  CLOCK  RNF144A  ZNF670  RBM4B  FAM120B  ZNF625  GHDC  MUC20  HERC6  SATB1  EPM2A  MMP15  SESTD1  ABHD6  IL7R  FGD5 | SLC38A1  HECW1  JAM2  PRMT7  OVGP1  KIAA1456  MRS2  KLHL15  BHLHE41  MRPL33  TRMT61B  PCSK1  CDC42BPA  CCL27  CPSF1  MKNK2  LOC149837  ABCA9  STRBP  AADACL1  TSPAN9  ST8SIA1  CAMTA1  PLA2R1  TBC1D4  OSBPL1A  FGF9  TIFA  SIX4  NDRG3  CCNI  RNF113A  EIF5A2  C3orf35  C1orf133  FAM131B  DDX3X  ATP4A  DENND1B  LMOD3  LUZP1  INTS4L2  MTAP  NEXN  TAS2R13  ADAM22  EGR1  STAC2  VPS54  ZNF385B  FSTL1  SMARCD2  RASD2  RFPL1  FAM83D  C11orf61  TRIP11  PRRT3  CNTNAP1  MGST2  CROCCP2  LOC100286977  SUZ12  ESCO1  ENTPD4  UBA3  EIF2C1  ZNF225  KBTBD3  RTKN2  ANKRD37  PDS5B  ACSL3  ST6GALNAC1  KCNA2  NFIC  ACAN  USP45  PAG1  C2orf49  KLHL20  C10orf2  KCNS1  ARC  LAPTM4B  RMI1  MPP1  PRDM5  SIRT4  ABCC1  CLK3  RWDD1  RALB  TTRAP  ZNF630  AMMECR1  AQR  CORO2B  REEP2  SETBP1  HIST1H4I  ARHGAP9  AAK1  MDH1  TRIM13  GPATCH8  CWC22  RBMS1  PRR4  ELTD1  NAALAD2  POLD3  TAS2R48  USP33  ZFYVE9  DND1P2  CSDC2  ANKRD62  SUPV3L1  NOP2  RPS6KB1  AIFM3  PDZD8  CKAP2  SAPS3  SLC36A1  CLVS2  ZC3H10  GSTT1  RNF168  LOC401321  TAS2R30  UVRAG  KIF2C  MIA3  TMF1  TRIM44  CFC1  SLC25A40  CREBZF  SOX7  TRAK2  PIGC  NUP54  COQ10B  SLC25A5  COL7A1  AC068353.1  TAS2R43  HMBS  PPARGC1B  ZNF354C  ATP11A  PELI3  TMEM127  NXPE3  IL1RAP  C10orf97  DCLK1  ELOVL7  C1orf84  IFNE  IDI2  C5orf28  NHLRC2  FLJ33996  GIN1  SCP2  BLMH  KLF2  PFKFB2  CYTSB  RASSF5  EMX1  EIF2S3  POLDIP2  TSEN2  ADAM32  MURC  CAMSAP1L1  BTN2A2  COQ3  PCDHB13  PIKFYVE  FECH  ZBTB43  NUP35  ARL5B  KRI1  SLC29A1  GALE  PLEKHF1  ATP1A3  C20orf108  LRRC45  DAXX  SLC25A5P1  CTSA  SV2C  RCAN2  PCP4  PTX3  SLC15A4  LOC100288972  NPBWR2  MED11  ZNF496  C14orf138  CAST  SLC25A32  TMEM81  NARG1L  LOC100289861  SH3BP1  LPIN3  NBPF8  TAS2R20  MAP1LC3B  ATP1A4  14-Sep  NEFH  FBXL4  BCL10  ZNF232  IQUB  INSIG2  TANC1  NDUFC1  KLHL17  C14orf45  KLC2  HSPB9  EHBP1  GMCL1P1  LOC727803  ZNF791  MALAT1  KSR2  TTLL11  CEP350  IER5L  ARNT  C1orf109  LOC144438  PLEKHA1  AURKA  RBBP8  RPF1  ASAH2  CCDC64B  PREP  API5L1  ZFHX2  METAP2  ACYP2  C20orf7  YOD1  MYO5A  TARS  LOC440311  THG1L  C7orf63  GARNL1  SERAC1  DTX3  RPUSD2  PHOSPHO1  CHRM2  PLEKHA8  ATXN2L  RABEP2  C21orf57  2-Mar  MUTED  SFRS14  SLC7A8  NAB1  EDN3  FAM108A5P  SESN2  SLC45A1  HIGD1B  MKX  FOXQ1  DIP2B  WIPI1  CEBPG  MFSD9  LOC100132317  SV2A  PFN1  C11orf63  PPM1A  TDH  SNX29P2  PRDM1  ZNF3  IQCC  ABTB1  ZNF358  LOC730658  C17orf100  SUCLA2  HM13  POU3F3  USP8  HPSE  ST13  TMLHE  FES  SCAND3  SCRN2  MSL2  ZFP161  UGP2  POU3F1  RNF144B  LOC440180  HIST1H4E  DDHD1  MICA  PSMD6  DNAJC16  SBNO1  RNF220  FAM73B  TTC17  KCNS2  TRMU  LOC100132351  PKD2  CACNG8  FAM81A  RFX7  KDM5D  BICD2  USP31  SFXN4  THSD4  CDKN2A  HSPB2  SEMA4C  ZNHIT6  ZNF442  ZNF260  KLHL28  SLC12A2  GNPTG  LOC100289350  LOC100129104  PTCD1 | LRCH1  KIAA0802  GABRB2  C18orf25  MIR31HG  C6orf186  LOC100134173  ITPR1  IRS1  SIKE1  TCTEX1D1  STRADA  C19orf46  POU3F2  INTS8  SLC24A2  KBTBD2  TPP2  NAP1L2  ZNF284  KIAA1370  ABCC8  NR3C2  USP9Y  AP000926.2  SLC6A6  ZBTB34  TUBE1  OR2L3  ARHGEF11  ARL4C  ATP2A2  PTPRD  ANKRD29  DDX3Y  ESRRA  LINC00515  C20orf100  FKBP7  ELOVL4  BEND6  C2orf86  FHDC1  C17orf63  SNRNP35  C20orf200  RAD52  DDX11  SOST  TAF3  RELL2  FANCI  ARL2BP  RBAK  SYT2  IL6R  GIT2  DTWD2  SPAG4  ZNF273  CNNM2  SMC2  PI4K2A  CD99L2  PDSS1  TRAF3IP1  LYPLA1  CA7  CCDC39  ZDHHC2  SPAG1  DNAJC4  SUPT16H  TMEM69  TET3  RNF6  CHD6  REEP6  SNTG1  MYC  LTB  CORO7  ST3GAL5  CYB5R4  USP2  KCNH6  DPY19L2P2  TMEM204  MUDENG  MYO15A  CLCN2  STK38L  LOC100132364  FOXN3  TFAM  REPIN1  RAB3GAP2  CDYL  CAB39  LURAP1L  KIAA1797  CDON  ZNF654  EDNRA  GALNT12  KDM4C  LYSMD4  DIRAS1  FAR2  KCNMB3  RERG  PLEKHM2  KDELC1  FAM19A3  OR2M4  GJC1  HSPBAP1  SEPT7L  STX1B  AHI1  TRMT2B  NEAT1  POU6F2  OXSR1  CYorf15B  OGDHL  LOC653056  TAS2R50  ICT1  ATAD2  CRABP1  MYSM1  NAGPA  DCLRE1C  TAS2R10  SCN4B  STARD10  OSBPL5  ZNF641  STAU2  BMS1  CCNE1  ADO  KIAA1219  EID3  ADAMTS5  RAB11B  KCNIP3  LRRN3  MAST4  GVINP1  FBXL6  RUNDC3B  ZNF223  GUCA2B  LRRC38  FAM78A  PNPO  LOC389842  MLLT10  CCDC130  ABTB2  ETS2  SLC35A4  EPM2AIP1  MOSPD1  AC069154.2  SLC8A1  SNRPN  SASS6  PLEKHO1  TEAD4  AC090360.1  SNRK  DNAJC28  C14orf162  SLC3A1  CISD2  DND1  GSTK1  C5orf41  RNF160  SSH3  ANKRD20A5P  PLEKHM1  BOD1L  ANKRD20A8P  MED22  AGPAT3  FAM76B  KIF27  TFCP2L1  SNX21  ZHX1  C5orf53  LOC339290  IGF1R  LEO1  PRSS56  NAPA  ASAP2  SMOC2  NFIL3  ACPL2  IMPA1  NEFM  ISCA1  C11orf30  CDKN2C  ELL2  PAQR3  ERP44  ZADH2  HAUS5  EHD2  RANBP2  CCDC115  OXCT2  FLRT2  CHD1L  DNMBP  POGK  ATG2B  HCG4  ZNF280D  CKMT1A  LL22NC03-80A10.2  ASCL1  UHRF1BP1  GMEB1  C3orf58  TRPT1  PDZD7  NUDT5  C11orf24  PAPD4  ZNF155  ASPDH  ARSG  LARP4  GATAD2A  NET1  BCAS4  LAT  ABHD12  MPP5  TAF5L  PPP1R3D  FOSB  C8orf73  CEP97  ACADM  TSEN15  FMR1  CCDC104  CTSK  SLFN5  HYAL1  ZSCAN12  ANO5  DYRK4  AC087742.2  UCP3  WDR55  TMEM109  KRR1  ALG3  FAM33A  TMEM55A  RRP12  FBXW7  AC021534.2  RC3H1  BAK1  CNGB3  PHKA2  UTP15  ILK  NRIP1  DOT1L  ANKRD20A13P  SAFB2  COPA  KCNS3  TRDMT1  CNTLN  IFNAR2  NUDT8  AP003355.2  STX19  ICA1  NKAPP1  SENP6  MTMR15  ZNF33A  WDR19  GTF3C3  GRID2IP  UXS1  ZNF136  CFL2  FN3K  LOC283701  C9orf6  DHX57  ZNF367  KIF4A  ABT1  GOLGA8C  MYOZ2  C1orf113  MRAP2  ZBTB11  NAB2  OR4N2  PGK1  TMX2  MAK16  CCDC144A  LRRK2  CCDC132  C4orf3  SOCS4  RABL2A  PPAP2A  PGP  TRAPPC2P1  MYST1  FAM135B  TMPO  C12orf57  ASB8  FSTL4  MFSD2  DHX16  FOXL2  LENG1  PRDM8  EPHB2  CTBP1  ZNF596  PBRM1  NME7  CCDC122  ANKRD24  SYNE1  CCDC55  HSD11B1  PKM2  ADAL |
