# Supplementary material for: Bridging the Gap Between Morphometric Similarity Mapping and Gene Transcription in Alzheimer’s Disease
Source: Front Neurosci. 2021 Sep 29;15:731292. doi: 10.3389/fnins.2021.731292 (PMC8522649; doi:10.3389/fnins.2021.731292)
Supplement: Supplementary file 5 [file Table_3.DOCX]

**Table S3** Significant differentially expressed genes between AD and normal elders

| **Up-regulated differentially expressed genes** | | | | | |
| --- | --- | --- | --- | --- | --- |
| ITPKB  SLC35E1  ACACB  NSUN6  DTNA  PXDC1  NLN  C1orf61  MINDY1  MAFF  EZR  HIPK2  PRR11  MSI2  ANP32B  HIF3A  COL27A1  CDK13  FBXO32  ATP8B1  CFLAR  PDCD6  WNK1  LOC202181  KLF15  RFX4  PMP2  SNRNP48  CCDC152  CACNB2  EP400  ZFP36L1  PGF  CMBL  MKNK2  EPC1  HDAC7  TFEB  ARHGEF40  ERBIN  MALAT1  SLC12A7  SREK1IP1  GLUL  ID3  FYCO1  LIMD1  NFAT5  CECR2  FAT1  TAF15  POU3F2  KMT2E  MEIOC  EHMT1  AEBP1  RASSF4  ARHGEF26  LINC01949  GRAMD1C  SLC25A29  PALLD  SLC39A12  EMP1  SPPL2A  PAXIP1-AS2  SAMD4A  MEGF10  CPEB4  ZNF207  ZBTB20  UBN2  PECAM1  SOX9  LINC00926  RHPN2  PLOD2  ANLN  SSBP3-AS1  PCSK5  SRGAP1  KCNN3  RRBP1  IWS1  ACKR3  FXR1  MKLN1  SERPINB6  SAV1  FRMD4A  SILC1  EIF3C  NFATC2IP  KDM4B  MGST1  ZNF254  MIR570HG  IREB2  NR2F2  TRIM38  CUX1  GALNT2  ND6  DDIT4L  ANKRD10-IT1  ELK4  RGL3  PRMT2  RIPOR3  IGSF9B  ACSF2  NLRC5  FBXW4P1  BHLHE41  ADAMTSL4  IL1R1  B3GNT5  TCAP  UBE2I  ACBD5  TAGLN2  NXPE3  KIAA0754  TIMP3  ACVRL1  RBFOX3  CDC14A  GIMAP7  MORF4L2  TEAD2  VSIR  ZNF217  SLC4A4  UHRF1  PDGFA  TRIM33  RAPGEF3  C21orf91  EBF1 | SRRM2  TP53INP1  NFIA  ATF7IP  ADAM33  CXCR4  TNPO1  FDFT1  MAP4K4  ZC3H7B  THRA  GOLIM4  ZNF160  LPP  LIFR  PACS2  NOTCH2  ANKRD36  ZBED6  POLR1B  HBP1  ANKRD13A  SMC3  MED13L  HIP1R  PRR34-AS1  FBXW12  BBX  CASP6  GPER1  DDX59  SYMPK  UBXN2A  AMER2  SEMA3F  NFIC  USP34  RASEF  AGAP4  RAB11FIP3  DNASE1  PPP1R3D  ATP6V0E1  PCAT19  PPP1R16B  SERPINB1  MAGI1  PIK3C2A  FOXC1  ANKRD12  IL6ST  PRELP  ARHGEF10  TRA2A  NEK1  EGFR  RELL1  AK4  SLC26A6  MT2A  PARP10  SLF2  PARP9  ITGA6  PRDX2  GBP1  EXOSC1  CHD9  ATRX  BRD8  BAZ1A  SLC15A2  CNTRL  ZIC2  WWC1  NKX2-2  LLGL1  PTPN2  SPIRE2  TLE4  EMX2  KLF4  YBX3  AHNAK  CRB2  PDZD2  TGFBR3  SFSWAP  ZNF566  HEY2  ITGA8  SLC14A1  TPP1  GATA2-AS1  USP47  MIDN  CMTM7  PRDM16  CNTLN  LOC102724449  COL1A1  LINC02076  ENTR1  SOX6  HEG1  LINC00937  FAM185A  SKIL  LEF1  SMIM10  ABTB1  PEAR1  FBLN1  DPF3  DOCK5  MAP2K7  IGFBP7  ITIH5  HIVEP3  BASP1-AS1  GJA4  CASC4  LINC002481  C11orf96  PAPSS2  ZC3H10  INPP5D  ENTPD2  MTMR9LP  PEX26  BHMT2  ANKRD36B  AOC3  SSPN  GRK4  CD40  TDRD10  LSR  MED13 | FAM107B  NFKBIA  NOTCH2NLA  TBL1XR1  TNS1  TNFRSF10B  LRRFIP1  VCAN  NAV1  KCNE4  MT1M  GAS2L1  TSR1  TRAK1  SCAF11  ALMS1  UBE3A  FLCN  SEMA4C  NAV2  COL5A3  PTMA  CTTN  TCF3  SLC22A3  ID4  LRP10  YAP1  ANAPC16  IQCA1  PPP6R2  SCARNA17  ITGB5  NEDD1  MUC1  MAP4  SALL3  DNAJC1  C9orf64  RIOK3  SOX2  LINC01000  CARD6  SCAF4  RIN3  RBBP4  PTPRF  IL6R  UBE2D3  HCG18  KLF2  VEZF1  NKTR  MAP3K20  PDLIM4  TEAD1  CCNC  LOC100506282  PIEZO2  DOCK6  PPARA  DCHS1  AKAP8L  RAB13  MOBP  VASP  ANGPT2  BGN  MT1F  ELAVL3  PTN  PTEN  RBPMS  EIF3B  PIK3CB  SPAG9  SRGN  PRKD3  PPA2  CDS2  TMED10  CADM1  FAM120A  SCIN  IL13RA1  ITGB8  ABCA1  BACE2  TRIR  HSP90B1  TNFAIP2  HIGD1B  CBX3  NFATC2  RARRES2  DLC1  PTPN9  PDE5A  PKN3  MROH1  APLNR  MED26  P2RY14  ZSCAN30  HECTD4  SP100  ZNF713  HERC2P7  PAQR5  RDH5  CRTC3  SLCO1A2  ERAP2  ARHGEF28  XYLB  CABLES1  ITFG2  LOC389831  EPHA7  SLC16A9  CTNNA3  TFPI  HNRNPLL  LRRC69  NID1  ATG16L2  TARS3  CAVIN1  HLA-DRB4  ACAD10  PLCB4  NKAPP1  CUX2  WDFY2  LOC158434  KRTAP10-11  SOX4  CXCL2  TGFB1I1 | USP36  FOXO1  PTAR1  RNPC3  MZT2B  ZCCHC24  PALD1  TMEM106C  DBT  SASH1  ANKRD13D  TBL1X  CLDN15  IFNAR2  GRAMD2B  FAM181B  LATS2  PNISR  PLSCR4  PARP11  BMPR1B  JPX  NPAS3  TMEM106A  SMARCC1  LRP4  MRPS5  DLGAP4  DIP2A  SKI  PARD3  CWF19L2  DGKG  ANKRD9  SLC5A3  CSNK1A1  RBM33  LMNA  GFAP  CEP104  FNIP1  MT1G  RGS12  ATOH8  PTPRC  RAB20  TEP1  COL1A2  RHOJ  TNFRSF1A  LFNG  TRA2B  CHST6  ARRB1  WDR1  MT1X  PREX2  VAC14-AS1  SEC62  PMP22  BCAS1  LOC100190986  KTN1  ATP1A2  PON2  NFX1  ZNF609  LIMA1  SPEN  IFI16  GPAM  CYYR1  PAAF1  SLC52A3  SYTL4  ELF1  FAM107A  IRF1-AS1  HSDL2  LOC114224  MROH6  GALNT15  PTPN13  AFG3L2  ARHGEF10L  ITPK1-AS1  MECOM  TJP2  CPM  CPEB3 | DDIT4  LZTS2  KCNJ10  CREBBP  UACA  B2M  BDP1  ZFP36L2  TAF3  HVCN1  LUC7L3  SLC44A1  CEP295  YLPM1  AKAP10  QKI  ITPRIPL2  CSRNP1  PDLIM5  FMNL2  AGAP9  ACSS3  MICALL2  ADGRL4  KANK1  LOC101927166  SIK3  AJAP1  MFHAS1  STAG2  VTI1A  XAF1  KIF5B  KLC1  WWTR1  SAMHD1  NOMO3  OTUD7B  GAREM2  FGFR1  BCL6  FRYL  LAMB2  SLCO4A1  ADD1  TFAP2C  MRTFB  SEPTIN7P2  ZNF785  SOS2  PPP2R1B  FGR  HBS1L  GPATCH2L  SEPSECS-AS1  KCNJ16  PRPF38B  NOL3  CCDC9B  FLT1  SLC4A11  PRO1804  CDK6  ZFAND6  SCFD1  PELI1  FGFR3  SELENOP  ELAVL1  GLIS3  LPAR1  RUSC1-AS1  FRG1JP  TCF7L2  SLCO3A1  ANGPT1  LRRC32  MICAL3  SDC4  GJA1  CFI  LOC100996506  TNS3  SEC22C  RHOBTB3  C6orf163  AIF1L  SNX31  ESAM  NDE1 | PBXIP1  SLC7A2  SQSTM1  NACC2  ZNF721  SPPL3  ZFHX3  SORBS1  MSX1  ZMYM5  NFASC  ZIC1  LOC286437  MYO10  KIF1B  RORA  ZNF423  LOC101927699  TOB1  RHOQ  CBR4  RPL35A  KANK2  LOC100130987  NUCKS1  REST  TBX3  ITPR2  CDC42EP4  PPFIBP1  PIDD1  CHDH  NUMA1  FGFR2  RAB18  PPP1R12B  CHST11  TCFL5  BAG3  UBE2Z  ZDHHC21  C19orf18  PLXNB1  SGSM2  LEAP2  TOP1  CSPG4  FOXD1  MRGPRF  GBP2  BMPR1A  STON2  CD44  SAT1  LMO2  PHYHD1  PPP2R3C  MAPKAPK3  RESF1  EHD2  SLC25A13  ASAP1-IT2  DAPK1-IT1  DIO3OS  RAPH1  SOCS3  GRTP1  SLC25A18  CDC42BPB  GKAP1  POGK  EPS15L1  PDK4  CEP89  NCS1  MIB1  SLC11A2  IGF2BP2  RBM5  HES1  SLC2A1  SMAD6  HGF  SREK1  VAPA  GPR4  TCIRG1  TBC1D16  CXCL1  SCAMP1 |

| **Down-regulated differentially expressed genes** | | | | | |
| --- | --- | --- | --- | --- | --- |
| ATP5F1C  TUBB  TUBB4B  PSMB3  ATP5F1B  TUBB3  YWHAZ  SNCA  PCYOX1L  SNAP25  RAB2A  PSMB2  DZIP3  DMAC1  NME1  CD200  PSMB7  P4HTM  YWHAB  SARS1  INA  IDH3G  ATP6V1G2  RRAGA  FAM162A  UCHL1  NDUFA7  SLC35B1  EGR1  SV2B  POP4  PTPN3  PPP1R7  RPL15  CRYM  BEND5  CALM1  COX5B  CADPS  GOT1  DPP6  EIF2B3  C12orf10  TRIM37  TAFA1  RNFT2  PDK3  CDK7  SYT13  IARS1  KIFAP3  ATP8A2  UBLCP1  TM2D2  NMRAL1  PCMT1  KIAA0513  KDM1A  TAGLN3  PSMA1  MAL2  MRPS9  NIF3L1  LYRM9  SNU13  AAK1  HIKESHI  LMO4  TPM3  NDUFA4  C11orf1  DNAJA4  ATP5IF1  GPHN  CAMK1G  FGF12  NCALD  SLC1A6  APLP2  PSMD12  SUSD4  MPV17  GSS  ENC1  SRPK2  TTC19  MECR  MAST3  TUB  C9orf72  TMX4  PHF20L1  HSPA12A  SNRPA1  MRTO4  SCN3B  ACTB  ALAS1  SYN2  ODF2  LETMD1  HUNK  TBC1D19  POLR3A  TM7SF2  CDK5  RPA3  HMGCS1  RPP40  CIRBP  FAM71E1  ST6GALNAC5  SEMA6D  PSMD8  DNM1  GPR158  NUDT18  HOPX  SLC17A7  LNPK  CCT7  TOMM22  YTHDF2  COMMD4  SERPINF1  CCNA1  NDUFV2  PFDN1  TRUB2  NFU1  MAEL  MBD4  B3GNTL1  BSN  COPS8  ANKS1B  ANKRD39  ATOH7  YJEFN3  U2AF1  DUS4L  MRPL17  GDE1  HLF  CITED1  CLPTM1L  NEUROD6  TTLL1  ARHGAP26  OAZ1  GADD45GIP1  ARL6  ERC2  NEFL  ATG2B  EFCAB7  EPB41L4B  MRPS12  DCAF6  SYT4  CXorf40A  API5  SRSF3  SMYD5  LOC100126784  PSMB1  TCEAL7  VSTM2A  MTFR1L  ANP32E  FRMPD4  SCO1  PCID2  SUSD1  FXYD6  SNCB  HTR2A  HLA-DQB1  DACH2  GAP43  TYRP1  TMEM9B  PPIEL  PLK4  SUN1  PKIB  MET  GAD1  MKX  NR4A3  NEFH  EDRF1-DT  EPHA3  MED6  PCDH8  MSC-AS1  RARRES1  IL13RA2  TPK1  GOLT1A  CREG2  COPE  TMEM232  TLL2  ZNF165  SATB2-AS1  C12orf29  UBE2M  TSPYL1  IMMP1L  GALNT12  LINC00467  PYCR3  C11orf80  CDH8  LINC00643 | ZNF415  MLLT11  LGALS8  RUFY3  ME3  TUBA1C  SRP54  ERLEC1  SAMM50  DDX10  GPI  SRD5A3  UTP4  TUBA1B  TMEM97  PSMD1  ANXA7  SF3B5  UBE2V2  EIF3H  NDUFAB1  SLC25A4  SLIRP  THUMPD3  FIG4  UBE2N  RTN3  MICU1  SST  NMNAT2  ACTR1A  ATP6V1B2  CMSS1  ATP6V1E1  RAP1GDS1  IMMT  NDUFS7  PPP2R2D  BCAS2  ANAPC13  EPM2AIP1  SAP18  IDH3A  CUL1  LAMP5  KBTBD11  NDUFV1  NCBP2AS2  TRAPPC12  PPIH  SIPA1L1  HIVEP2  C2orf80  SLC25A12  LRRC49  MDH2  FH  GLRB  SVOP  FKBP1B  JAZF1-AS1  BEX4  CHCHD2  STAT4  ORC5  RGS4  CYFIP2  SEZ6L2  NDUFB5  GRIN1  BEX5  ICA1  ACTR10  CARTPT  GNG3  GLOD4  MKRN1  NQO2  KPNA2  ROMO1  PITRM1  ARPC1A  SPATA7  CLTC  RIMBP2  GPATCH2  VPS53  CFAP69  MRPL37  SLC25A11  RASGRF1  TMEM191A  EIF5  VDAC1  CMAS  GATB  PAFAH1B1  ACTN2  HYLS1  DHX30  TIMM17A  MRPL9  MAPK9  DDX50  SIRT3  VPS33B  ABT1  PITHD1  TRMT10C  RAB3C  SURF2  CASD1  CLTB  PDHB  NDUFA13  PPEF1  PCLO  MRPS28  PPP1R14C  LARP1B  MAP7D2  LAMB1  ZDHHC4  TM2D3  ELMO1  UCHL5  CCNDBP1  NDUFS3  ALDOA  DNTTIP1  ATP5PO  DYNLL1  TMEM178A  MED8  RUVBL2  DHX15  NELL1  BTN2A1  SLC7A14  EIF3M  ARHGAP32  PPIB  CLIP3  PCSK2  TCP11L1  GRIN2A  CLCN4  FSD1  PRMT5  ZNF365  ZNF222  RPA1  EMC1  MCM4  CLBA1  SRD5A1  SCOC  PODXL2  DOCK3  NUP42  EXOSC4  VPS28  NECAB1  OLFM3  MRPL4  WDR46  CYC1  MRPS30  ABCC12  PRKAG2-AS1  ROBO2  WASL  KCNJ6  NWD2  PLA2G4A  DTNBP1  RHEBL1  CFL1  RSPO2  HNRNPL  PVALB  RPS4Y1  SLC24A3  HTR2C  LINC00507 | EMC4  COPS4  METTL3  RAD51C  RTL8C  MPP1  CDC42  ENO2  RTN1  UQCRC2  COPS3  TSG101  LARP4B  NIT2  ASAH2B  DYNC1I1  CDC37  CALY  CHRM1  CD2BP2  OCIAD1  SULT4A1  EIF3K  CRMP1  ANXA6  NRXN3  TPI1  NDFIP2  CHGB  CZIB  SELENOI  NRN1  SDHB  AP3M2  ADAM23  YWHAH  ITFG1  MTX2  SDR16C5  PGM2L1  PARP2  UQCRFS1  TAF9B  PHF24  ATP6V1F  PAIP2  DYRK4  EIF3D  PEBP1  MICAL2  TBC1D9  GLS2  FABP3  CSRNP2  MIF  PSMG1  UNC13A  TARBP1  PAK1  MKKS  SGPP2  RRP36  GNB5  MED14  RNF175  CUL3  MAPK1  SPCS1  HSP90AB1  MIR7-3HG  SVIP  SERTM1  EXTL2  PRKCZ  EDF1  GLS  UQCRC1  CCK  REPS2  DNM3  BLVRB  PHB  CDS1  LRRC8B  COX7B  CCT2  PLCL2  TOMM20  ATP6V0C  SYT1  PNMA8A  THY1  EIF3G  SLC39A10  PRKCB  WIPF3  PPFIA2  PSMA3  RTRAF  FAM149B1  TXN  MIR124-2HG  ZNF57  ATL1  AP3B2  CALB1  MALSU1  CACNG3  GLO1  PLK2  NTPCR  C1orf216  ATR  LANCL1  RALYL  RAB3B  SPHKAP  SLC25A3  ARPP19  SPTAN1  GALNT17  AZI2  SPIN3  TM9SF2  STAR  DNAJC6  GYG1  LSM7  AQP11  TLN2  AMZ2P1  EXOSC5  C8orf88  FCRLB  FAM86B3P  ZNF488  GPR22  LINC00665  CORO1A  SLIT3  PHF14  LRPAP1  SNAP91  KCNV1  GABRD  TMEM59L  PRKAG1  KIAA1324  GDA  MLX  SLITRK5  GMDS  SLC8A1  EIF5A2  KHDC1  LINC00460  LRRC75A  RPF1  NAP1L2  DGKB  THEM6  SYCE1  IDNK  MLIP  AAGAB  MRPL20  AP4S1  CHAF1B  KCNQ5  FBXW7  MTSS1  HECW1  PSME3  GAD2 | SCN2B  ACOT7  PPME1  LRPPRC  ZNF204P  FHL2  DHCR24  JAZF1  GABBR1  STMN2  MAP2K4  GABRG2  DHRS7B  PTPRN2  MTPAP  GOT2  PFKM  ATP5MPL  ATP2B2  LRFN5  BCCIP  PTP4A1  NSF  MAP6  KCNIP4  PREPL  BRWD1  ATP6V1H  TRAM1L1  MRPS23  HINT1  ANAPC5  LDHA  IMPDH2  SCCPDH  STX8  PRDM2  NAP1L5  DYNC1H1  ATRNL1  FAR2  GHITM  TMEM178B  SCN8A  SNX14  NDUFA1  CCKBR  TIMM50  SGIP1  CCT4  MAP2K5  KMT5B  EML6  NVL  MRPL15  SEPTIN7  AGAP2  HMOX2  PPP2CA  ZWINT  GAPDH  PGK1  GABARAPL1  SNAPC5  RRAGB  TMEM50A  TMEM163  WASHC3  ATP1A1  IP6K2  COMMD9  FARSA  RAB1A  NSG1  SLIT2  NAV3  SNX3  GTF2B  GABRA1  ANKRD36BP2  CAPNS1  SLC16A14  ATP6V1D  FHOD3  CLPP  UGP2  VPS50  CEP41  TTC9C  ANKMY2  EXOG  SLC27A4  LMBR1  MAGED2  SCG5  SSU72  PSMC4  TMEM70  OXCT1  MEF2C  ASMTL  FADS3  LAMTOR2  CDH18  IK  DNAH6  HSPB3  SLC25A14  SYNJ1  DNAH9  PCSK1  NSDHL  RIIAD1  MOCS2  SYBU  PNMA8B  UBE2QL1  NPTX2  STEAP2  YKT6  DYNC2LI1  ANK3  SOSTDC1  USP39  SCN2A  UBE2T  KIF3A  RETREG1  ANKRD34C  GTF3C1  CERNA1  COQ3  PLD3  RIT2  STAMBPL1  RBFOX2  C11orf97  NUDT6  MAPK11  TRIM36  DNAJC12  PRC1  TMEM18  FGF14  PTPRT  BHLHE22  SNAP29  LRRTM1  SLC6A15  PAK3  PARM1  DMXL2  LOC729870  BDNF  DUSP4  XRN2  NETO1  GIT2  CPNE6  ADAL  ENDOG  RANBP10  GNG2  ADAT2  CAMK2B  KCNS2  TMEM165  IGF1  VXN  COG7  ZNF416  METTL21A  TMOD1 | KRT222  MDH1  RGS7  PLPBP  TUBA4A  WARS1  REEP1  EMC3  MOB4  VPS35  TBC1D7  AP2S1  FUCA1  EPB41L3  EEF1A2  MRPL30  BLVRA  COPS5  NHP2  NECAP1  FIBP  CAPRIN2  UBE2D2  EEF1E1  NUDT2  NDRG4  ATP6V1A  PSMD14  COA1  APOO  CA10  MAGED1  GNAS  ORC3  TSPYL5  RTN4IP1  RAB11A  NSG2  CNOT10  KIZ  ARHGEF9  ZDHHC23  ATOX1  ASNS  SKP1  CHP1  SNX10  WDR61  CAMTA1  SUMO1  TBC1D30  MAP2K1  PFN2  TMEM121B  LOC100507557  INPP4A  TMEM126A  SUB1  RASA1  SPOCK1  WDR7  SERINC3  CXorf40B  RBFOX1  DDX41  VAMP7  GLRX  MGST3  C3orf14  ATP2B3  HACL1  WDR70  SIVA1  ST8SIA5  HAGH  TOMM5  DCLK1  RNF181  ACTL6B  KIF9  THYN1  PRMT8  EMC9  ERCC1  RITA1  DLGAP1  AK5  CELF1  OCIAD2  NDUFB10  DHX36  DDX1  HMGCR  NRGN  EID1  ATP5F1A  FAM201A  EPHA4  ZC3H14  MSH2  NDRG3  PLCXD3  PPP3CA  CDKL1  PTRH2  NDUFA11  GOSR2  AP2M1  PDS5A  LRMP  DMAP1  PRR3  GABRA5  ZNRD2  XRCC6  ZNF780A  SPATS2  UBL7  SLC25A5  GRIA1  SCG2  TSC22D1-AS1  ZFPM2  AHNAK2  TERF2IP  TMEFF2  SH3GL2  PPP3CB  DTNB  DDX28  ARHGEF7  ARMCX4  BNIP1  KCTD4  DRP2  PACSIN1  CACNA2D3  NAPB  PDE1A  ARHGDIA  NEU1  ARF3  ZMAT4  FAXC  NRBF2  OSTF1  NRG1  ATP6V0A1  AK9  DMAC2  PSMC3  PNMA2  MRPL43  CHURC1  NAA80  OPN3  B3GALNT1  ARG2  UBE2S  PSMA7  CDH12  STRBP  POLH  CMTM1  ZNF200  RAB27B  SLC25A27  PGD  WBP11  NXPH2  FGF13  CSTF1 | GUCY1B1  MFSD4A  POLDIP2  HS6ST3  AKIRIN2  AMPH  ATP2A2  VSNL1  ATXN10  MRPL47  CHCHD6  SLC9A6  CDC40  TRAPPC2L  HMGCLL1  PSMA5  C1QTNF4  PSMB4  RWDD2A  MCTS1  UQCR10  RWDD2B  POLR3C  ZCCHC17  GABBR2  ITGB1BP1  DGUOK  JAK1  GRPEL1  TOMM70  ATP1A3  ADCYAP1  SMYD3  LOC728392  TUSC3  DPCD  CSE1L  PREP  ACP1  CSNK2B  LARGE1  KARS1  ERICH3  MAD2L1BP  IFT122  SSX2IP  CLEC2L  SMARCA4  ATP6V0B  ATP6AP1  RPH3A  RAN  COG1  GPRASP1  EBP  GABRA4  NELL2  SLC25A46  COMMD1  LMBRD2  PIAS2  EPDR1  TMEM35A  DPY30  TSPAN7  CNKSR2  SYNE1  PGAP4  DDX25  ATP6V1C1  ADARB1  NDUFAF1  PRMT7  CCT5  MMADHC  AP1S2  PPM1E  PTPRO  NDUFB8  HPRT1  NAA20  ZCCHC12  ZBBX  VDAC3  C14orf119  EXOC8  LY86-AS1  PSMD10  LOC100506563  PSMD11  SLC35G1  NRXN1  ZNF554  STXBP1  DLD  RUVBL1  NRIP3  EFNB3  PCP4  DHPS  ANO4  SEH1L  RABGGTB  IAH1  SUCLA2  SEPHS1  SYAP1  ATG7  TSPAN3  SOD1  IGFBPL1  SERPINI1  PI4KA  ATP5MC3  SYN1  FAHD2A  TTBK2  ADK  DDX6  STMN1  ECHDC1  JPT1  AMER3  RMND1  SLC25A23  LRRC4  VAMP1  PTGR1  NAPA  CRH  MYT1L  LDB2  PPFIA4  TSTA3  SLC32A1  RASGRF2  TAC1  LMLN  DHX35  RPRD1A  RBP4  NEFM  GRIA4  PSMC5  POR  TMEM230  ZNF385B  ZNF215  TDRKH  SLF1  SLC30A3  PKM  KCNA1  DMAC2L  SELENOO  PCDHAC2  MAPKAPK5-AS1  WDR37  ENOSF1  TMEM200A  PIGL  RRP7A  SYTL5  ZNF518B  SRR  EIF5A  ANO3  IL1RAPL1  STYK1  TRHDE  MAP3K7  DNPH1 |
