# Supplementary material for: Bridging the Gap Between Morphometric Similarity Mapping and Gene Transcription in Alzheimer’s Disease
Source: Front Neurosci. 2021 Sep 29;15:731292. doi: 10.3389/fnins.2021.731292 (PMC8522649; doi:10.3389/fnins.2021.731292)
Supplement: Supplementary file 6 [file Table_4.DOCX]

**Table S4** Cell-type-enriched gene lists for each type of cortical cells

| **Astrocytes** | | | | |
| --- | --- | --- | --- | --- |
| ITPKB  SLC35E1  ACACB  NSUN6  DTNA  PXDC1  NLN  C1orf61  MINDY1  MAFF  EZR  HIPK2  PRR11  MSI2  ANP32B  HIF3A  COL27A1  CDK13  FBXO32  ATP8B1  CFLAR  PDCD6  WNK1  LOC202181  KLF15  RFX4  PMP2  SNRNP48  CCDC152  CACNB2  EP400  ZFP36L1  PGF  CMBL  MKNK2  EPC1  HDAC7  TFEB  ARHGEF40  ERBIN  MALAT1  SRRM2SPPL3  ZFHX3  SORBS1  MSX1  ZMYM5  NFASC  ZIC1  LOC286437  MYO10  KIF1B  RORA  ZNF423  LOC101927699  TOB1  RHOQ  CBR4  RPL35A  KANK2  LOC100130987  NUCKS1  REST  TBX3  ITPR2  CDC42EP4  PPFIBP1  PIDD1  CHDH  NUMA1  FGFR2  RAB18  PPP1R12B  CHST11  TCFL5  BAG3  UBE2Z  ZDHHC21  SLC12A7  SREK1IP1  GLUL  ID3  FYCO1  LIMD1  NFAT5  CECR2  HSDL2  LOC114224  MROH6  GALNT15  PTPN13  AFG3L2  ARHGEF10L  ITPK1-AS1  MECOM  TJP2  CPM  CPEB3  FRYL  LAMB2  SLCO4A1  ADD1  TFAP2C  MRTFB  SEPTIN7P2  ZNF785  SOS2  PPP2R1B  FGR  HBS1L  GPATCH2L  SEPSECS-AS1  KCNJ16  PRPF38B  NOL3  CCDC9B  FLT1  SLC4A11  PRO1804  CDK6  ZFAND6  SCFD1  PELI1  FGFR3  SELENOP  ELAVL1  GLIS3  LPAR1  RUSC1-AS1  FRG1JP  P2RY14  ZSCAN30  HECTD4  SP100  ZNF713  HERC2P7  PAQR5  RDH5  CRTC3  SLCO1A2  ERAP2  ARHGEF28 | TP53INP1  NFIA  ATF7IP  ADAM33  CXCR4  TNPO1  FDFT1  MAP4K4  ZC3H7B  THRA  GOLIM4  ZNF160  LPP  LIFR  PACS2  NOTCH2  ANKRD36  ZBED6  POLR1B  HBP1  ANKRD13A  SMC3  MED13L  HIP1R  PRR34-AS1  FBXW12  BBX  CASP6  GPER1  DDX59  SYMPK  UBXN2A  AMER2  SEMA3F  NFIC  USP34  RASEF  AGAP4  RAB11FIP3  DNASE1  FAM107B  NFKBIA  FAT1  TAF15  POU3F2  KMT2E  MEIOC  EHMT1  AEBP1  RASSF4  ARHGEF26  LINC01949  GRAMD1C  SLC25A29  PALLD  SLC39A12  EMP1  SPPL2A  PAXIP1-AS2  SAMD4A  MEGF10  CPEB4  ZNF207  ZBTB20  UBN2  PECAM1  SOX9  LINC00926  RHPN2  PLOD2  ANLN  SSBP3-AS1  PCSK5  SRGAP1  KCNN3  RRBP1  IWS1  ACKR3  FXR1  MKLN1  SERPINB6  SAV1  FRMD4A  PPP1R3D  ATP6V0E1  PCAT19  TCF7L2  SLCO3A1  ANGPT1  LRRC32  MICAL3  SDC4  GJA1  CFI  LOC100996506  TNS3  SEC22C  RHOBTB3  C6orf163  AIF1L  SNX31  ESAM  NDE1  C19orf18  PLXNB1  SGSM2  LEAP2  TOP1  CSPG4  FOXD1  MRGPRF  GBP2  BMPR1A  STON2  CD44  SAT1  LMO2  PHYHD1  PPP2R3C  MAPKAPK3  RESF1  EHD2  SLC25A13  ASAP1-IT2  DAPK1-IT1  DIO3OS  RAPH1  SOCS3  GRTP1  SLC25A18  XYLB  CABLES1  ITFG2  LOC389831  EPHA7  SLC16A9  CTNNA3  TFPI  HNRNPLL  LRRC69  NID1  ATG16L2 | NOTCH2NLA  TBL1XR1  TNS1  TNFRSF10B  LRRFIP1  VCAN  NAV1  KCNE4  MT1M  GAS2L1  TSR1  TRAK1  SCAF11  ALMS1  UBE3A  FLCN  SEMA4C  NAV2  COL5A3  PTMA  CTTN  TCF3  SLC22A3  ID4  LRP10  YAP1  ANAPC16  IQCA1  PPP6R2  SCARNA17  ITGB5  NEDD1  MUC1  MAP4  SALL3  DNAJC1  C9orf64  RIOK3  SOX2  USP36  FOXO1  PTAR1  PPP1R16B  SERPINB1  MAGI1  PIK3C2A  FOXC1  ANKRD12  IL6ST  PRELP  ARHGEF10  TRA2A  NEK1  EGFR  RELL1  AK4  SLC26A6  MT2A  PARP10  SLF2  PARP9  ITGA6  PRDX2  GBP1  EXOSC1  CHD9  ATRX  BRD8  BAZ1A  SLC15A2  CNTRL  ZIC2  WWC1  NKX2-2  LLGL1  PTPN2  SPIRE2  TLE4  EMX2  KLF4  YBX3  AHNAK  CRB2  PDZD2  TGFBR3  SFSWAP  CDC42BPB  GKAP1  POGK  EPS15L1  PDK4  CEP89  NCS1  MIB1  SLC11A2  IGF2BP2  RBM5  HES1  SLC2A1  SMAD6  HGF  SREK1  VAPA  GPR4  TCIRG1  TBC1D16  CXCL1  SCAMP1  SILC1  EIF3C  NFATC2IP  KDM4B  MGST1  ZNF254  MIR570HG  IREB2  NR2F2  TRIM38  CUX1  GALNT2  ND6  DDIT4L  ANKRD10-IT1  ELK4  RGL3  PRMT2  RIPOR3  IGSF9B  ACSF2  NLRC5  TARS3  CAVIN1  HLA-DRB4  ACAD10  PLCB4  NKAPP1  CUX2  WDFY2  LOC158434  KRTAP10-11  SOX4  CXCL2 | RNPC3  MZT2B  ZCCHC24  PALD1  TMEM106C  DBT  SASH1  ANKRD13D  TBL1X  CLDN15  IFNAR2  GRAMD2B  FAM181B  LATS2  PNISR  PLSCR4  PARP11  BMPR1B  JPX  NPAS3  TMEM106A  SMARCC1  LRP4  MRPS5  DLGAP4  DIP2A  SKI  PARD3  CWF19L2  DGKG  ANKRD9  SLC5A3  CSNK1A1  RBM33  LMNA  GFAP  CEP104  FNIP1  DDIT4  LZTS2  KCNJ10  CREBBP  ZNF566  HEY2  LINC01000  CARD6  SCAF4  RIN3  RBBP4  PTPRF  IL6R  UBE2D3  HCG18  KLF2  VEZF1  NKTR  MAP3K20  PDLIM4  TEAD1  CCNC  LOC100506282  PIEZO2  DOCK6  PPARA  DCHS1  AKAP8L  RAB13  MOBP  VASP  ANGPT2  BGN  MT1F  ELAVL3  PTN  PTEN  RBPMS  EIF3B  PIK3CB  SPAG9  SRGN  PRKD3  PPA2  CDS2  TMED10  CADM1  FAM120A  FBXW4P1  BHLHE41  ADAMTSL4  IL1R1  B3GNT5  TCAP  UBE2I  ACBD5  TAGLN2  NXPE3  KIAA0754  TIMP3  ACVRL1  RBFOX3  CDC14A  GIMAP7  MORF4L2  TEAD2  VSIR  ZNF217  SLC4A4  UHRF1  PDGFA  TRIM33  RAPGEF3  C21orf91  EBF1  ITGA8  SLC14A1  TPP1  GATA2-AS1  USP47  MIDN  CMTM7  PRDM16  CNTLN  LOC102724449  COL1A1  LINC02076  ENTR1  SOX6  HEG1  LINC00937  FAM185A  TGFB1I1  FLI1  CCN4  ITGB2-AS1  OSBP2  RGS1  CD163  FN1  PCSK7  MYH11  LUM  HERC2P3 | UACA  B2M  BDP1  ZFP36L2  TAF3  HVCN1  LUC7L3  SLC44A1  CEP295  YLPM1  AKAP10  QKI  ITPRIPL2  CSRNP1  PDLIM5  FMNL2  AGAP9  ACSS3  MICALL2  ADGRL4  KANK1  LOC101927166  SIK3  AJAP1  MFHAS1  STAG2  VTI1A  XAF1  KIF5B  KLC1  WWTR1  SAMHD1  NOMO3  OTUD7B  GAREM2  FGFR1  BCL6  PBXIP1  SLC7A2  SQSTM1  NACC2  ZNF721  SCIN  IL13RA1  ITGB8  ABCA1  BACE2  TRIR  HSP90B1  MT1G  RGS12  ATOH8  PTPRC  RAB20  TEP1  COL1A2  RHOJ  TNFRSF1A  LFNG  TRA2B  CHST6  ARRB1  WDR1  MT1X  PREX2  VAC14-AS1  SEC62  PMP22  BCAS1  LOC100190986  KTN1  ATP1A2  PON2  NFX1  ZNF609  LIMA1  SPEN  IFI16  GPAM  CYYR1  PAAF1  SLC52A3  SYTL4  ELF1  FAM107A  IRF1-AS1  SKIL  LEF1  SMIM10  ABTB1  PEAR1  FBLN1  DPF3  DOCK5  MAP2K7  IGFBP7  ITIH5  HIVEP3  BASP1-AS1  GJA4  CASC4  LINC002481  C11orf96  PAPSS2  ZC3H10  INPP5D  ENTPD2  MTMR9LP  PEX26  BHMT2  ANKRD36B  AOC3  SSPN  GRK4  CD40  TDRD10  LSR  MED13  TNFAIP2  HIGD1B  CBX3  NFATC2  RARRES2  DLC1  PTPN9  PDE5A  PKN3  MROH1  APLNR  MED26  HIPK1-AS1  SERPINA3  VAMP5  SDC3  TRIM56  MYOF  TM4SF18  LOC100507477  TPM2  XIST |

| **Neurons** | | | | | |
| --- | --- | --- | --- | --- | --- |
| 11-Mar  ABCA4  ABCB1  ABCC3  ABI3BP  ABRA  ABTB1  ACE  ACHE  ACOT7  ACRV1  ACSBG2  ACTA1  ACTA2  ACTC1  ACTL6B  ADAM11  ADAM15  ADAM21  ADAMTS16  ADAMTS19  ADAMTS3  ADAMTS6  ADAMTS9-AS2  ADCK5  ADCY1  ADCYAP1  ADD2  ADM2  ADRA1B  ADRA2A  AFAP1-AS1  AFF2  AGAP2  AGTR1  AIFM2  AIPL1  AKR1C1  AKR1E2  AKR7A2P1  AKR7L  ALG1L2  CORO6  CORT  COX4I2  COX6A2  CPA1  CPLX3  CPNE4  CPNE7  CPO  CRHR1  CRHR2  CROCC  CRYBB3  CRYGA  CRYGC  CRYGD  CRYM  CRYM-AS1  CSPG4  CST4  CXADRP3  CXCL6  CYB561  CYLC2  CYP1B1  CYP24A1  CYP27B1  CYP27C1  CYP2A6  CYP2C8  CYP2D6  CYP2E1  CYP2G1P  CYP4F2  CYP4X1  CYP4Z2P  CYYR1  DAB1  DACH2  DBIL5P  DBIL5P2  DCAF12L2  DCD  DCLRE1A  GRIN1  GRIN2D  GRIP1  GRK6  GRK7  GRM2  GRM6  GRM8  GRPR  GSTA7P  GSX2  GTSE1  GUCA1C  GULP1  GUSBP2  GYG2  GYG2P1  GYPB  GYPE  H19  H2AFY2  HAPLN1  HAPLN4  HAR1A  HAS1  HAS3  HBQ1  HCAR3  HCRTR1  HELLS  HGFAC  HIGD1B  HIGD1C  HIST1H1D  HIST1H2AI  HIST1H2AK  HIST1H2AM  HIST1H2BF  HIST1H2BH  HIST1H2BJ  HIST1H2BM  HIST1H3B  HIST1H3C  HIST1H3H  METTL21C  MEX3B  MFNG  MFSD9  MICAL2  MIMT1  MIP  MIR106B  MIR1185-2  MIR1-2  MIR1203  MIR1204  MIR1208  MIR1252  MIR1256  MIR1272  MIR1275  MIR1276  MIR128-1  MIR1284  MIR129-1  MIR1293  MIR130A  MIR133A2  MIR133B  MIR137HG  MIR138-1  MIR140  MIR141  MIR146A  MIR147A  MIR1537  MIR181A2  MIR1827  MIR184  MIR188  MIR1911  MIR1912  MIR194-2  MIR2053  MIR218-1  MIR23B  MIR298  MIR29A  NEGR1-IT1  NEK10  NEUROD1  NEUROD2  NEUROD4  NEUROD6  NGB  NGEF  NGF  NHLH1  NLRP4  NMU  NNMT  NOS2  NOX1  NOX3  NPAS4  NPFF  NPPB  NPPC  NPR3  NPTX1  NPY5R  NPY6R  NR1H4  NR1I3  NR6A1  NRG1  NRG4  NRGN  NRK  NRSN1  NSUN7  NT5C1A  NT5DC2  NTF3  NTNG1  NTSR1  NUDT10  NUDT11  NUDT18  NUDT6  NUP210P1  NUP62CL  RPS6KA6  RRAS  RSAD2  RSC1A1  RSPO2  RSPO3  RTBDN  RTN4RL1  RTP1  RUNDC3B  S100A3  S100A4  S100P  SAA4  SALL4  SAMD10  SAMD12-AS1  SAMD5  SCARNA1  SCARNA10  SCARNA11  SCARNA12  SCARNA18  SCARNA21  SCARNA5  SCARNA6  SCARNA8  SCGB1A1  SCGB2A2  SCGN  SCN2A  SCN5A  SCO2  SCTR  SDR16C5  SELP  SEMA5B  SENP3  SERTM1  SEZ6  SFTA3  SH2D3C  SH2D5  SH3BP5  TAAR5  TAC3  TACC3  TACR1  TAF4B  TAS2R50  TAS2R7  TAS2R8  TBC1D30  TBX15  TBXA2R  TC2N  TCAM1P  TCEA3  TCEAL6  TCERG1L  TCP11  TCTEX1D1  TDO2  TDRD1  TDRD5  TDRG1  TEDDM1  TEKT5  TELO2  TERT  TESC  TEX12  TEX21P  TFPT  TFR2  TG  TGM4  THBS1 | ALS2CL  AMBP  AMH  AMHR2  AMIGO1  AMPH  ANGPTL7  ANKLE1  ANKRD2  ANKRD20A11P  ANKRD30B  ANKRD30BL  ANKRD34B  ANKRD34C  ANKRD53  ANO1  ANXA1  ANXA13  APEX2  APOL5  ARAP3  ARG1  ARHGAP10  ARHGAP11A  ARHGAP11B  ARHGAP19-SLIT1  ARHGAP28  ARHGAP36  ARHGAP39  ARHGAP4  ARHGEF15  ARHGEF25  ARID3C  ARL15  ARMC3  ARMCX5-GPRASP2  ARMS2  ARR3  ASB12  ASB5  DCST1  DCST2  DDX43  DEAF1  DEFB1  DEFB128  DEFB134  DEFB136  DEGS2  DERL3  DET1  DGCR10  DHRS4L1  DIRAS2  DISP2  DKK2  DKK4  DLEC1  DLGAP2  DLK2  DLL4  DLX2  DLX5  DLX6  DLX6-AS1  DMKN  DNAAF1  DNAH3  DNAJA1P5  DNAJC5B  DNAJC5G  DNASE1L1  DNASE1L2  DNASE2B  DNM1P35  DOK4  DPH1  DPP4  DPRXP4  DPT  DPY19L2P4  DRD1  DRD2  DSCAM-AS1  HIST1H3J  HIST1H4D  HIST1H4F  HIST1H4K  HIST4H4  HK3  HLA-H  HMGB3P1  HMGCLL1  HMGN2P46  HOMER2  HORMAD1  HORMAD2  HPCA  HPS6  HPX  HPYR1  HRAS  HRASLS  HS3ST2  HS6ST3  HSD11B2  HSD3B1  HSD3BP4  HSF5  HSPB3  HSPB9  HTR1A  HTR1E  HTR3A  HTR3B  HTR3C  HTR4  HTR5A  HTR7  HTRA2  HUNK  HYAL2  HYAL3  IDO1  IER5L  IFIT1B  IFITM1  IFNA22P  MIR300  MIR30B  MIR30C2  MIR30D  MIR3123  MIR3126  MIR3146  MIR3153  MIR3156-1  MIR3156-2  MIR3165  MIR3167  MIR3186  MIR3190  MIR3198-1  MIR320B2  MIR320C1  MIR323A  MIR323B  MIR326  MIR329-2  MIR335  MIR3609  MIR3612  MIR3675  MIR3677  MIR3680-1  MIR3692  MIR373  MIR374A  MIR375  MIR376A2  MIR377  MIR378E  MIR379  MIR380  MIR382  MIR383  MIR3922  MIR3939  MIR3941  MIR3943  MIR3945  MIR4258  NXNL2  NXPH1  NXPH2  NXPH3  NYNRIN  OBSCN  OCA2  OCM  ODF3L2  OLFM4  OPN1SW  OPN3  OPN5  OPRK1  OPRL1  OPRM1  OR10A4  OR10A5  OR10AD1  OR10P1  OR14I1  OR1F1  OR1L3  OR1N1  OR51B6  OR51I1  OR51Q1  OR52B6  OR6A2  OR7E12P  OR7E14P  OR8B8  ORAI1  ORC1  OSBPL10  OSTN  OVCH1  OXGR1  P2RX5  P2RX6  P2RX6P  P4HA3  PABPC1L2A  PACSIN1  SH3RF3  SHD  SHOX2  SIAH3  SIRT6  SIX4  SLC10A5  SLC12A1  SLC12A8  SLC14A2  SLC16A10  SLC16A12  SLC17A6  SLC17A7  SLC19A3  SLC1A6  SLC22A10  SLC22A13  SLC22A2  SLC22A24  SLC22A7  SLC22A9  SLC24A3  SLC24A4  SLC25A31  SLC26A3  SLC26A8  SLC27A2  SLC27A6  SLC29A2  SLC29A4  SLC30A2  SLC30A3  SLC32A1  SLC35F2  SLC35F4  SLC38A11  SLC38A8  SLC39A2  SLC39A3  SLC45A2  SLC46A2  SLC47A1  SLC4A3  THEM5  THEMIS  THPO  THSD7B  THY1  TIFA  TIGD2  TIGD4  TIMP4  TIPARP-AS1  TKTL1  TLE2  TLE6  TLL1  TMC3  TMC8  TMED11P  TMED6  TMEM121  TMEM130  TMEM132D  TMEM132E  TMEM143  TMEM150B  TMEM151B  TMEM163  TMEM173  TMEM191A  TMEM191B  TMEM196  TMEM215  TMEM221  TMEM233  TMEM244 | ASB9  ASGR1  ATF6B  ATG9B  ATP1A3  ATP1A4  ATP2A1  ATP2B2  ATP6V0CP3  ATP8B3  ATXN7L2  AURKC  AWAT1  B3GALT1  B9D1  BAAT  BCAS4  BCL2L15  BCRP3  BCYRN1  BDNF  BEST4  BFSP1  BFSP2  BHLHE22  BMP3  BMP4  BMP6  BOLL  BPI  BPIFB4  BRF2  BSPH1  BTBD11  BTNL9  BUB1  BUB1B  C10ORF82  C11ORF40  C11ORF91  C12ORF54  C12ORF71  DSCR4  DSP  DUSP14  DYDC2  ECE2  ECHDC3  ECM1  EDA2R  EDAR  EDN3  EEF1A2  EFCAB1  EFCAB10  EFNA3  EGF  EGFEM1P  EGFL6  EGOT  EGR4  EIF4E1B  ELANE  ELFN1  ELN  EME1  ENDOG  ENO1-AS1  ENPEP  EOMES  EPB41L4B  EPHA10  EPHA3  EPHB6  EPHX4  EPN3  EPX  ERAS  ERC2  EREG  ERP27  ESR1  ESYT3  EXD3  EXO1  F12  IFNA6  IFNW1  IGFBP3  IGFBP5  IGFL2  IGLON5  IGSF3  IL12RB2  IL13RA2  IL15RA  IL17B  IL18R1  IL23R  IL31RA  IL5RA  IL7  INPP5J  INSL5  INSM1  INSM2  IQCF3  IQGAP2  IQSEC3  IRF6  IRS4  ISYNA1  ITGBL1  JPH3  KAAG1  KANK4  KAZALD1  KBTBD13  KCNA1  KCNA3  KCNA4  KCNAB3  KCNB1  KCNB2  KCNG3  KCNH2  KCNH5  KCNH6  KCNH7  KCNIP2  MIR4265  MIR4273  MIR4274  MIR4278  MIR4279  MIR4282  MIR4288  MIR4295  MIR4301  MIR4302  MIR4306  MIR4311  MIR4312  MIR4316  MIR4318  MIR4423  MIR4439  MIR4446  MIR4450  MIR4462  MIR4472-2  MIR4476  MIR448  MIR4484  MIR4493  MIR4500  MIR4500HG  MIR4511  MIR4521  MIR4636  MIR4643  MIR4644  MIR4652  MIR4654  MIR4660  MIR4661  MIR4666A  MIR4676  MIR4677  MIR4681  MIR4684  MIR4703  MIR4710  MIR4716  PAK6  PALM3  PAM16  PANX2  PANX3  PARD6A  PATE1  PATE4  PAX7  PAX8  PCBP3  PCDH11Y  PCDH18  PCDH19  PCDHA1  PCDHA11  PCDHA12  PCDHA13  PCDHA2  PCDHA5  PCDHAC1  PCDHAC2  PCDHGA1  PCDHGC4  PCDHGC5  PCOLCE  PCOLCE2  PCSK1  PCSK2  PDC  PDE10A  PDE2A  PDE6A  PDGFD  PDLIM1  PDYN  PDZD7  PELI3  PEX26  PGAM2  PGAP3  PGLYRP1  PGPEP1L  PHLDA2  SLC5A4  SLC6A13  SLC6A17  SLC6A2  SLC6A20  SLC6A7  SLC7A3  SLC7A4  SLC7A9  SLC8A2  SLC8A3  SLC9A2  SLC9A3R2  SLCO1B3  SLCO2A1  SLCO4A1  SLIT2-IT1  SLIT3  SLITRK3  SLN  SMAGP  SMPX  SMTNL1  SMYD2  SNCB  SNED1  SNORA11  SNORA15  SNORA2B  SNORA35  SNORA36A  SNORA36C  SNORA52  SNORA53  SNORA5A  SNORA6  SNORA62  SNORA70D  SNORA70F  SNORA71D  SNORA79  SNORD111  SNORD113-1  SNORD113-2  TMEM44  TMEM52  TMEM61  TMEM63C  TMEM74B  TMIE  TMOD4  TMSB15A  TNFAIP8L1  TNFRSF19  TNFSF4  TNK1  TNN  TOMM34  TOP1MT  TOR2A  TP73  TPH1  TPH2  TPTE2P6  TPX2  TRAIP  TRH  TRHR  TRIB3  TRIM54  TRIM55  TRIM67  TRIM71  TRIP13  TRNP1  TRPC3  TRPC4  TRPC7 | C16ORF71  C16ORF86  C17ORF102  C17ORF107  C17ORF67  C17ORF98  C19ORF81  C1ORF194  C1ORF220  C1ORF53  C1ORF74  C1QL2  C1QTNF4  C1QTNF7  C1QTNF9B  C20ORF173  C22ORF24  C22ORF31  C2ORF40  C3ORF20  C4BPA  C4ORF36  C4ORF47  C6  C6ORF118  C6ORF222  C7ORF57  C7ORF66  C8ORF34  C9ORF131  CA11  CA4  CA7  CA9  CABP7  CACNA1C  CACNA1S  CACNG3  CALCR  CALR3  CALY  CAMK1G  F7  FABP4  FABP6  FAM110C  FAM117A  FAM129A  FAM153A  FAM173A  FAM185A  FAM189A1  FAM201A  FAM209A  FAM209B  FAM216B  FAM24B  FAM41C  FAM43A  FAM47A  FAM71C  FAM81B  FAM86HP  FAM89B  FAT2  FATE1  FBLIM1  FBLN2  FBLN7  FBN3  FBXL14  FBXL6  FBXO40  FBXO47  FBXW12  FBXW9  FCN3  FCRLB  FER1L6-AS1  FGD3  FGD5  FGF10  FGF13  FGFBP3  FHL2  FHL5  KCNIP4-IT1  KCNJ12  KCNJ6  KCNK3  KCNN1  KCNQ5  KCNS3  KCNT1  KCNV1  KCTD16  KDELC1  KDELR3  KHDC1L  KIAA0087  KIAA1210  KIAA1324  KIF26A  KIFC2  KISS1  KL  KLHDC8A  KLHL1  KLHL13  KLHL14  KLHL17  KLHL36  KLK10  KLK7  KLRB1  KLRG1  KMO  KNCN  KRT18  KRT18P55  KRT222  KRT31  KRT80  KRT81  KRT82  KRTAP5-9  L1TD1  LAMB3  LAMC2  LAMC3  MIR4718  MIR4728  MIR4729  MIR4730  MIR4731  MIR4732  MIR4744  MIR4748  MIR4752  MIR4753  MIR4754  MIR4760  MIR4774  MIR4777  MIR4786  MIR4793  MIR4802  MIR4803  MIR4804  MIR485  MIR491  MIR495  MIR496  MIR505  MIR510  MIR514A1  MIR520B  MIR521-1  MIR521-2  MIR522  MIR541  MIR545  MIR548A2  MIR548A3  MIR548AC  MIR548AD  MIR548AN  MIR548B  MIR548F3  MIR548F4  MIR548F5  MIR548H2  MIR548I4  MIR548Q  PHYHIP  PIF1  PIK3CD  PIN1P1  PIRT  PITPNM3  PKD2L1  PKDCC  PLA2G4E  PLAG1  PLCB4  PLCH1  PLCXD3  PLEKHG5  PLN  PLS1  PMCHL1  PMF1-BGLAP  PNLDC1  PNMA3  PNMA5  PNMT  PNPLA3  PODNL1  PODXL2  POLN  POSTN  POU1F1  POU5F1P4  PPARGC1B  PPM1E  PPP1R17  PPP1R1A  PPP3R2  PRB1  PRDM6  PRELID1  PRELID2  PRICKLE1  PRICKLE4  PRKAR1B  PRKG2  PRKY  PRMT8  SNORD113-5  SNORD113-6  SNORD113-7  SNORD113-9  SNORD114-1  SNORD114-10  SNORD114-13  SNORD114-14  SNORD114-15  SNORD114-16  SNORD114-17  SNORD114-18  SNORD114-2  SNORD114-20  SNORD114-21  SNORD114-22  SNORD114-23  SNORD114-24  SNORD114-25  SNORD114-26  SNORD114-27  SNORD114-28  SNORD114-29  SNORD114-3  SNORD114-30  SNORD114-31  SNORD114-4  SNORD114-5  SNORD114-7  SNORD114-9  SNORD115-1  SNORD115-10  SNORD115-16  SNORD115-22  SNORD115-24  SNORD115-25  SNORD115-31  SNORD115-32  SNORD115-35  SNORD115-40  SNORD115-45  SNORD115-47  SNORD115-48  SNORD115-7  TRPM5  TRPM8  TSGA13  TSHZ2  TSPAN2  TSSK3  TTC36  TTC9  TTTY19  TUBA8  TXK  TYRP1  UBE2S  UBE2T  UBE3D  UBQLNL  UBXN10  UCN  ULBP1  UNC13C  UNC5A  UPB1  UPK2  USP27X  UTS2  VASH2  VAX1  VENTXP7  VEPH1  VIPR2  VPS37D  VSIG2  VSTM2A  VTN | CAMK4  CAMKV  CARD10  CARD14  CARTPT  CAV1  CBLN1  CBLN2  CBLN4  CCBE1  CCDC110  CCDC120  CCDC141  CCDC154  CCDC157  CCDC158  CCDC60  CCDC62  CCDC81  CCDC89  CCKBR  CCL1  CCL13  CCL28  CCL7  CCNA1  CCNO  CD101  CD160  CD163L1  CD24  CD300LG  CD36  CD52  CD8A  CDA  CDCA4  CDCA5  CDH12  CDH3  CDH7  CDHR2  FIGLA  FLG  FLT3  FMO1  FMR1-AS1  FNDC5  FNDC8  FNDC9  FRAS1  FREM1  FRG2B  FRMPD4  FSCN3  FSD1  FTCD  FUCA1  FUT1  FUT2  FZR1  GABRA3  GABRA5  GABRA6  GABRD  GABRE  GABRG3  GABRR2  GAGE10  GAL3ST3  GALNT12  GALNT14  GALNT3  GALNT8  GAS2L2  GAS2L3  GBP1P1  GBP6  GCK  GCM1  GCNT1  GDA  GDAP1L1  GDF10  GDF5  GDPD3  LCE5A  LCN15  LDHC  LEF1  LEFTY1  LGALS8-AS1  LGI2  LHFPL1  LHX1  LHX6  LIN7A  LINC00184  LINC00189  LINC00290  LINC00308  LINC00313  LINC00314  LINC00327  LINC00421  LINC00426  LINC00460  LINC00469  LINC00477  LINC00488  LINC00525  LINC00535  LINC00538  LINC00575  LINGO1  LINGO2  LIPG  LIX1L  LMNB1  LMX1A  LNX1  LOXHD1  LPAR2  LRFN1  LRGUK  LRIT1  LRIT3  LRP5L  LRRC37A2  LRRC38  MIR548T  MIR548X  MIR561  MIR563  MIR564  MIR572  MIR580  MIR581  MIR583  MIR586  MIR590  MIR593  MIR595  MIR598  MIR599  MIR602  MIR603  MIR614  MIR626  MIR627  MIR628  MIR632  MIR647  MIR651  MIR654  MIR656  MIR659  MIR663B  MIR665  MIR668  MIR670  MIR7-1  MIR711  MIR7-3  MIR7-3HG  MIR758  MIR765  MIR770  MIR873  MIR889  MIR890  MIR921  MIR93  MIR940  PROK2  PROKR2  PROP1  PROS1  PROZ  PRPH  PRR16  PRR19  PRR4  PRRG2  PRSS12  PRSS16  PRSS23  PRSS3  PRSS38  PRSS50  PRSS55  PRSS8  PSKH2  PTCHD4  PTF1A  PTGER3  PTGES  PTGFR  PTHLH  PTP4A3  PTPN5  PTPRB  PTPRR  PTPRU  PVALB  PVR  PWRN1  PWRN2  PXDNL  PYDC1  QRFPR  QTRT1  RAB11FIP1  RAB15  RAB23  RAB26  RAB27B  RAB37  SNORD116-1  SNORD116-10  SNORD116-11  SNORD116-12  SNORD116-14  SNORD116-16  SNORD116-24  SNORD116-26  SNORD116-28  SNORD116-29  SNORD116-4  SNORD121B  SNORD127  SNORD18B  SNORD19B  SNORD32A  SNORD34  SNORD36A  SNORD36B  SNORD43  SNORD45C  SNORD49A  SNORD49B  SNORD56  SNORD59A  SNORD65  SNORD66  SNORD7  SNORD8  SNORD88A  SNORD9  SNORD90  SNORD92  SNORD94  SNTG1  SNX29P1  SOHLH1  SORCS1  SOX7  SPAG11A  SPAG4  SPATA18  SPATA4  SPDYE8P  VWC2  VWC2L  VWDE  WASH7P  WDR31  WDR64  WFDC13  WFDC3  WFDC9  WNT16  WNT3  WNT4  WNT5B  WSCD2  WWTR1  XCL1  YIPF2  ZAR1  ZBBX  ZBTB39  ZC3HAV1L  ZCCHC12  ZCCHC18  ZDHHC22  ZDHHC23  ZFAT-AS1  ZGLP1  ZMAT4  ZMAT5  ZMYND10  ZNF101  ZNF134  ZNF157  ZNF204P | CDK5R2  CDYL2  CEACAM19  CELA2A  CELF3  CENPA  CER1  CES5AP1  CHGA  CHODL-AS1  CHRDL2  CHRM1  CHRM2  CHRM3  CHRNA2  CHRNA3  CHRNB2  CHRNB3  CHST2  CHSY3  CKMT1B  CLDN16  CLEC2L  CLEC3B  CLEC4G  CLEC4GP1  CLGN  CLIC2  CLPS  CLSTN2  CMTM8  CNFN  CNGB1  CNIH2  CNTN5  COCH  COL19A1  COL22A1  COL25A1  COL4A1  COMTD1  CORO2A  GDPD5  GFER  GFRA2  GFRA3  GFRA4  GGNBP1  GGT1  GINS3  GIPC2  GJB7  GJD4  GLB1L2  GLB1L3  GLIS1  GLOD5  GLRA2  GLRA3  GLRA4  GLYATL2  GNAL  GNB3  GNRH2  GP6  GPC3  GPR12  GPR149  GPR26  GPR4  GPR45  GPR6  GPR61  GPR68  GPR84  GPR88  GPRIN1  GPX2  GRAMD1A  GRB14  GRB7  GREM2  GRHL1  GRHL2  GRIA1  GRIK1-AS1  LRRC55  LRRC61  LRRC72  LRRC73  LRRN4CL  LRRTM1  LRTM2  LSM11  LY6H  LY86-AS1  LYPD3  LYPD5  LYPD6  LYPD6B  MAB21L1  MACROD2-AS1  MADCAM1  MAEL  MAGEB3  MAGEC3  MAGEE2  MAGEL2  MANEAL  MANSC4  MAP1LC3C  MAPK13  MAST1  MC3R  MC4R  MCAT  MCF2L-AS1  MCHR1  MCHR2  MCM2  MCM3AP-AS1  MCOLN3  MCTP2  MDH1B  MED22  MEG8  MELK  MESP1  MET  MIR942  MKRN7P  MLLT10P1  MMP11  MMP12  MMP25  MMP3  MMP9  MMRN2  MPND  MPP4  MPP7  MPZ  MPZL2  MRAP2  MREG  MRM1  MSC  MSL3P1  MSS51  MT1DP  MTRNR2L3  MTRNR2L5  MTUS2  MYADML2  MYB  MYCNOS  MYH2  MYH7  MYL7  MYO1A  MYO1H  MYOG  N4BP3  NAGS  NAP1L6  NAT16  NAT8B  NBEAP1  NCR1  NDNF  NDST3  NDST4  NECAB2  RAB3B  RAB40A  RAB40AL  RAB41  RAD21-AS1  RAD21L1  RAD9B  RAMP2  RASD2  RASL11B  RASSF6  RBFOX1  RBM11  RBP5  RCAN3AS  RCN3  REP15  RERGL  RESP18  RFPL1  RFTN1  RFX8  RGL4  RGS7BP  RGS8  RHBDL1  RHO  RHPN1  RIBC2  RIMBP2  RIMS3  RIMS4  RIN1  RLN1  RMI2  RMST  RNF128  RNF150  RNF165  RNU6ATAC  ROPN1L  ROR2  RPGRIP1  RPH3A  SPEF1  SPHKAP  SPINK2  SPINK7  SPRN  SPRR2C  SPRR2F  SPRR2G  SRD5A1  SSTR1  ST6GALNAC2  ST8SIA2  ST8SIA3  STAR  STAT4  STBD1  STK32B  STOML3  STRA6  STX1A  STYK1  SULT1A2  SULT1C2P1  SULT4A1  SUMO1P1  SUN5  SV2B  SV2C  SVOP  SYCE1L  SYCE3  SYNDIG1L  SYNGR4  SYNPO  SYNPO2  SYNPR  SYT10  SYT12  SYT15  SYT16  SYT3  SYT4  SYT7  SYTL5  ZNF215  ZNF233  ZNF239  ZNF283  ZNF300  ZNF329  ZNF391  ZNF416  ZNF425  ZNF436  ZNF454  ZNF514  ZNF530  ZNF541  ZNF547  ZNF596  ZNF600  ZNF625  ZNF653  ZNF749  ZNF764  ZNF792  ZNF804A  ZNF804B  ZNF833P  ZNF876P  ZNF890P  ZP1  ZPLD1  ZSCAN1  ZYG11A |

| **Oligodendrocytes** | | | | | |  |
| --- | --- | --- | --- | --- | --- | --- |
| 0-Jan  8-Mar  4-Sep  12-Sep  ABCA2  ABCG1  ABHD11-AS1  ABLIM2  ACSM1  ACVR1  ADAM8  ADAMTS1  ADAMTS18  ADAMTS5  ADAMTS8  ADCY5  AGMO  AGPAT1  AKNAD1  AMBRA1  ANGPTL2  ANGPTL5  ANKRA2  ANKRD18A  ANKRD37  ANKRD62P1-PARP4P3  ANKRD65  ANP32C  ANXA9  APBA3  APOA1  AQP6  AQP8  ARC  ARHGAP19  ARHGEF19  ARHGEF37  ARL10  ARMC4  ARMC7  ARRDC2  ASB13  IKBKG  INSL3  IP6K3  IPO13  IQCC  IRF2BP1  IRX2  ITGA4  ITGB1BP2  ITM2A  JAM3  KBTBD7  KCNG1  KCNK10  KCNV2  KDR  KEL  KIAA1324L  KIF13B  KIF25  KIF6  KIFC3  KLF17  KLHL31  KLHL32  KLHL4  KLK6  KLRC2  KLRC3  KLRC4  KRTAP5-8  KSR1  LACC1  LARP6  LAYN  LCORL  LCTL  LDB3  LDLRAD3  LDLRAP1  LGI3  LGR5  LHFPL3  LIAS  SAMD3  SAP25  SAPCD2  SARM1  SAYSD1  SCARNA2  SCARNA23  SCGB1B2P  SELL  SEMA3B  SEMA3C  SERPINA3  SERPINE3  SERTAD4  SFRP1  SFRP4  SGK2  SGK3  SH3TC2  SHB  SHC4  SHISA4  SHROOM4  SLC24A2  SLC25A13  SLC35A5  SLC3A1  SLC43A3  SLC45A3 | ASPHD1  ATOH7  ATP10B  AZGP1  B3GNT9  BACE2  BAMBI  BCHE  BCL2L14  BIRC5  BLID  BMP1  BMP8B  BOK  BRD1  BRSK1  BST1  BTBD16  BTN2A2  BVES  C10ORF90  C11ORF42  C11ORF71  C12ORF66  C19ORF24  C19ORF25  C1GALT1C1  C1ORF226  C21ORF91-OT1  C2ORF27A  C7ORF25  C7ORF31  C7ORF61  C9ORF135  C9ORF3  C9ORF43  CA14  CACNA2D4  CACNG1  CAGE1  CALD1  CAMKMT  LINC00113  LINC00161  LINC00323  LINC00486  LMCD1  LMF1  LMOD2  LMOD3  LOX  LOXL2  LPAL2  LPAR1  LRRC4B  LRRC66  LRRD1  LRWD1  MAD1L1  MAG  MAL  MAP3K10  MAP6D1  MATN1  MATN2  MBP  MCM8  MDFI  MDS2  MED18  MED24  METRN  MICALL1  MID1IP1  MIF4GD  MIPEP  MIR1200  MIR122  MIR1243  MIR1248  MIR1250  MIR1262  MIR1278  MIR1292  MIR1304  MIR148B  SLC4A9  SLC5A11  SLC5A5  SLC6A12  SLCO1A2  SLCO1B1  SNORA11C  SNORA13  SNORA16A  SNORA18  SNORA20  SNORA33  SNORA38B  SNORA40  SNORA51  SNORA54  SNORA70C  SNORA71B  SNORD110  SNORD114-12  SNORD115-39  SNORD5  SNORD51  SNORD61  SOSTDC1  SOX10  SPAG6 | CAPN3  CARNS1  CATSPERD  CAV2  CBLC  CCDC122  CCDC150  CCDC160  CCDC17  CCDC61  CCNE2  CD22  CDC14C  CDC20  CDC25C  CDC42EP1  CDC42EP2  CDC6  CDCA7L  CDH1  CDH19  CDK18  CDKN1C  CDKN2A  CEACAM1  CENPI  CENPN  CEP128  CERCAM  CES5A  CHADL  CHCHD4  CHEK1  CHI3L2  CHODL  CHPF  CHRNA10  CHST6  CIDEA  CILP  CKAP2L  CLASRP  CLCA4  MIR16-2  MIR181A1  MIR181B1  MIR25  MIR302A  MIR302B  MIR302C  MIR302D  MIR3074  MIR3121  MIR3157  MIR3173  MIR320E  MIR3605  MIR3661  MIR367  MIR3973  MIR4271  MIR4276  MIR4305  MIR4326  MIR4434  MIR4436A  MIR4712  MIR4714  MIR4742  MIR4783  MIR4790  MIR517A  MIR520G  MIR548W  MIR558  MIR573  MIR604  MIR624  MIR629  MIR635  MIR639  MIR761  MIR933  MIRLET7A1  MIRLET7F1  MMAA  MMACHC  SPARC  SPIRE2  SPTLC1  SRD5A3  ST18  STEAP1B  STK31  STK39  STRN  STX10  SUGT1P3  TAF5L  TAS2R31  TAS2R39  TAX1BP3  TBRG4  TCEANC  TCP11L2  TEKT1  TEKT3  TEKT4P2  TEX14  TF  TGFA  THBS2  THOC6  TMC6  TMCC2  TMCO2 | CLDN11  CLEC12B  CLEC14A  CLIP2  CLMN  CLSPN  CMTM5  CNBD1  CNKSR3  CNN2  CNP  CNTF  CNTN2  COL18A1  COL4A5  COL9A3  COMMD3-BMI1  COX11  CPXM2  CRB2  CTDSP1  CTHRC1  CTNNA3  CUTC  CXADRP2  CYB5R2  CYP2C18  DBNDD2  DDX4  DDX49  DENND2D  DEPDC1B  DGKH  DISC1  DLEU1  DLL1  DMRT2  DMRTA2  DNAH11  DNAJC22  DOCK5  DOK1  MMP21  MMRN1  MND1  MNS1  MOB3B  MOBP  MOG  MON1A  MOV10L1  MPST  MS4A2  MSH4  MTCP1  MTFR1  MYBL1  MYO1D  MYOT  NACAD  NAGPA  NAT1  NCAPD2  NCAPH  NEK3  NFE2  NGFR  NHLH2  NID2  NINJ2  NKAIN1  NKAIN2  NKX6-2  NMI  NMUR2  NOP14-AS1  NPAS1  NUDT8  NUF2  ONECUT2  OR2L13  OR2L1P  OR5AP2  OR6W1P  OR7A5  ORM2  TMEM125  TMEM139  TMEM140  TMEM171  TMEM182  TMEM198  TMEM235  TMEM241  TMEM26  TMEM31  TMEM63A  TMEM64  TMEM79  TMEM86B  TMEM95  TMEM98  TNFSF10  TNFSF9  TNIP2  TNNC1  TNNI3  TNS1  TPPP  TPRN  TPST1  TRAPPC9  TRIM62  TRPC5  TRPC6 | DPH3P1  DPY19L2P3  DPYD  DPYSL5  DUSP18  DYNLL2  E2F5  EDEM2  EFNB1  EML1  ENPP2  ENPP6  EPCAM  EPHB2  ERMN  ESRP1  ETFB  EXTL1  FA2H  FAM122B  FAM177B  FAM20A  FAM83D  FANCB  FGF7  FLNC  FLVCR2  FMO3  FMO4  FOLH1B  FOXO4  FRMD4B  FRMD5  FUT8  FXYD4  FZD5  GALNT6  GAS1  GATA2  GCA  GCC1  GDF9  GHSR  OSGIN1  P2RX5-TAX1BP3  PABPC5  PACSIN3  PAIP2B  PALB2  PAQR4  PARP15  PARP16  PCA3  PCDHGB2  PCGF6  PCNA  PCSK6  PCYT2  PDIK1L  PEX16  PEX5L  PHLDA3  PIEZO2  PIGN  PIP  PIP4K2C  PLA2G4B  PLCL1  PLD1  PLEKHG3  PLEKHH1  PLLP  PLP1  POGK  POLA2  POLR1B  POLR1E  POLR2C  PON1  POU2F3  PP2D1  PPFIBP2  PPM1N  PPP1R14A  PPP1R16B  PPP1R36  TRPM6  TSIX  TSPAN1  TSPAN18  TSPAN8  TSSK4  TTC23  TTC30B  TUBB4A  UBE2C  UBE2MP1  UGT8  VSX1  VWA1  WDR18  WDR66  ZBTB2  ZBTB6  ZDHHC24  ZFP1  ZFP3  ZNF137P  ZNF155  ZNF184  ZNF205  ZNF213  ZNF227  ZNF319  ZNF354A | GINS4  GJB1  GJC2  GJC3  GLT8D2  GLTPD2  GNA11  GNB1L  GNG8  GNRHR2  GOLGA6C  GOLT1A  GPD1  GPIHBP1  GPR62  GPSM2  GRB10  GREM1  GRWD1  GTF2E1  GUSBP3  GZF1  HAPLN2  HAUS2  HCG4B  HERC5  HFE  HHIP  HHLA3  HIST1H1T  HIST3H2BB  HMG20B  HMGA1P7  HOXD1  HPN  HR  HRASLS2  HS6ST2  HSD11B1  HSD17B3  ID1  ID3  IGSF22  PPP1R37  PPP1R8  PPP2R3A  PRDM8  PRIMA1  PROM1  PRR11  PRR5L  PRRG1  PRUNE2  PSG10P  PSG5  PTK7  PTPDC1  PTPRK  PWWP2A  PYY2  QSOX2  RAB28  RAB33A  RAB3IP  RAD51AP1  RASAL1  RBM24  RBM42  RBP7  REPS2  RGS3  RHBDL2  RHOBTB1  RHOV  RNASE1  RNASE4  RNASEH2A  RNF125  RNF144A  RNF220  RNU11  ROBO4  ROM1  RPL39L  RTKN  RWDD2B  S1PR5  ZNF354C  ZNF358  ZNF367  ZNF473  ZNF486  ZNF488  ZNF491  ZNF510  ZNF536  ZNF563  ZNF576  ZNF582  ZNF585B  ZNF607  ZNF676  ZNF688  ZNF717  ZNF727  ZNF778  ZSCAN12P1  ZSCAN16  ZSCAN29  ZWINT | |

| **Microglia** | | | | | |  |
| --- | --- | --- | --- | --- | --- | --- |
| A2M  ABCB4  ABCC4  ABCG2  ABHD8  ABI3  ABL2  ACPP  ACY3  ADAM28  ADAMDEC1  ADAMTSL4  ADAP2  ADORA3  ADPRH  ADRA2B  AGMAT  AIF1  AKNA  ALOX5  ALOX5AP  ALPK2  ALPK3  AMELX  ANKRD13D  ANKRD22  AOAH  APBB1IP  APLF  APOBEC3C  APOC1  APOC1P1  APOC4-APOC2  APOL1  ARGFXP2  ARHGAP15  ARHGAP25  ARHGAP6  ARHGDIB  ARID3B  ARID5A  ARL11  GIMAP4  GIMAP7  GIMAP8  GIPR  GLRX  GM2A  GMFG  GMPR  GNA15  GNLY  GOLPH3L  GPR160  GPR183  GPR34  GPR65  GPRIN3  GPX1  GRASP  GSTM1  GYPC  HAL  HAMP  HAVCR1  HAVCR2  HCCS  HCK  HCLS1  HCST  HENMT1  HEXB  HHEX  HIF1A  HIST1H4E  HIST2H2AB  HLA-B  HLA-DMA  HLA-DPB1  HLA-DQA2  HLA-DQB2  HLA-DRA  HLA-DRB1  HLA-DRB5  HPGDS  HS3ST1  PPP4C  PRDM1  PRKCH  PRRG4  PSG11  PSG6  PSMB8  PSTPIP2  PTAFR  PTGER4  PTGS1  PTPN6  PTPRC  PXDN  PYCARD  RAB20  RAB32  RAB39A  RASAL3  RASSF3  RBBP8  RBM47  RBP3  RCAN3  RCC2  RCVRN  REL  RELB  RELL2  RFX1  RGS1  RGS10  RGS13  RGS18  RGS19  RHBDF2  RHOG  RHOH | ARRDC5  ARSA  ATP6V0A4  ATP8B4  ATXN1  B3GNT5  B3GNTL1  B4GALT1  BACH1  BATF3  BCL2A1  BIN2  BIN3  BLNK  BNC2  BNIP2  BRAP  BST2  C14ORF178  C15ORF39  C16ORF54  C16ORF89  C18ORF21  C19ORF38  C1ORF162  C1QA  C1QB  C1QC  C22ORF46  C3  C3AR1  C4ORF19  C5AR1  C5ORF58  C9ORF66  CAHM  CAPG  CAPNS2  CARD16  CASP1  CASP10  CASP4  CASP6  HSD3B7  HSPB7  ICAM2  ICOS  IER3  IFI16  IFI30  IFI44L  IFNGR2  IGFLR1  IGLL3P  IGSF10  IGSF6  IKBIP  IKZF1  IL10RA  IL13RA1  IL16  IL18  IL1A  IL1B  IL1RAP  IL1RL2  IL1RN  IL20RA  IL27  IL4I1  IL4R  IL6R  INHBA  INPP5D  IPCEF1  IRAK2  IRAK3  IRF5  IRF8  ISG20  ITGAD  ITGAM  ITGAX  ITGB2  ITGB7  ITIH2  ITPRIP  RNASE2  RNASE3  RNASE6  RNASET2  RNF103  RNF122  RNF135  RNF144B  RNF20  RNF222  RNLS  RNPEP  RNU12  RPIA  RPS6KA1  RTP4  RUNX1  RUNX2  RXFP2  S100A11  S100A8  SAA2  SAMD9L  SAMSN1  SASH3  SCARNA17  SCIMP  SCIN  SELPLG  SERPINA1  SERPINB9  SERPINE1  SERPINF1  SFT2D3  SGK1  SGPP1  SGTA  SH2B3 | CASP7  CASS4  CBFB  CCDC69  CCL2  CCL20  CCL22  CCL3  CCL3L1  CCL4  CCR1  CCR5  CCRL2  CD14  CD276  CD300A  CD300C  CD300E  CD300LB  CD302  CD33  CD37  CD4  CD44  CD53  CD68  CD69  CD74  CD83  CD84  CD86  CDCP1  CDK2  CDKL4  CDKN1A  CDRT4  CEBPG  CENPBD1  CEP55  CH25H  CHCHD7  CHST14  CHST3  IZUMO1  JDP2  KBTBD8  KCNE3  KCNK2  KCNMB1  KCNN4  KCTD12  KDM6B  KIAA0040  KIF17  KLF2  KLF6  KLHL6  KRT15  KYNU  LAIR1  LAPTM5  LAT2  LCP1  LCP2  LENG1  LHFPL2  LILRA4  LILRB1  LILRB3  LILRB4  LIMD2  LINC00309  LINC00346  LIPC  LNP1  LPAR5  LPAR6  LPCAT2  LPL  LRMP  LRRC29  LRRC70  LTC4S  LY86  LY96  LYN  LYVE1  SH3BP1  SH3TC1  SHISA2  SIGLEC10  SIGLEC14  SIGLEC5  SIGLEC8  SIKE1  SIPA1  SKAP2  SLA  SLC11A1  SLC15A3  SLC1A5  SLC25A25  SLC2A14  SLC2A5  SLC2A9  SLC35A3  SLC39A8  SLC7A7  SLCO2B1  SMAD3  SNORA72  SNORA84  SNORA9  SNORD45A  SNORD45B  SNORD4B  SNORD54  SNORD56B  SNORD82  SNORD89  SOAT1  SOCS6  SP100  SP140L  SPHK1 | CHST9  CHSY1  CITED2  CLEC17A  CLEC2B  CLEC7A  CLEC9A  CLECL1  CLPB  CMTM3  CMTM6  CMTM7  CNGA1  COL8A2  CORO1A  CPVL  CREB3  CRISPLD2  CRYBB1  CSF1R  CSF3R  CTLA4  CTSC  CTSS  CTSW  CTTNBP2NL  CX3CR1  CXCL12  CXCL16  CXCR3  CXORF21  CYBA  CYBB  CYP19A1  CYSLTR1  CYTIP  CYTL1  DAB2  DAGLB  DAPP1  DCTD  DDR1  DDX39A  LYZ  LZTS1  MAD2L1  MAFB  MAML3  MAN1A1  MAP2K3  MAP3K8  MAP4K2  MAPK12  MDK  MEST  METTL24  MFSD2A  MICAL1  MIER2  MILR1  MIR1296  MIR1343  MIR155HG  MIR190A  MIR197  MIR221  MIR2355  MIR2467  MIR26B  MIR29B2  MIR3128  MIR3651  MIR3660  MIR4257  MIR4637  MIR4723  MIR4727  MIR548G  MIR548I2  MIR578  MIR623  MIR645  MIRLET7D  MIRLET7G  MIS18BP1  MLXIPL  MMP19  SPN  SPNS3  SPP1  SRGN  ST6GAL1  ST8SIA4  STAT6  STC1  STK10  STX11  STX19  STXBP2  SUMO4  SUSD1  SUSD3  SYK  SYNDIG1  TAGAP  TAL1  TAS2R19  TBC1D14  TBXAS1  TCIRG1  TCOF1  TFCP2L1  TFEC  TGFBR2  THBD  TIGIT  TINAG  TLCD2  TLR1  TLR10  TLR2  TLR6  TLR7  TM4SF20  TM6SF1 | DEF6  DENND1C  DENND3  DGKA  DHRS9  DKK1  DLEU7  DLGAP3  DNAH1  DNAJB7  DNMBP  DOCK2  DOCK8  DOK2  DOK3  DR1  DUSP5  DUSP6  E2F3  EBI3  EDEM1  EDN1  EGFL7  EGR2  EGR3  EHD4  EIF4EBP1  ELF1  EMB  EMP1  ENG  ENPP1  ENTPD4  EPHA1  EPSTI1  ESR2  ETF1  ETS2  EVI2B  F11R  F13A1  FAM102B  FAM135A  MNDA  MOB1A  MPEG1  MRC1  MS4A14  MS4A4A  MS4A6A  MS4A7  MSR1  MSX1  MTHFD2  MTHFS  MX2  MYBPC3  MYC  MYD88  MYL5  MYO1C  MYO1F  MYO7A  MYOF  NAPSB  NCF2  NCF4  NCKAP1L  NEDD9  NET1  NFKBIZ  NHLRC1  NIPAL4  NKX3-1  NLRP3  NOD1  NODAL  NR1H2  NUAK2  OAS1  OASL  OGFRL1  OLFML3  OLR1  OR56B1  OSCAR  OSM  TMEM104  TMEM106A  TMEM119  TMEM156  TMEM71  TMEM88  TMPRSS12  TNFAIP8  TNFRSF10A  TNFRSF10B  TNFRSF10D  TNFRSF12A  TNFRSF1B  TNFRSF21  TNFSF13B  TNFSF14  TNFSF15  TNFSF18  TNFSF8  TOP3B  TP53RK  TP53TG5  TRAF3IP3  TRAPPC6A  TREM2  TREML1  TRIB1  TRIM14  TRIM22  TRIM32  TRIM38  TTLL2  TTLL3  TUBB6  TXLNB  TYROBP  UBA7  UBXN11 | FAM186B  FAM72A  FBP1  FBXL18  FBXO30  FCAR  FCER1G  FCGBP  FCGR1A  FCGR1B  FCGR2A  FCGR3A  FCGRT  FERMT3  FFAR2  FGD2  FGL2  FGR  FHL3  FHOD1  FLI1  FLT1  FMN1  FOLR2  FOSL1  FOSL2  FOXD4L1  FPGS  FPR1  FPR3  FRRS1  FUT4  G0S2  GAB3  GABPB1  GAL3ST4  GBP1  GBP2  GBP4  GCH1  GCNT7  GGTA1P  GIMAP2  OXER1  P2RX4  P2RY12  P2RY13  P2RY6  P4HA1  PAN3-AS1  PANX1  PARP12  PARVG  PAX5  PDE3B  PDGFB  PDPN  PDZK1  PECAM1  PGM5  PIGM  PIGR  PIGW  PIK3AP1  PIK3CG  PIK3R5  PILRA  PKD2L2  PKDREJ  PLA1A  PLA2G4A  PLA2G7  PLAU  PLAUR  PLCB2  PLD4  PLEK  PLEKHG2  PLIN2  PLK3  PLK4  PLP2  PLVAP  PMAIP1  PMEL  POTEE  PPARG  UCP2  USP53  UST  VAMP8  VCAN  VENTX  VMO1  VSIG4  VTRNA1-1  VTRNA1-2  WAS  WDFY4  WNT8B  XBP1  XIRP1  YDJC  ZBTB1  ZC3H12A  ZDHHC3  ZEB2-AS1  ZFP36L2  ZFP91-CNTF  ZMYND15  ZNF160  ZNF23  ZNF3  ZNF321P  ZNF468  ZNF474  ZNF550  ZNF584  ZNF678  ZNF85  ZNF865  ZNRF2 | |
