# Supplementary material for: Bridging the Gap Between Morphometric Similarity Mapping and Gene Transcription in Alzheimer’s Disease
Source: Front Neurosci. 2021 Sep 29;15:731292. doi: 10.3389/fnins.2021.731292 (PMC8522649; doi:10.3389/fnins.2021.731292)
Supplement: Supplementary file 7 [file Table_5.DOCX]

**Table S5** GO enrichment results for PLS1 gene sets

| **ID** | **Description** | **Gene Ratio** | **Bg Ratio** | **pvalue** | **p.adjust** | **qvalue** | **Count** |
| --- | --- | --- | --- | --- | --- | --- | --- |
| PLS1 negative genes（[bonferroni](https://baike.so.com/doc/6838484-7055747.html) corrected） | | | | | | | |
| GO:0050804 | modulation of chemical synaptic transmission | 70/1418 | 436/18670 | 0.000 | 0.000 | 0.000 | 70 |
| GO:0099177 | regulation of trans-synaptic signaling | 70/1418 | 437/18670 | 0.000 | 0.000 | 0.000 | 70 |
| GO:0060078 | regulation of postsynaptic membrane potential | 32/1418 | 140/18670 | 0.000 | 0.000 | 0.000 | 32 |
| GO:0042391 | regulation of membrane potential | 65/1418 | 434/18670 | 0.000 | 0.001 | 0.000 | 65 |
| GO:0099003 | vesicle-mediated transport in synapse | 39/1418 | 207/18670 | 0.000 | 0.001 | 0.000 | 39 |
| GO:0050808 | synapse organization | 60/1418 | 408/18670 | 0.000 | 0.005 | 0.001 | 60 |
| GO:0007015 | actin filament organization | 59/1418 | 400/18670 | 0.000 | 0.005 | 0.001 | 59 |
| GO:0099504 | synaptic vesicle cycle | 35/1418 | 194/18670 | 0.000 | 0.011 | 0.001 | 35 |
| GO:0071248 | cellular response to metal ion | 34/1418 | 190/18670 | 0.000 | 0.018 | 0.002 | 34 |
| GO:0006836 | neurotransmitter transport | 43/1418 | 269/18670 | 0.000 | 0.021 | 0.002 | 43 |
| GO:0007409 | axonogenesis | 64/1418 | 468/18670 | 0.000 | 0.025 | 0.002 | 64 |
| GO:0050806 | positive regulation of synaptic transmission | 31/1418 | 168/18670 | 0.000 | 0.026 | 0.002 | 31 |
| GO:0050890 | cognition | 45/1418 | 296/18670 | 0.000 | 0.048 | 0.003 | 45 |
| PLS1 positive genes（[bonferroni](https://baike.so.com/doc/6838484-7055747.html) corrected） | | | | | | | |
| GO:0071804 | cellular potassium ion transport | 45/1642 | 217/18670 | 0.000 | 0.000 | 0.000 | 45 |
| GO:0071805 | potassium ion transmembrane transport | 45/1642 | 217/18670 | 0.000 | 0.000 | 0.000 | 45 |
| GO:0006813 | potassium ion transport | 47/1642 | 240/18670 | 0.000 | 0.001 | 0.000 | 47 |
| GO:1990778 | protein localization to cell periphery | 52/1642 | 311/18670 | 0.000 | 0.039 | 0.009 | 52 |

| **ID** | **geneID** |
| --- | --- |
| **PLS1 negative genes（**[**bonferroni**](https://baike.so.com/doc/6838484-7055747.html) **corrected）** | |
| GO:0050804 | TAC1/SYNPO/DGKB/RIN1/DLG4/GIT1/NEUROD2/NPTX2/SNAP47/NPY5R/OPHN1/FAM107A/SLC7A11/CALB1/NR2E1/ADRB2/STX1A/MCTP1/VAMP2/IL1B/GRID2/SNCG/MPP2/BDNF/HRAS/TUBB2B/OXTR/NTNG2/JPH4/APOE/DRD5/BAIAP2/CALB2/SLC8A2/GRIK3/RAB3GAP1/GRIK5/GRIN2B/NISCH/APBA1/GRIK2/KCNMB4/NPTXR/YWHAH/RGS14/MAPK1/SHANK1/CPLX3/CASK/NLGN3/BCR/CALM3/CACNG3/CNR1/CYP46A1/SCGN/HRH1/PTPRA/TMEM108/NTSR1/EFNB3/ARRB2/GRM1/CNIH2/DTNBP1/BAIAP3/GRIA1/SNCA/GRIN3A/PRKCG |
| GO:0099177 | TAC1/SYNPO/DGKB/RIN1/DLG4/GIT1/NEUROD2/NPTX2/SNAP47/NPY5R/OPHN1/FAM107A/SLC7A11/CALB1/NR2E1/ADRB2/STX1A/MCTP1/VAMP2/IL1B/GRID2/SNCG/MPP2/BDNF/HRAS/TUBB2B/OXTR/NTNG2/JPH4/APOE/DRD5/BAIAP2/CALB2/SLC8A2/GRIK3/RAB3GAP1/GRIK5/GRIN2B/NISCH/APBA1/GRIK2/KCNMB4/NPTXR/YWHAH/RGS14/MAPK1/SHANK1/CPLX3/CASK/NLGN3/BCR/CALM3/CACNG3/CNR1/CYP46A1/SCGN/HRH1/PTPRA/TMEM108/NTSR1/EFNB3/ARRB2/GRM1/CNIH2/DTNBP1/BAIAP3/GRIA1/SNCA/GRIN3A/PRKCG |
| GO:0060078 | GABRE/DLG4/ADRB2/STX1A/GRID2/GABRG1/MPP2/BAIAP2/GABBR1/GRIK3/RAB3GAP1/GABRA2/GRIK5/GRIN2B/SLC17A7/GRIK2/GLRA3/SHANK1/NLGN3/P2RX5/GLRA2/GABRB3/TMEM108/NTSR1/ARRB2/GRM1/GRIA1/SNCA/GABRA3/GRIN3A/GABRA5/GABRB1 |
| GO:0042391 | TAC1/GABRE/STOX1/KCNE2/SCN7A/GBA/DLG4/TRDN/CACNA1H/FKBP1B/SLC1A6/SNTA1/ADRB2/STX1A/GRID2/DMD/GABRG1/MPP2/SCN3A/WDR1/NPPA/BAIAP2/NTSR2/GJA1/GABBR1/PYCR1/JUN/GRIK3/RAB3GAP1/GABRA2/CD36/GRIK5/NEDD4L/GRIN2B/SLC17A7/CXADR/GRIK2/HTR3A/KCNMB4/YWHAH/GLRA3/SHANK1/NLGN3/GPR88/KCNK13/CAMK2D/SCN9A/KCNK2/P2RX5/CNR1/GLRA2/GABRB3/TMEM108/NTSR1/ARRB2/GRM1/CNIH2/GRIA1/SNCA/GABRA3/GRIN3A/SCN3B/GABRA5/PID1/GABRB1 |
| GO:0099003 | PACSIN1/GIT1/PCLO/SNAP47/ERC2/OPHN1/GSG1L/HPCA/STX1A/VAMP2/DOC2A/BTBD9/SLC2A4/STON2/RAB3GAP1/GRIK5/APBA1/SLC17A7/SNAP29/EFNB2/POTEKP/AP2B1/CPLX3/CASK/NLGN3/AP3B2/CALM3/AP3M2/CANX/CNR1/ACTG1/CNIH2/DTNBP1/SNCA/AP3S1/GRIN3A/PRKCG/SYT17/RAB27B |
| GO:0050808 | PCDHGC3/UBE3A/L1CAM/SYNPO/DGKB/DBNL/DLG4/C1QA/PCLO/NEUROD2/PALM/ERC2/SLITRK2/LRRC24/ABI2/SDK2/OPHN1/SLC7A11/FLRT1/SLC9A6/GRID2/SNCG/BDNF/CDH8/WNT7B/PTPRO/OXTR/NTNG2/GPM6A/APOE/CTNND2/BAIAP2/PDLIM5/CDC42/GABRA2/AMIGO2/GRIN2B/ERBB2/CAMKV/EFNB2/YWHAZ/POTEKP/MDGA1/SHANK1/SEMA4A/NLGN3/FARP1/FZD1/DLG2/ROBO2/GPC4/CDH9/GABRB3/ACTG1/TMEM108/SNCA/PTPRF/TUBB/SLIT1/C1QL1 |
| GO:0007015 | PACSIN1/PDLIM3/LCP1/TCAP/TAC1/SYNPO/BRK1/PFN3/DBNL/MYOM1/PYCARD/ALDOA/ABI2/SCIN/FAM107A/TMSB4X/KPTN/ARPC2/CDC42EP4/ACTR3B/ARRB1/S100A10/ARPC5/SAMD14/ARHGAP6/WDR1/CAPZB/PPM1F/SORBS2/BAIAP2/DIAPH2/IGSF22/ARHGAP18/MAD2L2/ARPC4/HSP90B1/CDC42/IQGAP2/RHOC/CAPG/MARCKS/WNT4/ABI1/SHANK1/SMAD3/RAC2/MTPN/CAP1/ARHGAP28/PTGER4/TMEFF2/GDPD2/TPM3/GMFB/TRIM27/TMSB10/CORO1A/DPYSL3/PRKCD |
| GO:0099504 | PACSIN1/GIT1/PCLO/SNAP47/ERC2/OPHN1/SLC17A8/STX1A/VAMP2/DOC2A/BTBD9/SLC2A4/STON2/TH/RAB3GAP1/GRIK5/APBA1/SLC17A7/SNAP29/RAPGEF4/POTEKP/CPLX3/CASK/NLGN3/CALM3/CANX/SYN2/CNR1/ACTG1/DTNBP1/SNCA/GRIN3A/PRKCG/SYT17/RAB27B |
| GO:0071248 | CRHBP/CYBB/TPH2/DLG4/NEUROD2/MT1G/RYR3/MT1A/CACNA1H/SYT10/ADCY7/MT1B/GUCA1A/HPCA/MAP1LC3A/OGG1/CPNE5/MT2A/JUN/TH/PRKAA2/SLC25A23/MAPK1/DLG2/CAMK2D/GLRA2/CEBPA/SLC30A10/CPNE6/SNCA/RASAL1/MAPK3/SYT17/CPNE7 |
| GO:0006836 | SYNGR3/GIT1/PCLO/SNAP47/ERC2/SLC44A2/LILRB1/SLC7A11/SLC1A6/SLC17A8/STX1A/MCTP1/LIN7B/VAMP2/DOC2A/SNCG/NPPA/TRPC4/MAOB/HTR2C/TH/RAB3GAP1/GABRA2/SLC25A22/GRIK5/APBA1/SLC17A7/SNAP29/KCNMB4/PRAF2/CPLX3/CASK/CALM3/SYN2/CNR1/NTSR1/DTNBP1/BAIAP3/SNCA/HTR1A/GRIN3A/PRKCG/SYT17 |
| GO:0007409 | DAB1/SEMA6B/VANGL2/LHX2/L1CAM/NDN/VLDLR/ALCAM/DBNL/ATP8A2/PALLD/B4GALT5/TRPV2/PARD3/SLITRK2/GLI2/OPHN1/LINGO1/CSF1R/SLC9A6/NR2E1/TCTN1/PLXNA1/MAP2K1/BDNF/PTCH1/TUBB2B/PTPRO/NTNG2/APOE/BAIAP2/ISLR2/DOCK7/NUMBL/SEMA3D/LAMA1/EPHA8/ERBB2/TNN/HSP90AB1/RAP1GAP/DOK1/EFNB2/CRMP1/PLXNC1/MAPK1/SEMA4A/NLGN3/USP9X/RAC2/ROBO2/SLIT3/UCHL1/DOK4/ROBO1/PTPRA/SEMA4F/EFNB3/STK25/CDH4/MAPK3/DOK6/ARHGDIA/SLIT1 |
| GO:0050806 | TAC1/DLG4/SNAP47/FAM107A/CALB1/NR2E1/ADRB2/STX1A/VAMP2/MPP2/OXTR/APOE/BAIAP2/CALB2/SLC8A2/RAB3GAP1/GRIN2B/GRIK2/RGS14/MAPK1/SHANK1/NLGN3/CACNG3/CNR1/CYP46A1/SCGN/NTSR1/ARRB2/DTNBP1/BAIAP3/SNCA |
| GO:0050890 | CRHBP/TAC1/SYNPO/RIN1/VLDLR/DLG4/NEUROD2/NPTX2/FAM107A/SLC7A11/CALB1/OPRK1/JAKMIP1/BTBD9/BDNF/OXTR/JPH4/APOE/DRD5/SLC8A2/JUN/TH/HMGCR/GRIN2B/SLC17A7/RGS14/MAPK1/SHANK1/NLGN3/GPR88/RIC8A/KCNK2/B4GALT2/TUSC3/THRA/CNR1/HRH1/NTSR1/PTCHD1/GRIA1/SLC1A4/GMFB/PRKCG/GABRA5/C1QL1 |
| **PLS1 positive genes（**[**bonferroni**](https://baike.so.com/doc/6838484-7055747.html) **corrected）** | |
| GO:0071804 | KCNAB3/KCNT1/ANK3/KCNJ3/ATP4A/SLC24A2/ABCC8/KCNC1/SLC9A1/HCN1/SNAP25/SLC12A5/KCNA2/KCNS1/KCNH6/KCNA1/KCNB1/KCNC3/KLHL24/CAB39/KCNMB3/OXSR1/KCNK12/KCNQ5/HCN2/ATP1B3/KCNIP3/LRRC38/ATP1B1/KCNJ14/ATP1A3/ATP1A4/KCNC4/SLC9A5/KCNA6/MIR133A1/KCNJ11/EDN3/KCNS3/ATP1A1/SLC12A8/KCNRG/KCNS2/PKD2/SLC12A2 |
| GO:0071805 | KCNAB3/KCNT1/ANK3/KCNJ3/ATP4A/SLC24A2/ABCC8/KCNC1/SLC9A1/HCN1/SNAP25/SLC12A5/KCNA2/KCNS1/KCNH6/KCNA1/KCNB1/KCNC3/KLHL24/CAB39/KCNMB3/OXSR1/KCNK12/KCNQ5/HCN2/ATP1B3/KCNIP3/LRRC38/ATP1B1/KCNJ14/ATP1A3/ATP1A4/KCNC4/SLC9A5/KCNA6/MIR133A1/KCNJ11/EDN3/KCNS3/ATP1A1/SLC12A8/KCNRG/KCNS2/PKD2/SLC12A2 |
| GO:0006813 | KCNAB3/KCNT1/ANK3/KCNJ3/ATP4A/SLC24A2/ABCC8/KCNC1/SLC9A1/HCN1/SNAP25/CDKN1B/SLC12A5/KCNA2/KCNS1/KCNH6/KCNA1/ATF4/KCNB1/KCNC3/KLHL24/CAB39/KCNMB3/OXSR1/KCNK12/KCNQ5/HCN2/ATP1B3/KCNIP3/LRRC38/ATP1B1/KCNJ14/ATP1A3/ATP1A4/KCNC4/SLC9A5/KCNA6/MIR133A1/KCNJ11/EDN3/KCNS3/ATP1A1/SLC12A8/KCNRG/KCNS2/PKD2/SLC12A2 |
| GO:1990778 | ANK1/EPB41/CPLX1/ANK3/ROCK2/GAS6/GPR158/SNAP25/GOLPH3/ADAM22/STAC2/ACSL3/LYPLA1/AR/KCNB1/PREPL/EFR3A/STX1B/ARL13B/DENND4C/STX8/ATP1B3/KCNIP3/GRIN2A/EPB41L3/FAM126B/ATP1B1/CACNG2/PLEKHF1/EHD2/RAB11FIP2/RHOQ/EFCAB7/GRIP2/MYO5A/MPP5/ITGB1/SPTBN4/KCNJ11/IKBKB/TMEM150A/TESC/WDR19/PLS1/APPL1/GOLGA4/TNF/EGFR/SORBS1/MRAP2/EPHB2/BBS1 |

**Note:** GO, gene ontology.
